# Supplementary material for: STING inhibits LINE-1 retrotransposition through sorting ORF1p to lysosomes for degradation
Source: EMBO Rep. 2025 Aug 18;26(18):4607–30. doi: 10.1038/s44319-025-00551-0 (PMC12457603; doi:10.1038/s44319-025-00551-0)
Supplement: Supplementary file 1 — Appendix [file 44319_2025_551_MOESM1_ESM.pdf]

EMBOR-2024-60709V3

Appendix

STING inhibits LINE-1 retrotransposition through sorting ORF1p to lysosomes for degradation

Yu Huang, Fengwen Xu, Lingwa Wang, Shan Mei, Fei Zhao, Liming Wang, Yu Xie, Liang Wei, Yamei Hu, Zhao Gao, Tiffany Xue, Jugao Fang, Fei Guo,

Table of Content

Appendix figure S1…………………………………………………………………………..2
Appendix figure S2…………………………………………………………………………..3
Appendix figure S3…………………………………………………………………………..4
Appendix figure S4…………………………………………………………………………..5
Appendix figure S5…………………………………………………………………………..6
Appendix figure S6…………………………………………………………………………..7

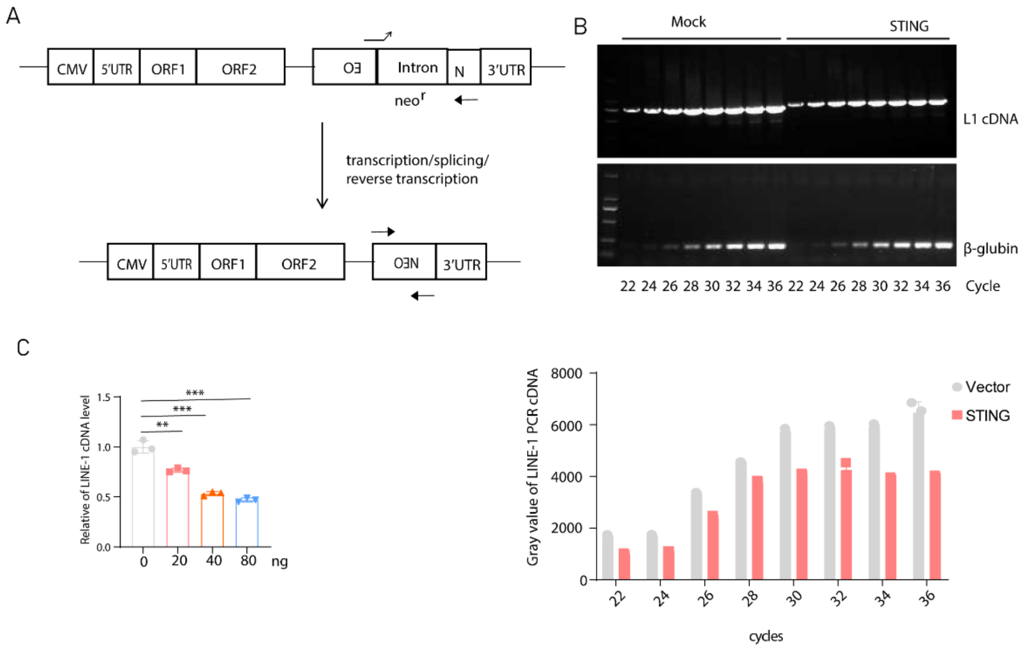

Appendix Figure S1. STING reduces the level of LINE-1 DNA.

(A) Positions of primers that were used to amplify CMV-L1-neo^{RT} DNA.

(B and C) HEK293T cells were co-transfected with the CMV-L1-neo^{RT} and STING-Flag DNA. Levels of the newly synthesized LINE-1 DNA were determined by semi-quantitative PCR (B) and quantitative PCR (C). Levels of β- globin DNA were measured as internal controls. (mean ± SEM; paired *t*-test). ns, no significant; *, P<0.05; **, P<0.01; ***, P<0.001.

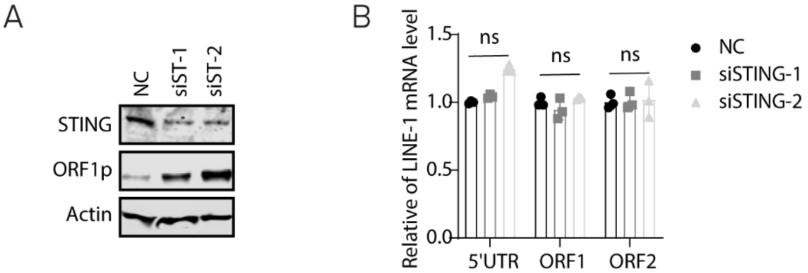

Appendix Figure S2. LINE-1 retrotransposition in THP1 cell with STING knockdown.

(A) siRNA was used to knock down endogenous STING in THP1 cells. Endogenous ORF1p was detected by Western blot.

(B) RT-qPCR was performed to measure endogenous mRNA levels of LINE-1 5'UTR, ORF1 and ORF2 in THP1 cells transfected with siRNA targeting STING. (mean ± SEM; paired *t*-test). ns, no significant.

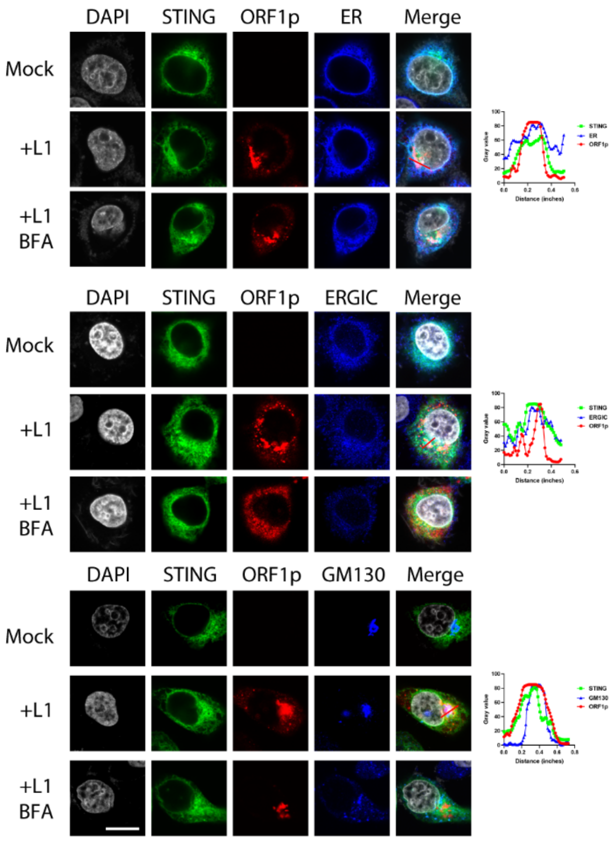

Appendix Figure S3. Subcellular localization of STING and ORF1p at ER (Calnexin), ERGIC (ERGIC53) and Golgi (GM130) in cGAS knockout HeLa cell line.

STING-EGFP and CMV-L1-neo^{RT} were co-transfected into cGAS knockout HeLa cells. 12 hours post transfection, cells were treated with BFA (Brefeldin A) (2 μM) for 20 hours

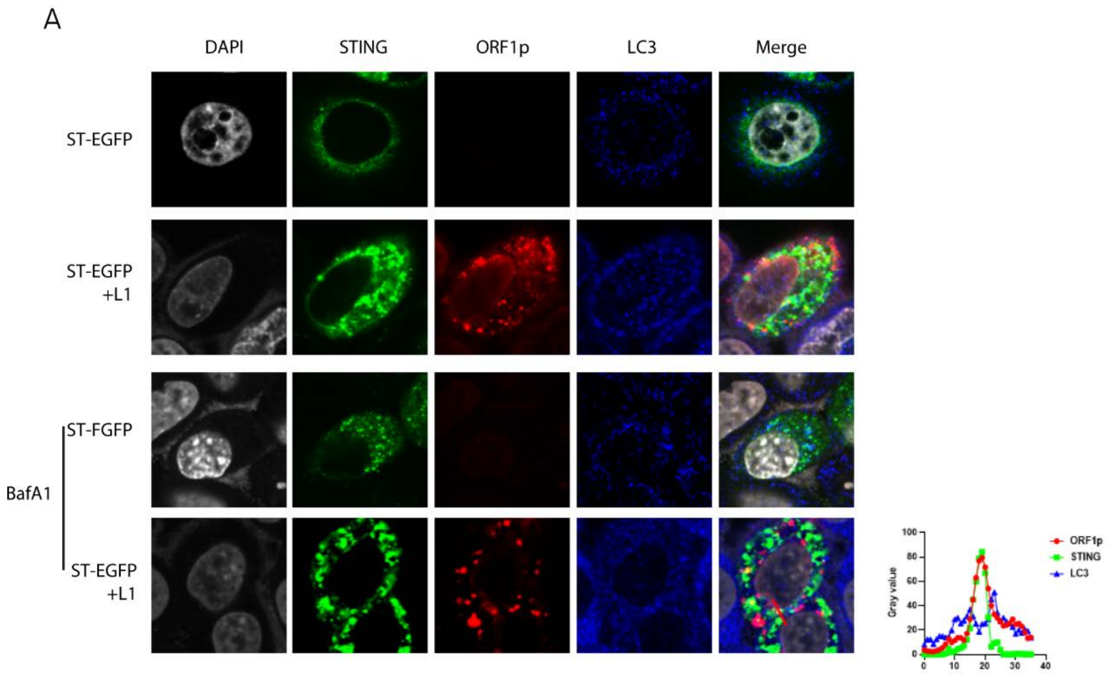

Appendix Figure S4. Subcellular localization of STING-EGFP and ORF1p with endogenous LC3.

Immunofluorescence confocal microscopy was performed to detect the subcellular localization of ORF1p and endogenous LC3 in STING-EGFP HeLa cells with BafA1 treatment. STING in green, ORF1p in red, LC3 in blue. ImageJ was used to analyze the co-localization. Scale bar, 10 μm.

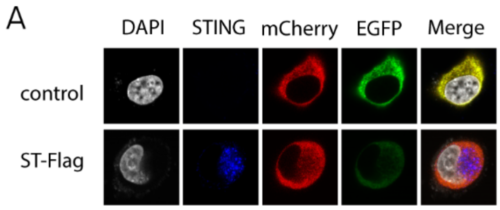

Appendix Figure S5. Immunofluorescence of EGFP-mCherry-ORF1 in HeLa cells stably expressing STING-Flag.

EGFP-mCherry-ORF1p plasmid was transfected into STING-Flag-expressing HeLa cells. 24 hours post-transfection, cells fixed with 4%PFA and immunofluorescence staining was performed to detect the subcellular location of ORF1p and STING-Flag.

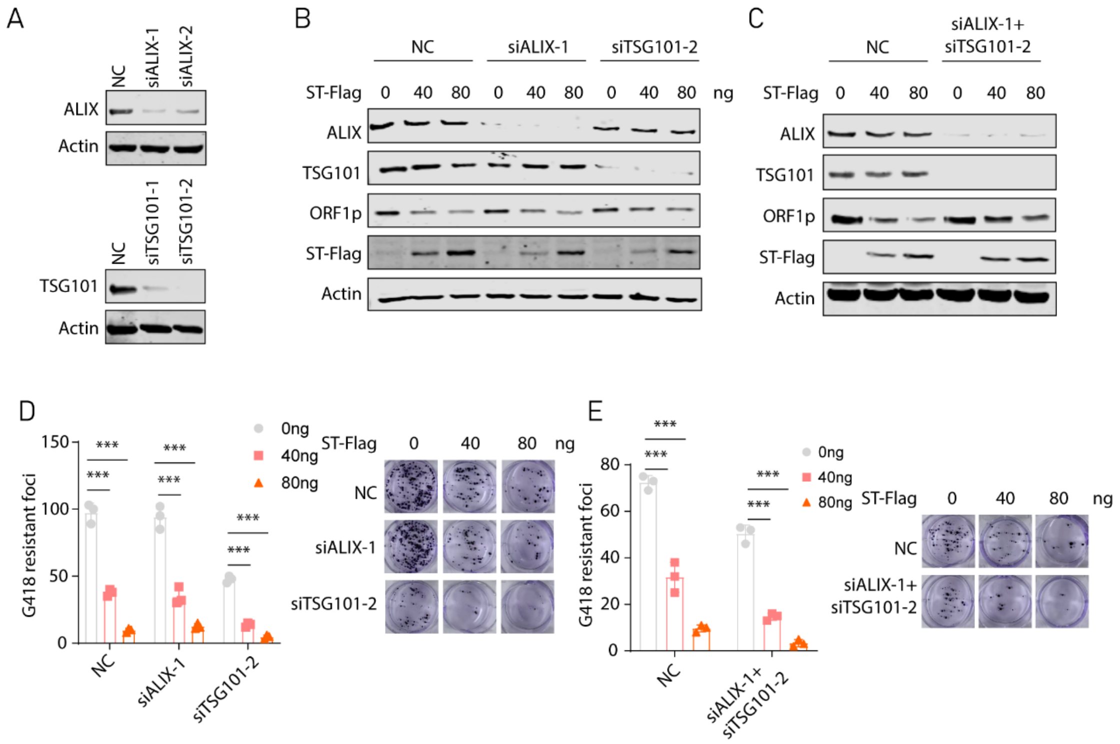

Appendix Figure S6. STING inhibition of LINE-1 retrotransposition is independent of ESCRT

(A) siRNA was used to knock down endogenous ALIX and TSG101 in Hela cells. Western-blot was performed at 48 hours post siRNA transfection.

(B) Western-blot was performed in siALIX or TSG101 knockdown HeLa cells which were co-transfected with CMV-L1-neo^{RT} DNA and STING-Flag plasmid.

(C) Western-blot was performed in siALIX and TSG101 double knockdown HeLa cells which were co-transfected with CMV-L1-neo^{RT} DNA and STING-Flag plasmid.

(D) CMV-L1-neo^{RT} colony assay was performed in ALIX or TSG101 knockdown HeLa cells which were co-transfected with CMV-L1-neo^{RT} DNA and STING-Flag plasmid. G418-resistant cell colonies were scored. The results of three independent experiments are presented in the bar graph (mean ± SEM; paired *t*-test). Compared with 0ng STING-Flag transfection, a significant difference was observed of 40ng ($p<0.001$) and 80ng ($p<0.001$), no matter in NC, siALIX or TSG101 knockdown group.

(E) CMV-L1-neo^{RT} colony assay was performed in ALIX and TSG101 double knockdown HeLa cells which were co-transfected with CMV-L1-neo^{RT} DNA and STING-Flag plasmid. G418-resistant cell colonies were scored. The results of three independent experiments are presented in the bar graph (mean ± SEM; paired *t*-test). Compared with 0ng STING-Flag transfection, a significant difference was observed of 40ng ($p<0.001$) and 80ng ($p<0.001$), no matter in NC or siALIX/TSG101 double knockdown group.
