# Supplementary figures and images for: STING inhibits LINE-1 retrotransposition through sorting ORF1p to lysosomes for degradation (part 1 of 4)
Source: EMBO Rep. 2025 Aug 18;26(18):4607–30. doi: 10.1038/s44319-025-00551-0 (PMC12457603; doi:10.1038/s44319-025-00551-0)

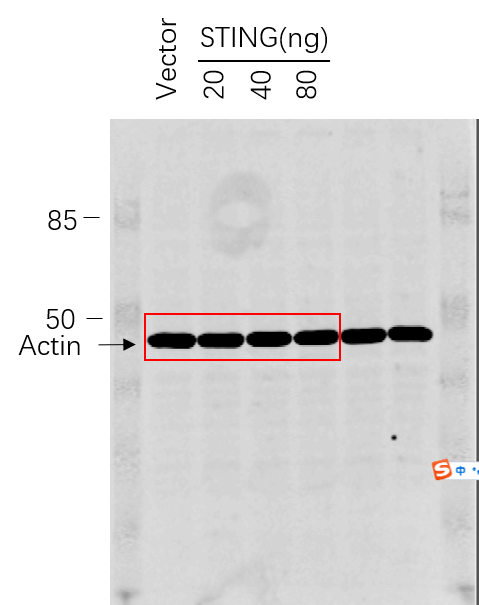

Supplement: Supplementary file 3 — Source data Fig. 1 [file 44319_2025_551_MOESM3_ESM.zip › Fig1/Fig 1E/Fig1E IB Actin.png]

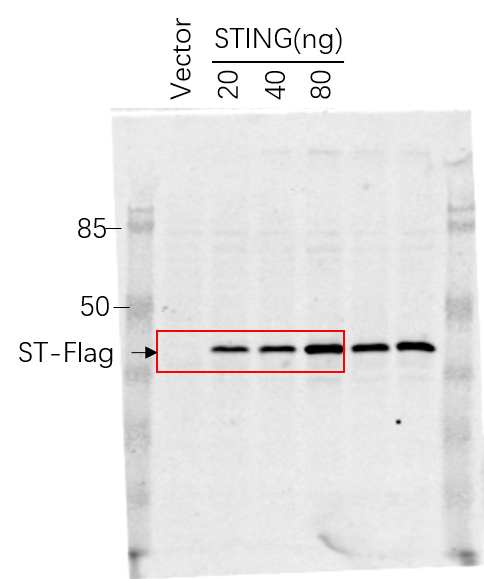

Supplement: Supplementary file 3 — Source data Fig. 1 [file 44319_2025_551_MOESM3_ESM.zip › Fig1/Fig 1E/Fig1E IB Flag.png]

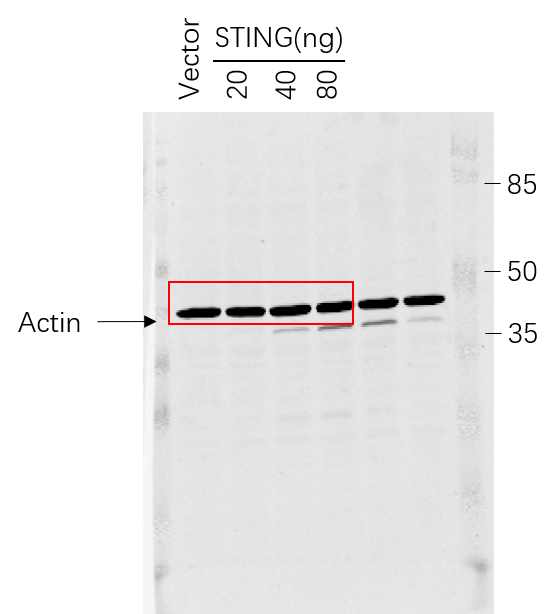

Supplement: Supplementary file 3 — Source data Fig. 1 [file 44319_2025_551_MOESM3_ESM.zip › Fig1/Fig 1F/Fig 1F IB Actin.png]

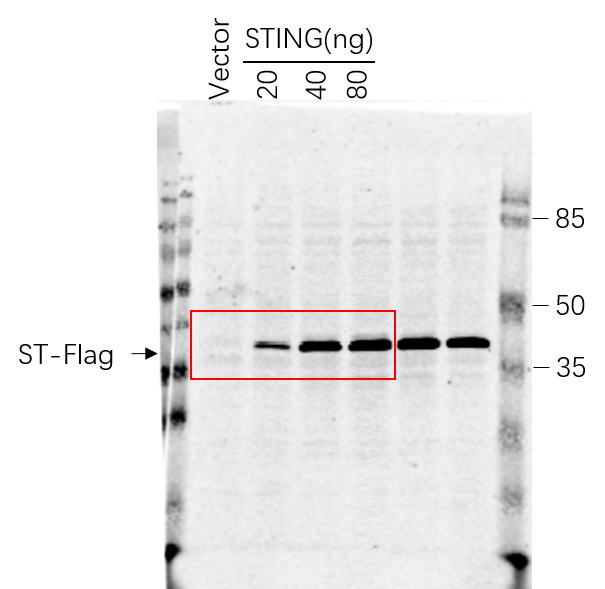

Supplement: Supplementary file 3 — Source data Fig. 1 [file 44319_2025_551_MOESM3_ESM.zip › Fig1/Fig 1F/Fig 1F IB Flag.png]

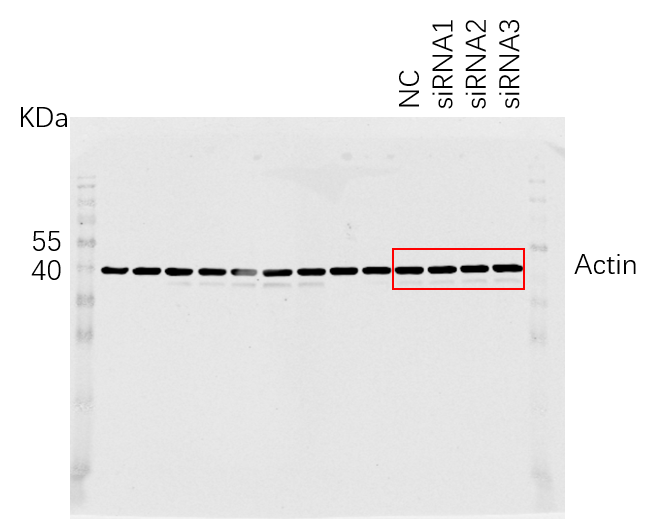

Supplement: Supplementary file 3 — Source data Fig. 1 [file 44319_2025_551_MOESM3_ESM.zip › Fig1/Fig 1H/Fig 1H IB actin.png]

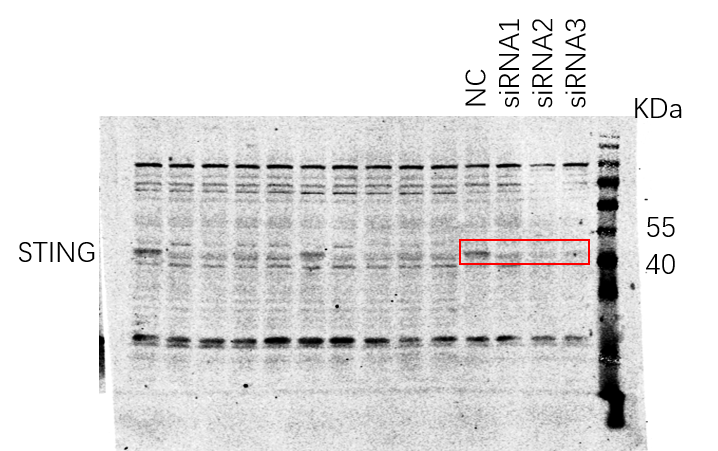

Supplement: Supplementary file 3 — Source data Fig. 1 [file 44319_2025_551_MOESM3_ESM.zip › Fig1/Fig 1H/Fig 1H IB STING.png]

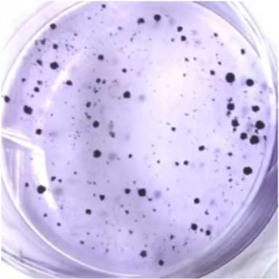

Supplement: Supplementary file 3 — Source data Fig. 1 [file 44319_2025_551_MOESM3_ESM.zip › Fig1/Fig 1I/Fig1I Image/Fig 1I NC.tif]

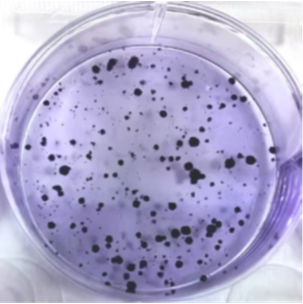

Supplement: Supplementary file 3 — Source data Fig. 1 [file 44319_2025_551_MOESM3_ESM.zip › Fig1/Fig 1I/Fig1I Image/Fig 1I siSTING-1.tif]

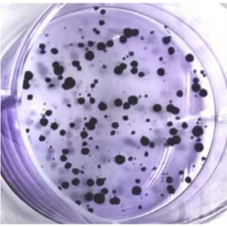

Supplement: Supplementary file 3 — Source data Fig. 1 [file 44319_2025_551_MOESM3_ESM.zip › Fig1/Fig 1I/Fig1I Image/Fig 1I siSTING-2.tif]

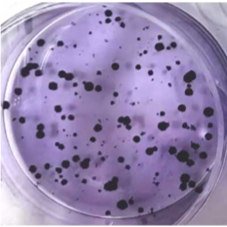

Supplement: Supplementary file 3 — Source data Fig. 1 [file 44319_2025_551_MOESM3_ESM.zip › Fig1/Fig 1I/Fig1I Image/Fig 1I siSTING-3.tif]

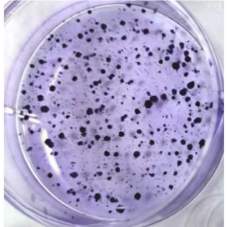

Supplement: Supplementary file 3 — Source data Fig. 1 [file 44319_2025_551_MOESM3_ESM.zip › Fig1/Fig 1J/Fig1J image/Fig 1J NC.tif]

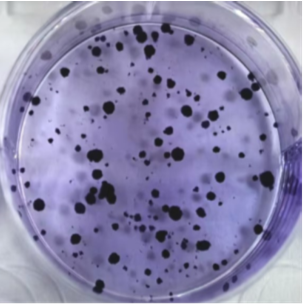

Supplement: Supplementary file 3 — Source data Fig. 1 [file 44319_2025_551_MOESM3_ESM.zip › Fig1/Fig 1J/Fig1J image/Fig 1J siSTING-1.tif]

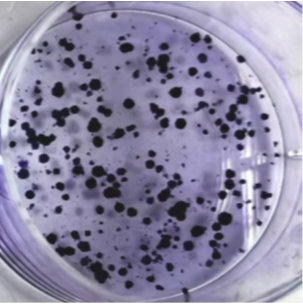

Supplement: Supplementary file 3 — Source data Fig. 1 [file 44319_2025_551_MOESM3_ESM.zip › Fig1/Fig 1J/Fig1J image/Fig 1J siSTING-2.tif]

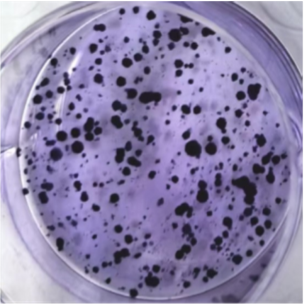

Supplement: Supplementary file 3 — Source data Fig. 1 [file 44319_2025_551_MOESM3_ESM.zip › Fig1/Fig 1J/Fig1J image/Fig 1J siSTING-3.tif]

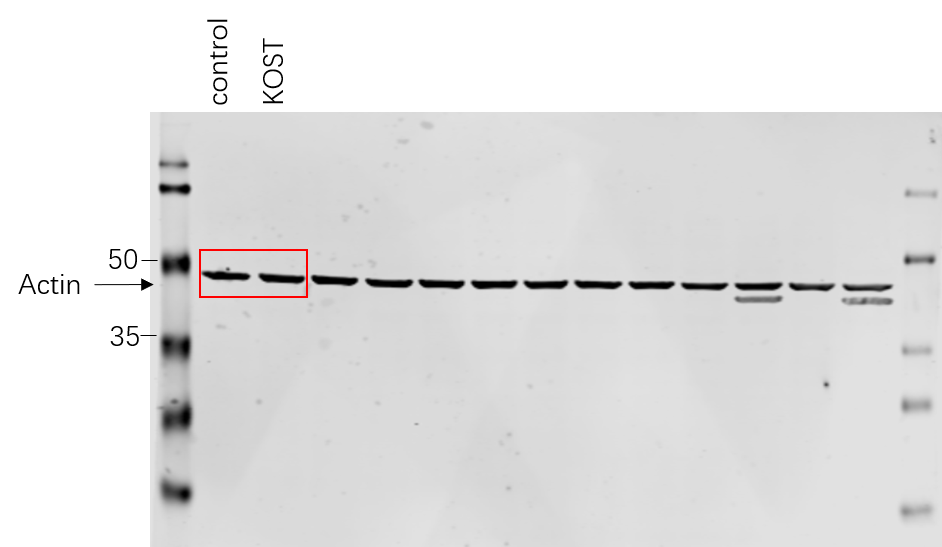

Supplement: Supplementary file 3 — Source data Fig. 1 [file 44319_2025_551_MOESM3_ESM.zip › Fig1/Fig 1K/Fig 1K Actin.png]

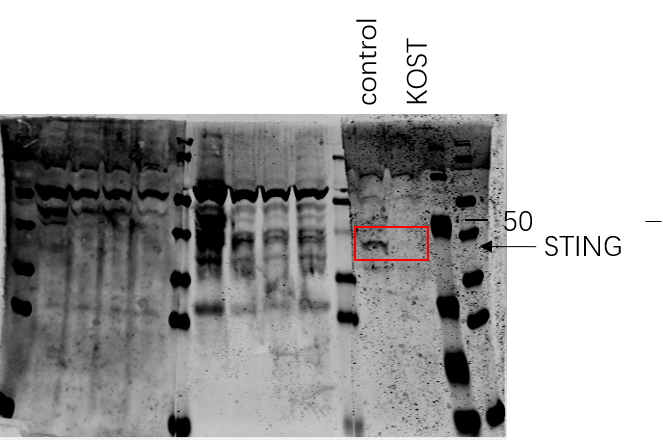

Supplement: Supplementary file 3 — Source data Fig. 1 [file 44319_2025_551_MOESM3_ESM.zip › Fig1/Fig 1K/Fig 1K STING.png]

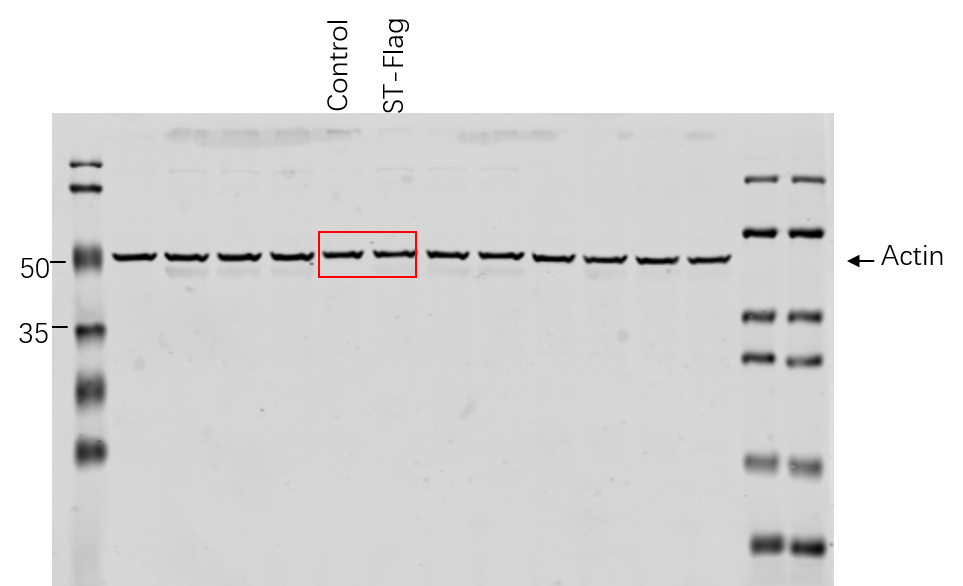

Supplement: Supplementary file 3 — Source data Fig. 1 [file 44319_2025_551_MOESM3_ESM.zip › Fig1/Fig1 L/Fig1 L IB Actin.png]

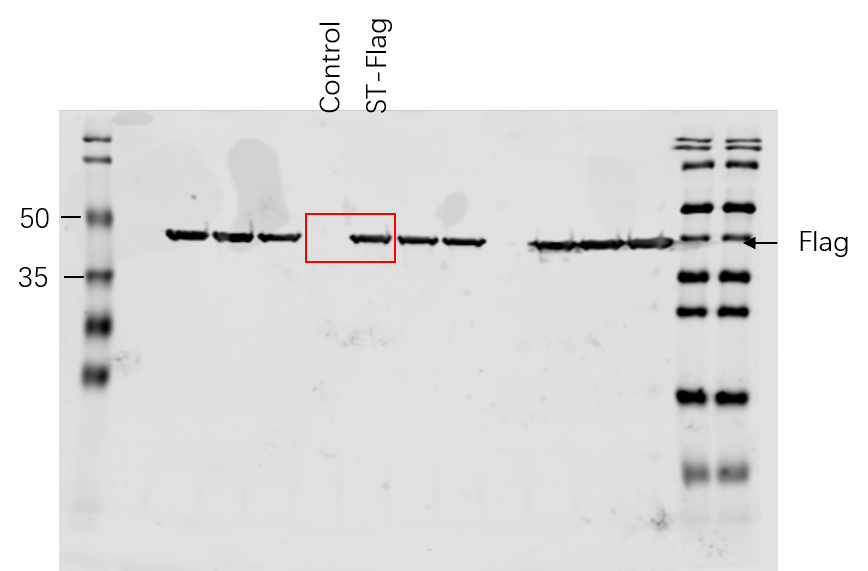

Supplement: Supplementary file 3 — Source data Fig. 1 [file 44319_2025_551_MOESM3_ESM.zip › Fig1/Fig1 L/Fig1 L IB Flag.png]

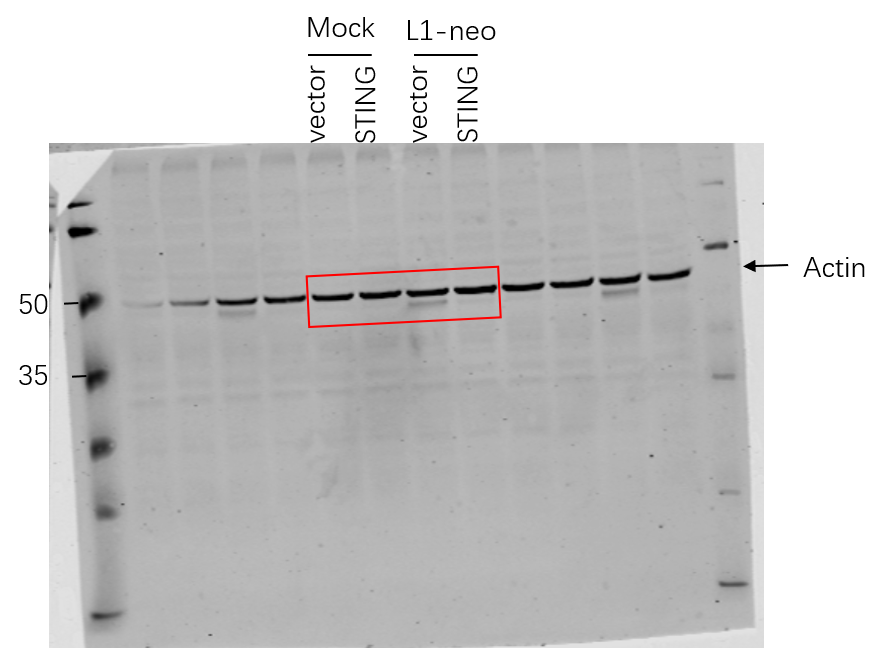

Supplement: Supplementary file 4 — Source data Fig. 2 [file 44319_2025_551_MOESM4_ESM.zip › Fig2/Fig 2A/Fig 2A IB Actin.png]

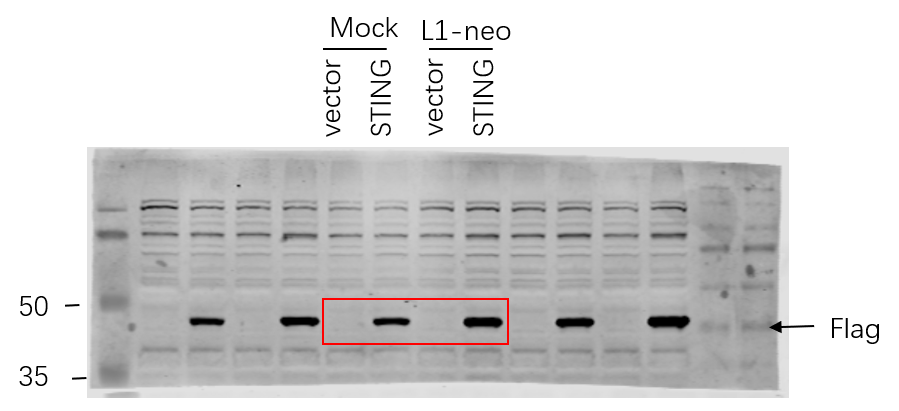

Supplement: Supplementary file 4 — Source data Fig. 2 [file 44319_2025_551_MOESM4_ESM.zip › Fig2/Fig 2A/Fig 2A IB Flag.png]

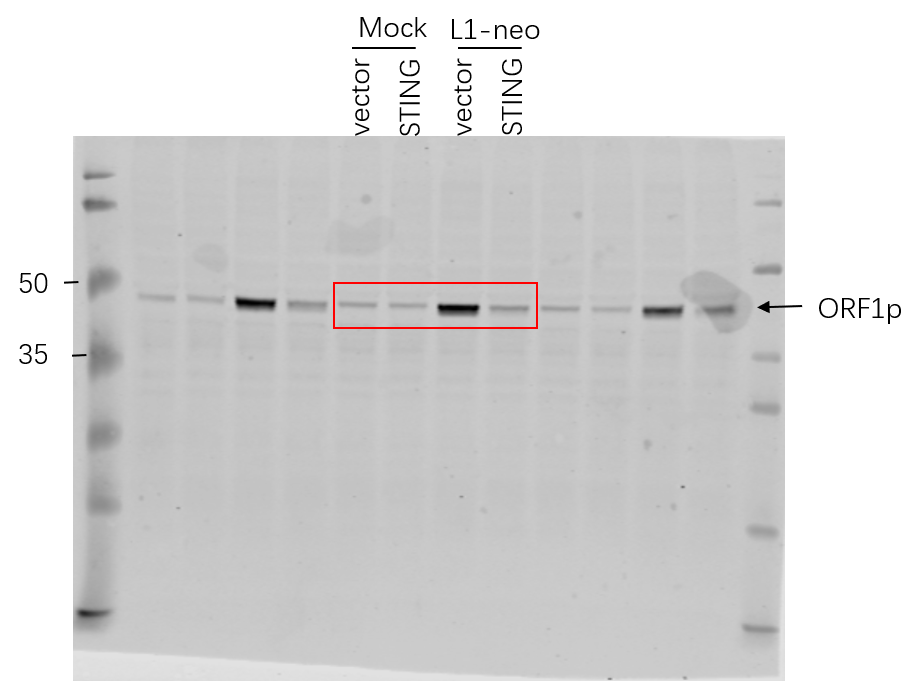

Supplement: Supplementary file 4 — Source data Fig. 2 [file 44319_2025_551_MOESM4_ESM.zip › Fig2/Fig 2A/Fig 2A IB ORF1p.png]

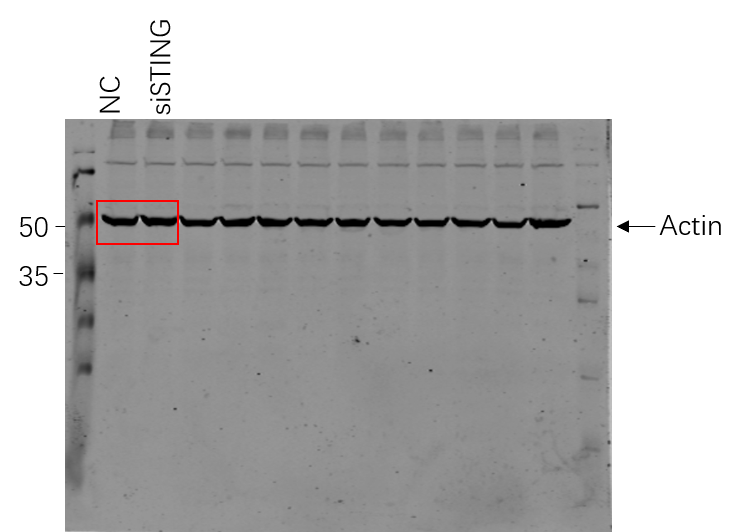

Supplement: Supplementary file 4 — Source data Fig. 2 [file 44319_2025_551_MOESM4_ESM.zip › Fig2/Fig 2B/Fig2B IB Actin.png]

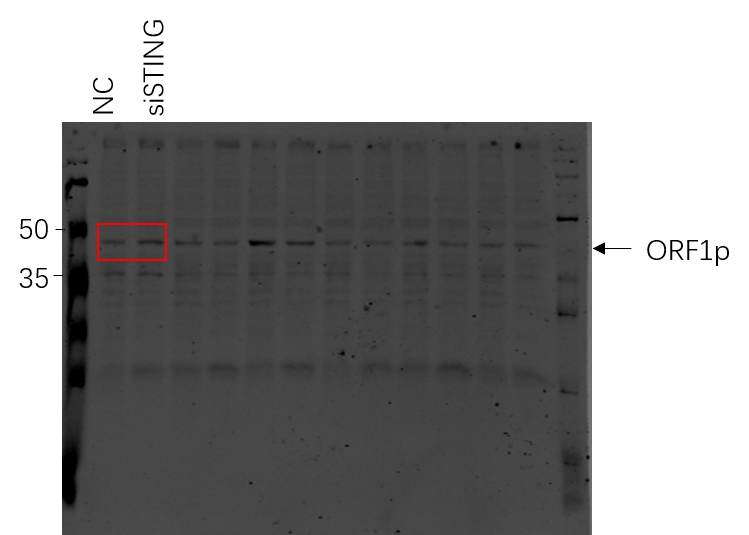

Supplement: Supplementary file 4 — Source data Fig. 2 [file 44319_2025_551_MOESM4_ESM.zip › Fig2/Fig 2B/Fig2B IB ORF1p.png]

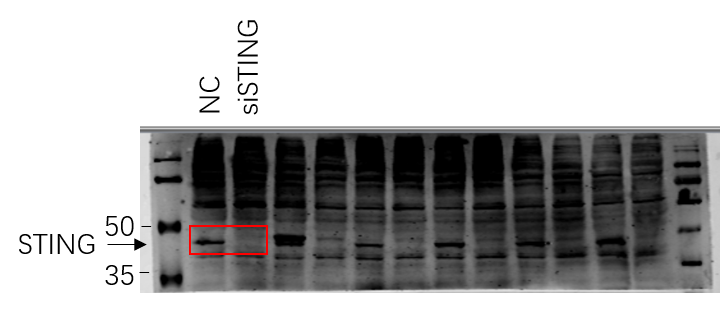

Supplement: Supplementary file 4 — Source data Fig. 2 [file 44319_2025_551_MOESM4_ESM.zip › Fig2/Fig 2B/Fig2B IB STING.png]

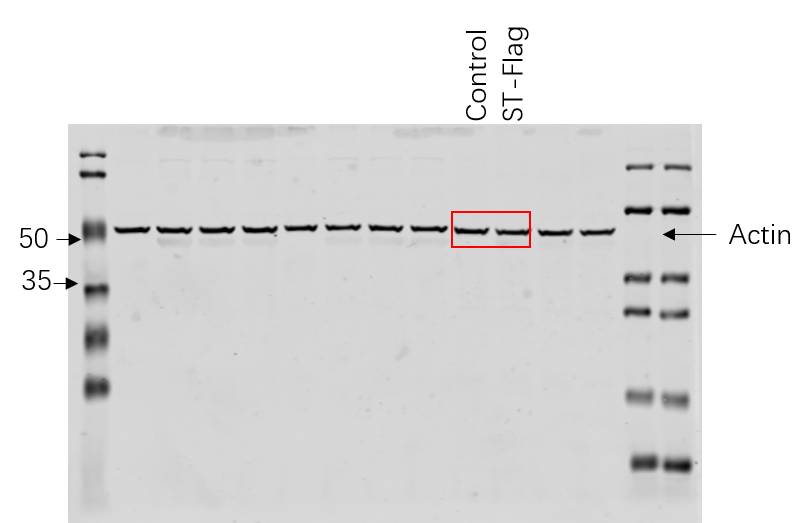

Supplement: Supplementary file 4 — Source data Fig. 2 [file 44319_2025_551_MOESM4_ESM.zip › Fig2/Fig 2E/Fig2E IB Actin.png]

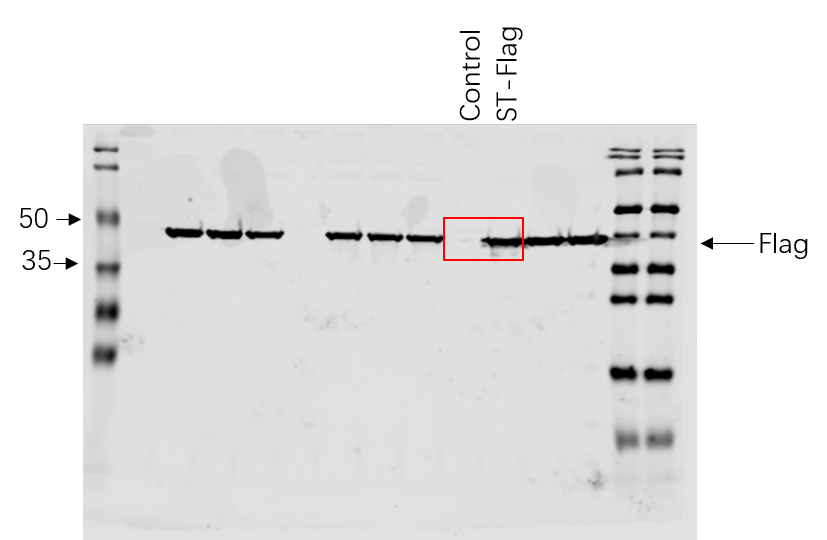

Supplement: Supplementary file 4 — Source data Fig. 2 [file 44319_2025_551_MOESM4_ESM.zip › Fig2/Fig 2E/Fig2E IB Flag.png]

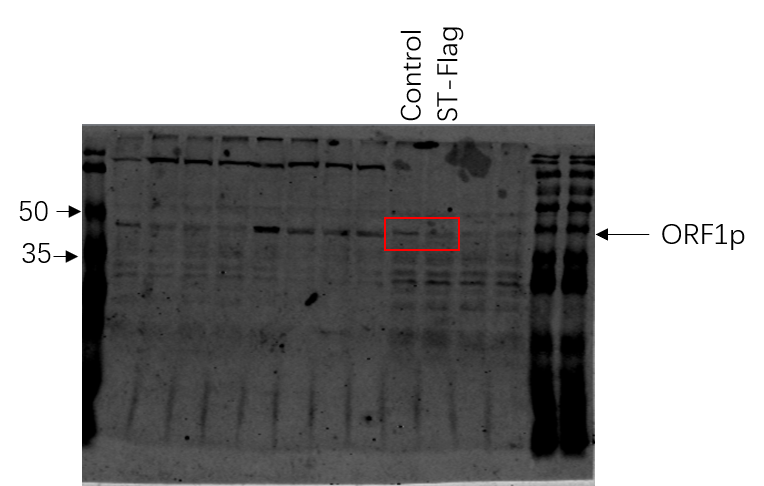

Supplement: Supplementary file 4 — Source data Fig. 2 [file 44319_2025_551_MOESM4_ESM.zip › Fig2/Fig 2E/Fig2E IB ORF1p.png]

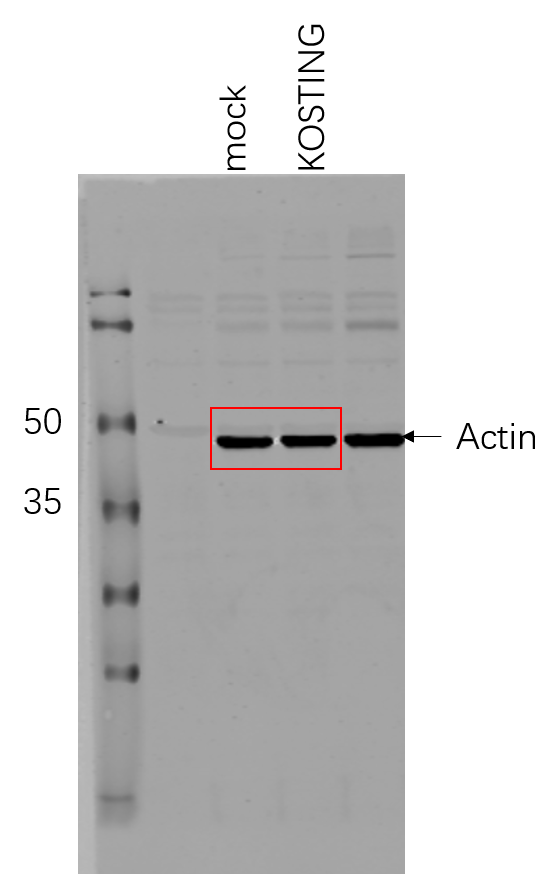

Supplement: Supplementary file 4 — Source data Fig. 2 [file 44319_2025_551_MOESM4_ESM.zip › Fig2/Fig 2G/Fig 2G IB Actin.png]

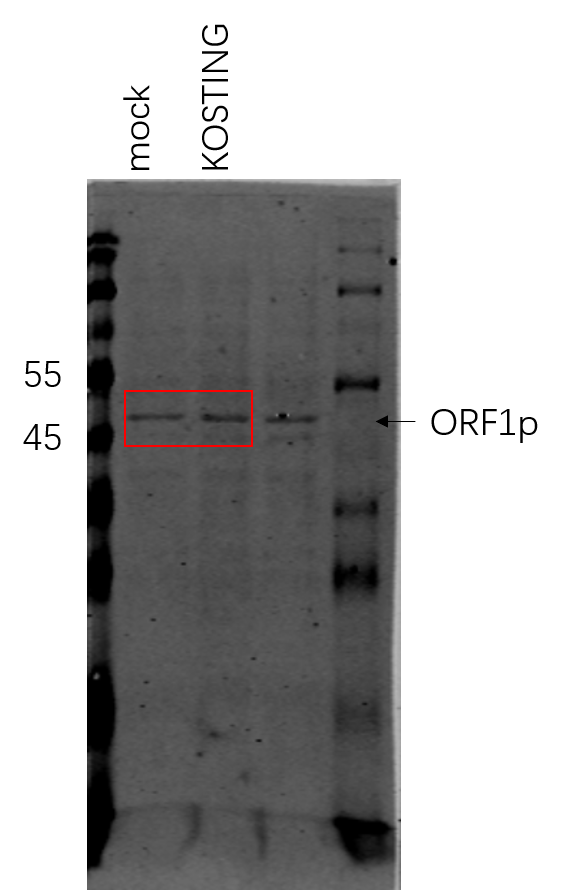

Supplement: Supplementary file 4 — Source data Fig. 2 [file 44319_2025_551_MOESM4_ESM.zip › Fig2/Fig 2G/Fig 2G IB ORF1p.png]

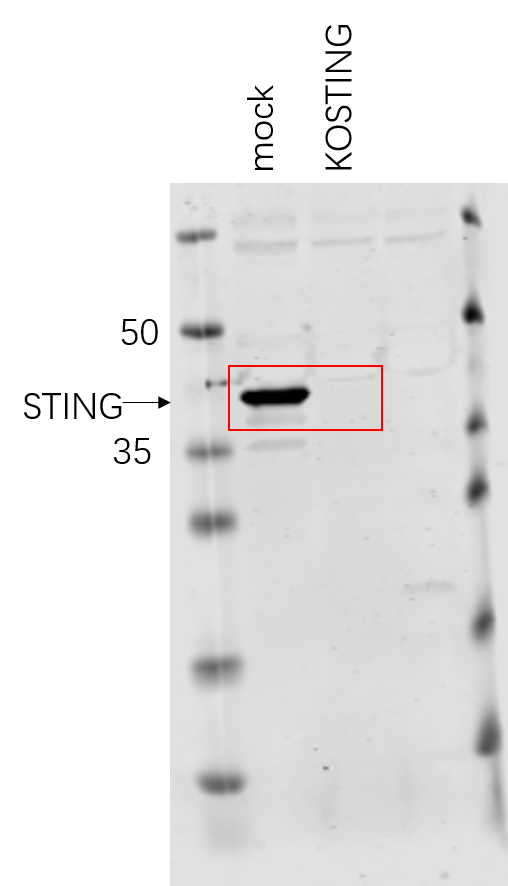

Supplement: Supplementary file 4 — Source data Fig. 2 [file 44319_2025_551_MOESM4_ESM.zip › Fig2/Fig 2G/Fig 2G IB STING.png]

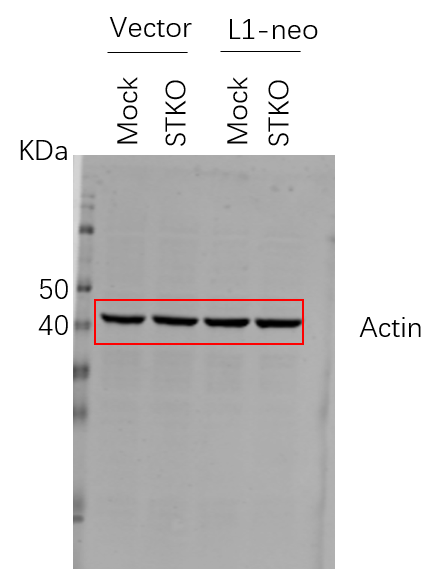

Supplement: Supplementary file 4 — Source data Fig. 2 [file 44319_2025_551_MOESM4_ESM.zip › Fig2/Fig 2J/Fig2I IB Actin.png]

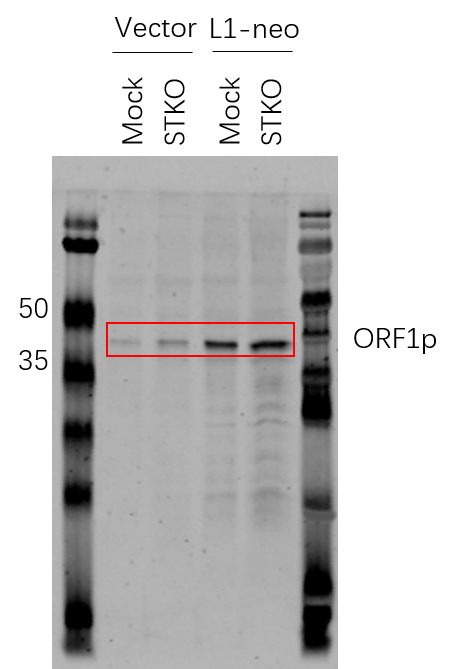

Supplement: Supplementary file 4 — Source data Fig. 2 [file 44319_2025_551_MOESM4_ESM.zip › Fig2/Fig 2J/Fig2I IB ORF1p.png]

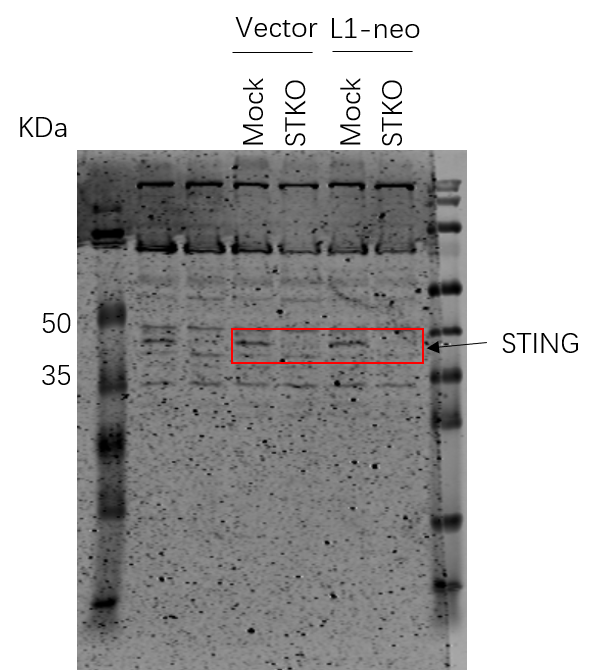

Supplement: Supplementary file 4 — Source data Fig. 2 [file 44319_2025_551_MOESM4_ESM.zip › Fig2/Fig 2J/Fig2I IB STING.png]

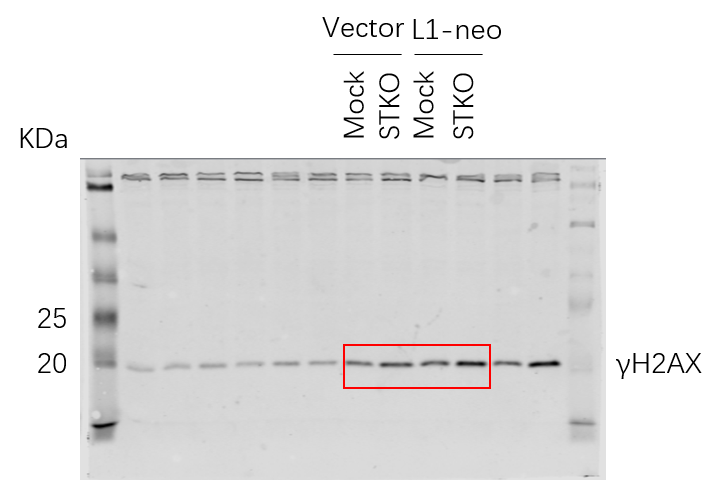

Supplement: Supplementary file 4 — Source data Fig. 2 [file 44319_2025_551_MOESM4_ESM.zip › Fig2/Fig 2J/Fig2I IB γH2AX.png]

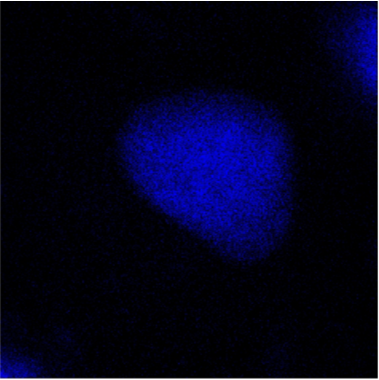

Supplement: Supplementary file 4 — Source data Fig. 2 [file 44319_2025_551_MOESM4_ESM.zip › Fig2/Fig 2K/L1neo mock/DAPI.tif]

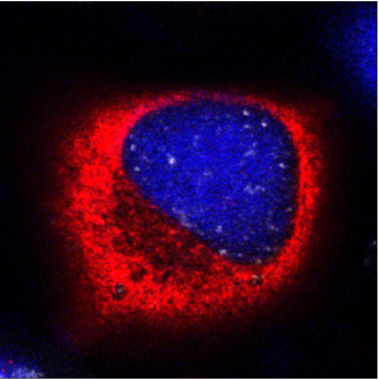

Supplement: Supplementary file 4 — Source data Fig. 2 [file 44319_2025_551_MOESM4_ESM.zip › Fig2/Fig 2K/L1neo mock/Merge.tif]

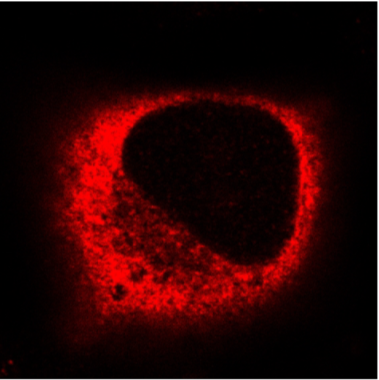

Supplement: Supplementary file 4 — Source data Fig. 2 [file 44319_2025_551_MOESM4_ESM.zip › Fig2/Fig 2K/L1neo mock/ORF1p.tif]

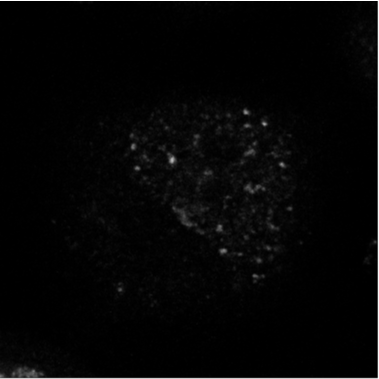

Supplement: Supplementary file 4 — Source data Fig. 2 [file 44319_2025_551_MOESM4_ESM.zip › Fig2/Fig 2K/L1neo mock/γH2AX.tif]

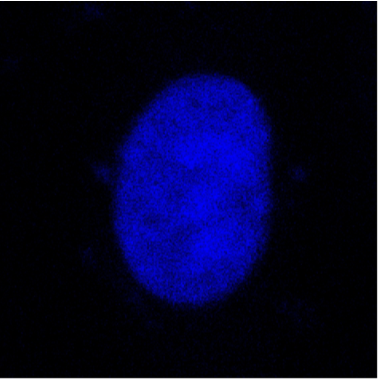

Supplement: Supplementary file 4 — Source data Fig. 2 [file 44319_2025_551_MOESM4_ESM.zip › Fig2/Fig 2K/L1neo STKO/DAPI.tif]

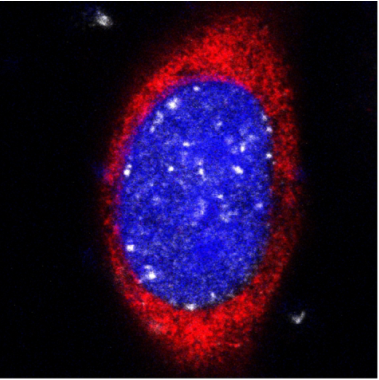

Supplement: Supplementary file 4 — Source data Fig. 2 [file 44319_2025_551_MOESM4_ESM.zip › Fig2/Fig 2K/L1neo STKO/Merge.tif]

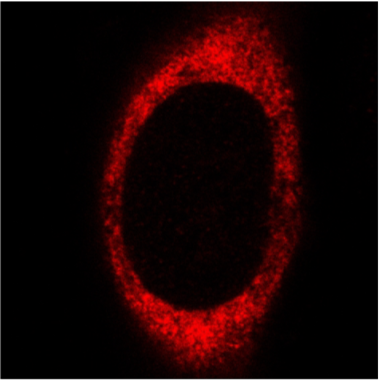

Supplement: Supplementary file 4 — Source data Fig. 2 [file 44319_2025_551_MOESM4_ESM.zip › Fig2/Fig 2K/L1neo STKO/ORF1p.tif]

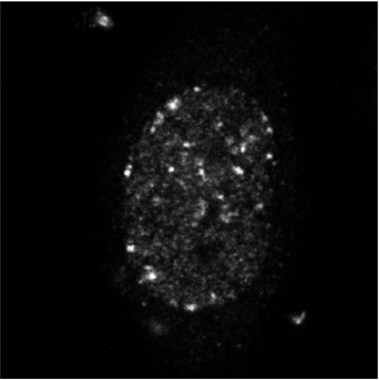

Supplement: Supplementary file 4 — Source data Fig. 2 [file 44319_2025_551_MOESM4_ESM.zip › Fig2/Fig 2K/L1neo STKO/γH2AX.tif]

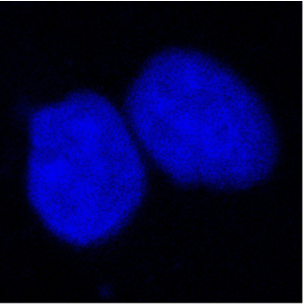

Supplement: Supplementary file 4 — Source data Fig. 2 [file 44319_2025_551_MOESM4_ESM.zip › Fig2/Fig 2K/Vector mock/DAPI.tif]

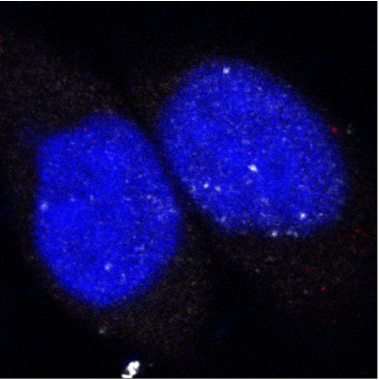

Supplement: Supplementary file 4 — Source data Fig. 2 [file 44319_2025_551_MOESM4_ESM.zip › Fig2/Fig 2K/Vector mock/merge.tif]

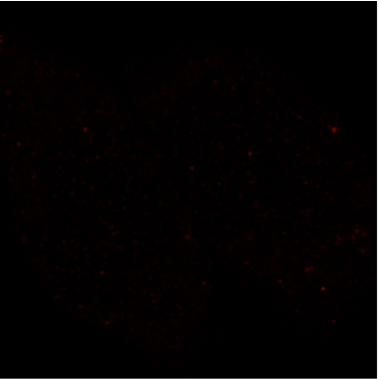

Supplement: Supplementary file 4 — Source data Fig. 2 [file 44319_2025_551_MOESM4_ESM.zip › Fig2/Fig 2K/Vector mock/ORF1p.tif]

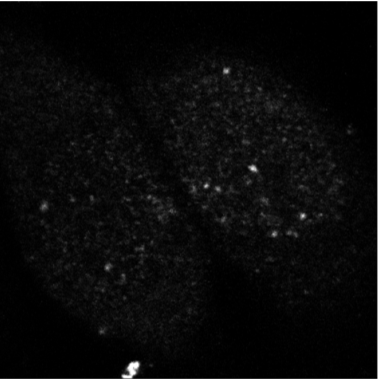

Supplement: Supplementary file 4 — Source data Fig. 2 [file 44319_2025_551_MOESM4_ESM.zip › Fig2/Fig 2K/Vector mock/γH2AX.tif]

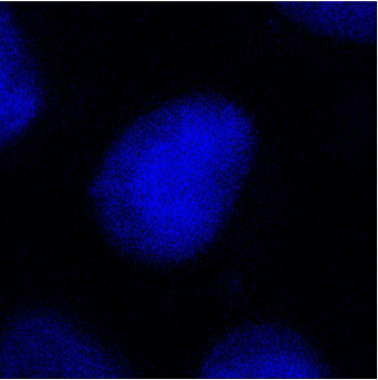

Supplement: Supplementary file 4 — Source data Fig. 2 [file 44319_2025_551_MOESM4_ESM.zip › Fig2/Fig 2K/Vector STKO/DAPI.tif]

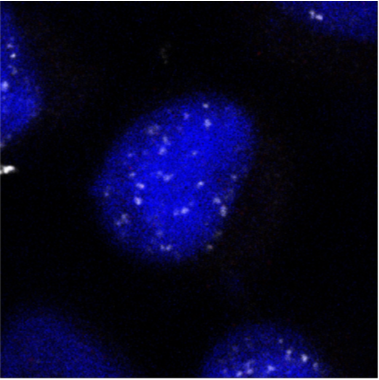

Supplement: Supplementary file 4 — Source data Fig. 2 [file 44319_2025_551_MOESM4_ESM.zip › Fig2/Fig 2K/Vector STKO/Merge.tif]

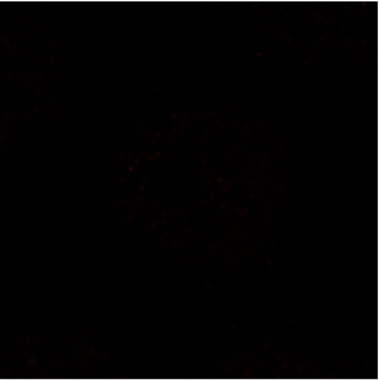

Supplement: Supplementary file 4 — Source data Fig. 2 [file 44319_2025_551_MOESM4_ESM.zip › Fig2/Fig 2K/Vector STKO/ORF1p.tif]

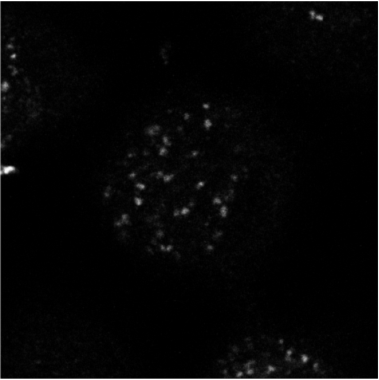

Supplement: Supplementary file 4 — Source data Fig. 2 [file 44319_2025_551_MOESM4_ESM.zip › Fig2/Fig 2K/Vector STKO/γH2AX.tif]

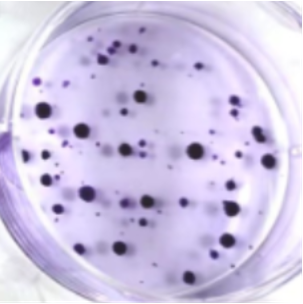

Supplement: Supplementary file 5 — Source data Fig. 3 [file 44319_2025_551_MOESM5_ESM.zip › Fig3/Fig3A/Fig3A image/cGASKO 40ng.tif]

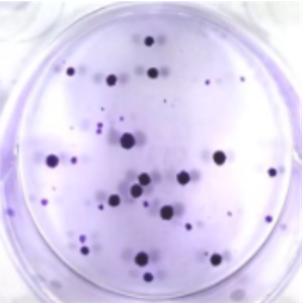

Supplement: Supplementary file 5 — Source data Fig. 3 [file 44319_2025_551_MOESM5_ESM.zip › Fig3/Fig3A/Fig3A image/cGASKO 80ng.tif]

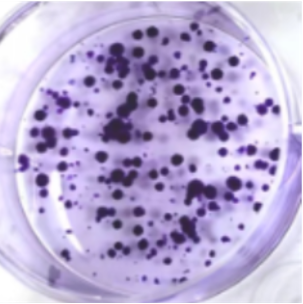

Supplement: Supplementary file 5 — Source data Fig. 3 [file 44319_2025_551_MOESM5_ESM.zip › Fig3/Fig3A/Fig3A image/cGASKO Vector.tif]

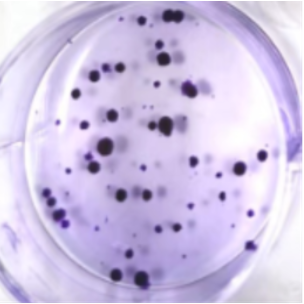

Supplement: Supplementary file 5 — Source data Fig. 3 [file 44319_2025_551_MOESM5_ESM.zip › Fig3/Fig3A/Fig3A image/V2 40ng.tif]

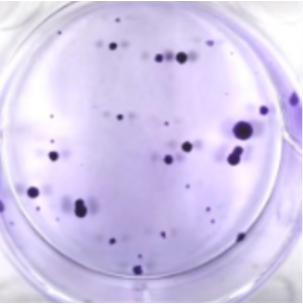

Supplement: Supplementary file 5 — Source data Fig. 3 [file 44319_2025_551_MOESM5_ESM.zip › Fig3/Fig3A/Fig3A image/V2 80ng.tif]

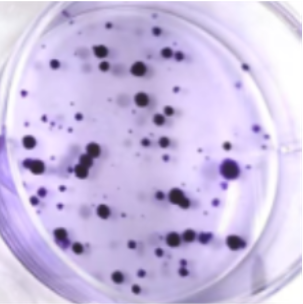

Supplement: Supplementary file 5 — Source data Fig. 3 [file 44319_2025_551_MOESM5_ESM.zip › Fig3/Fig3A/Fig3A image/V2 Vector.tif]

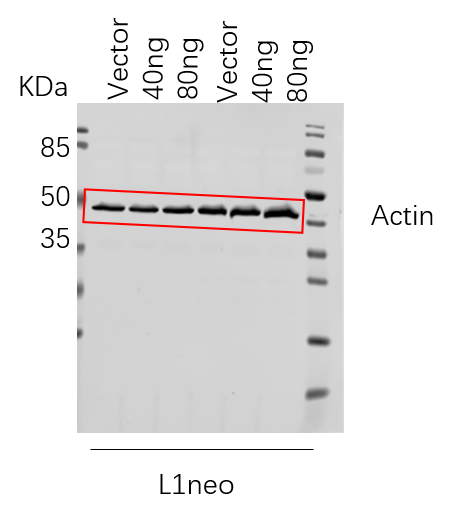

Supplement: Supplementary file 5 — Source data Fig. 3 [file 44319_2025_551_MOESM5_ESM.zip › Fig3/Fig3B/Fig 3B L1neo IB Actin.png]

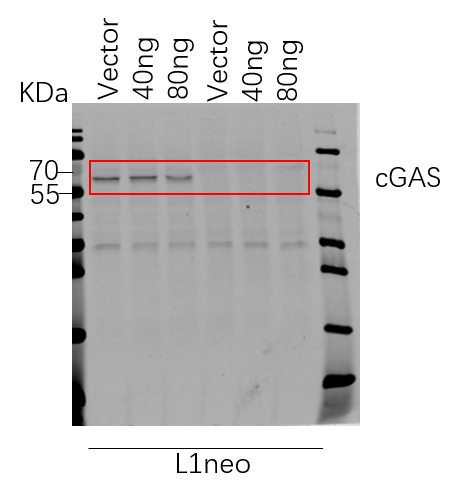

Supplement: Supplementary file 5 — Source data Fig. 3 [file 44319_2025_551_MOESM5_ESM.zip › Fig3/Fig3B/Fig 3B L1neo IB cGAS.png]

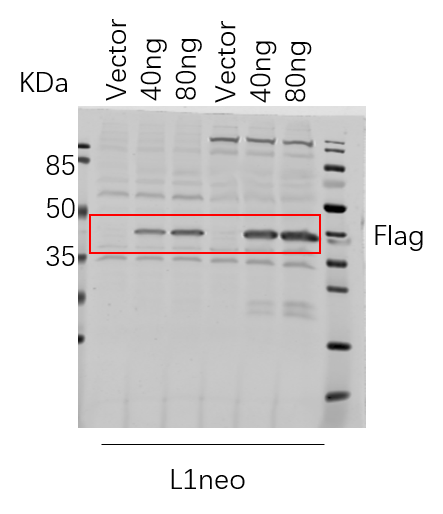

Supplement: Supplementary file 5 — Source data Fig. 3 [file 44319_2025_551_MOESM5_ESM.zip › Fig3/Fig3B/Fig 3B L1neo IB Flag.png]

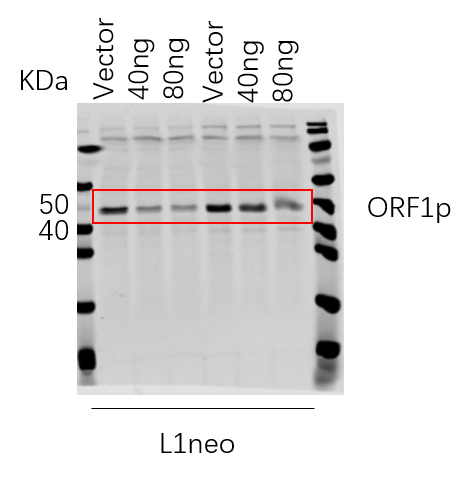

Supplement: Supplementary file 5 — Source data Fig. 3 [file 44319_2025_551_MOESM5_ESM.zip › Fig3/Fig3B/Fig 3B L1neo IB ORF1p.png]

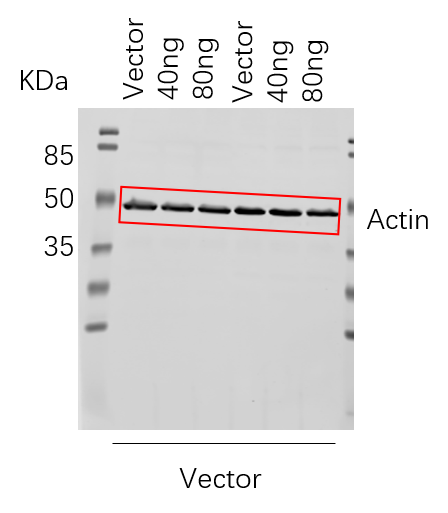

Supplement: Supplementary file 5 — Source data Fig. 3 [file 44319_2025_551_MOESM5_ESM.zip › Fig3/Fig3B/Fig 3B Vector IB Actin.png]

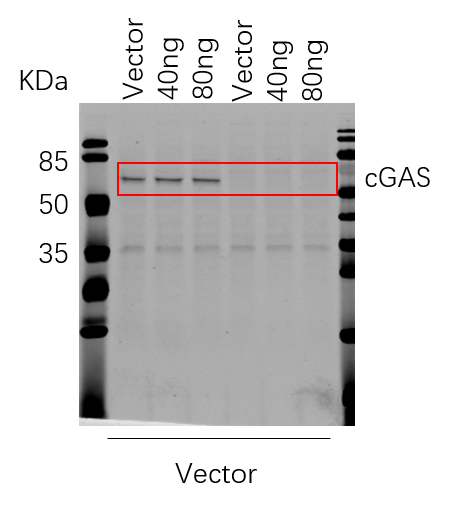

Supplement: Supplementary file 5 — Source data Fig. 3 [file 44319_2025_551_MOESM5_ESM.zip › Fig3/Fig3B/Fig 3B Vector IB cGAS.png]

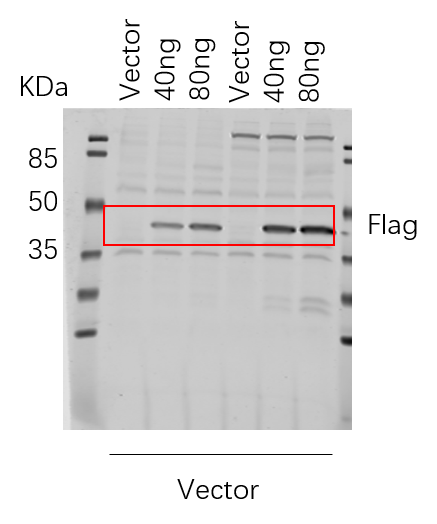

Supplement: Supplementary file 5 — Source data Fig. 3 [file 44319_2025_551_MOESM5_ESM.zip › Fig3/Fig3B/Fig 3B Vector IB Flag.png]

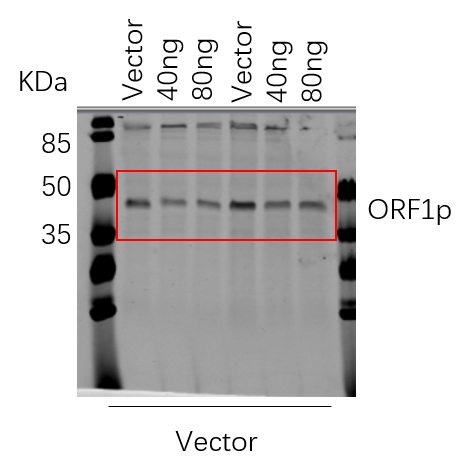

Supplement: Supplementary file 5 — Source data Fig. 3 [file 44319_2025_551_MOESM5_ESM.zip › Fig3/Fig3B/Fig 3B Vector IB ORF1p.png]

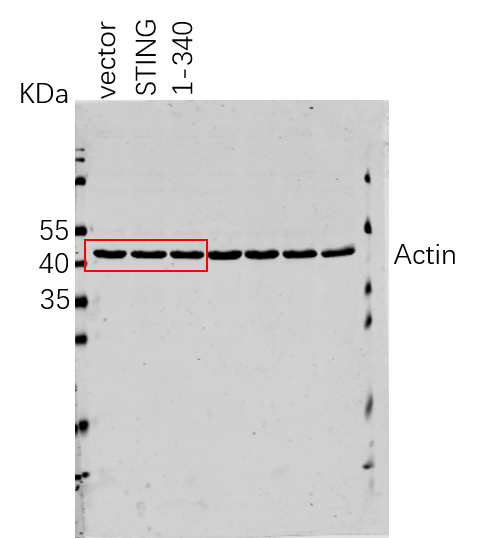

Supplement: Supplementary file 5 — Source data Fig. 3 [file 44319_2025_551_MOESM5_ESM.zip › Fig3/Fig3D/Fig3D IB Actin.png]

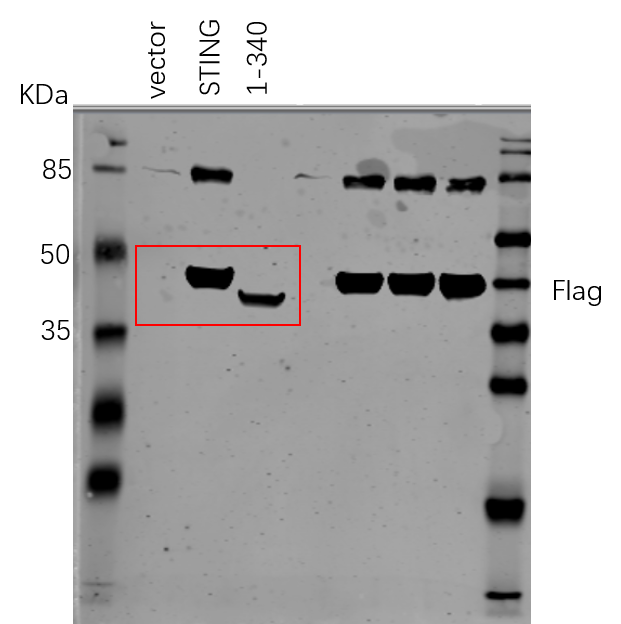

Supplement: Supplementary file 5 — Source data Fig. 3 [file 44319_2025_551_MOESM5_ESM.zip › Fig3/Fig3D/Fig3D IB Flag.png]

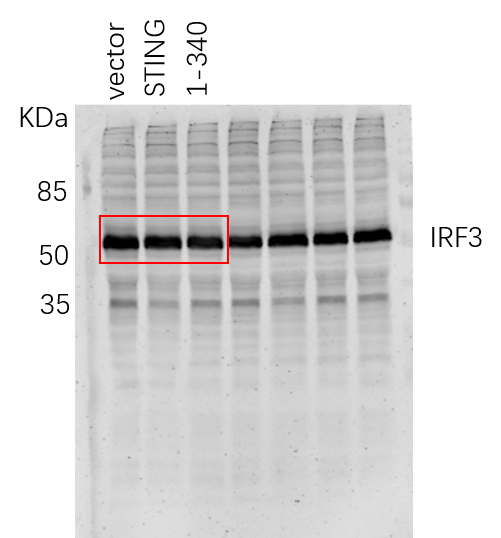

Supplement: Supplementary file 5 — Source data Fig. 3 [file 44319_2025_551_MOESM5_ESM.zip › Fig3/Fig3D/Fig3D IB IRF3.png]

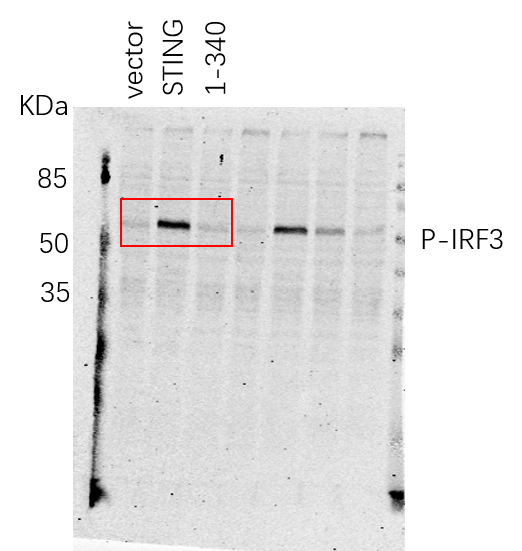

Supplement: Supplementary file 5 — Source data Fig. 3 [file 44319_2025_551_MOESM5_ESM.zip › Fig3/Fig3D/Fig3D IB pIRF3.png]

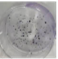

Supplement: Supplementary file 5 — Source data Fig. 3 [file 44319_2025_551_MOESM5_ESM.zip › Fig3/Fig3E/Fig3E image/1-340.tif]

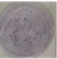

Supplement: Supplementary file 5 — Source data Fig. 3 [file 44319_2025_551_MOESM5_ESM.zip › Fig3/Fig3E/Fig3E image/mock.tif]

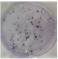

Supplement: Supplementary file 5 — Source data Fig. 3 [file 44319_2025_551_MOESM5_ESM.zip › Fig3/Fig3E/Fig3E image/STING.tif]

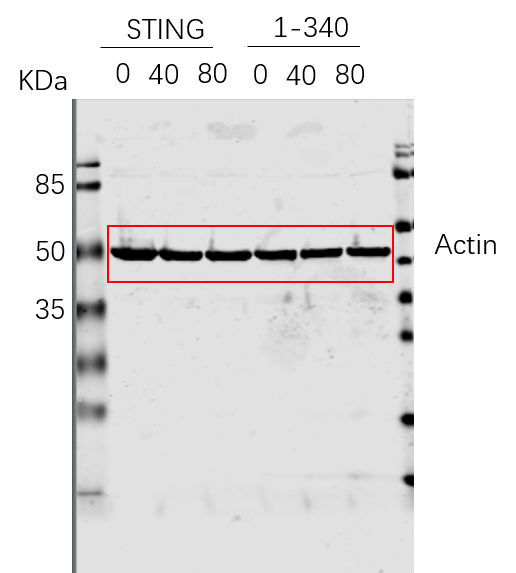

Supplement: Supplementary file 5 — Source data Fig. 3 [file 44319_2025_551_MOESM5_ESM.zip › Fig3/Fig3F/Fig3F IB Actin.png]

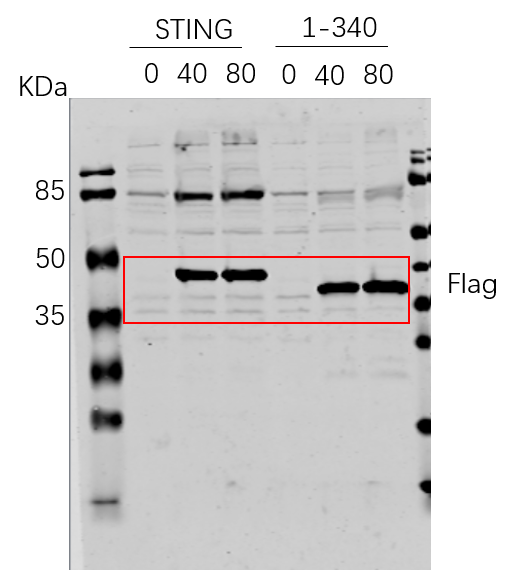

Supplement: Supplementary file 5 — Source data Fig. 3 [file 44319_2025_551_MOESM5_ESM.zip › Fig3/Fig3F/Fig3F IB Flag.png]

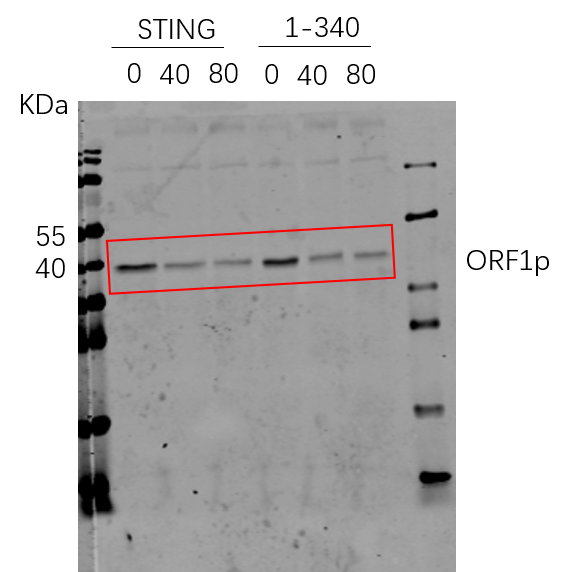

Supplement: Supplementary file 5 — Source data Fig. 3 [file 44319_2025_551_MOESM5_ESM.zip › Fig3/Fig3F/Fig3F IB ORF1p.png]

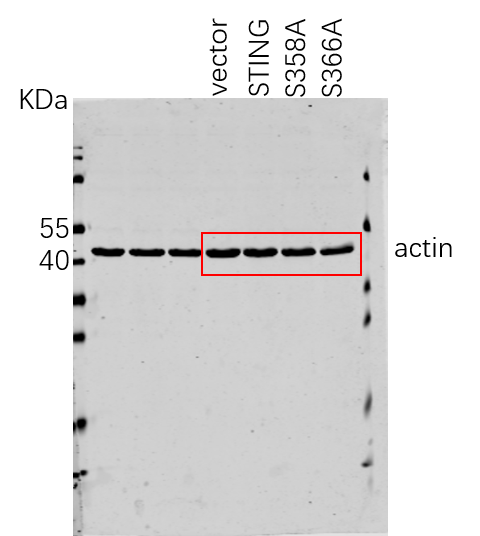

Supplement: Supplementary file 5 — Source data Fig. 3 [file 44319_2025_551_MOESM5_ESM.zip › Fig3/Fig3G/Fig3G IB Actin.png]

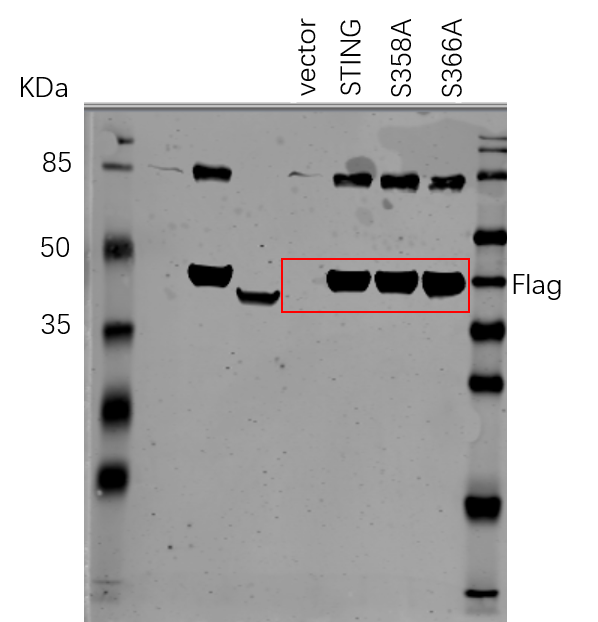

Supplement: Supplementary file 5 — Source data Fig. 3 [file 44319_2025_551_MOESM5_ESM.zip › Fig3/Fig3G/Fig3G IB Flag.png]

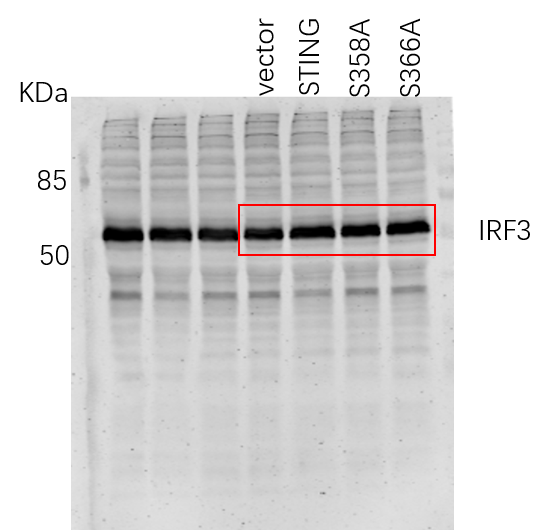

Supplement: Supplementary file 5 — Source data Fig. 3 [file 44319_2025_551_MOESM5_ESM.zip › Fig3/Fig3G/Fig3G IB IRF3.png]

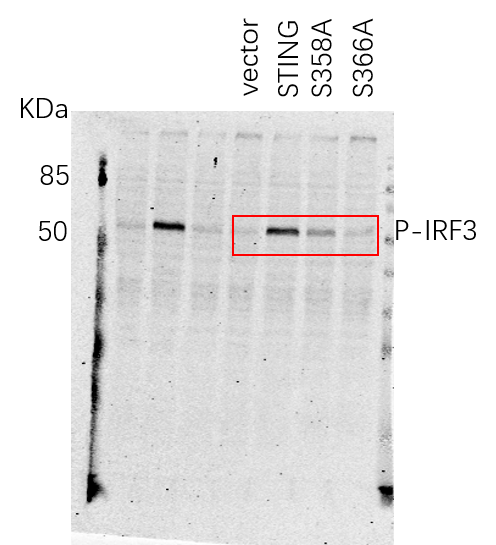

Supplement: Supplementary file 5 — Source data Fig. 3 [file 44319_2025_551_MOESM5_ESM.zip › Fig3/Fig3G/Fig3G IB pIRF3.png]

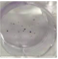

Supplement: Supplementary file 5 — Source data Fig. 3 [file 44319_2025_551_MOESM5_ESM.zip › Fig3/Fig3H/Fig3H image/S358A.tif]

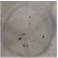

Supplement: Supplementary file 5 — Source data Fig. 3 [file 44319_2025_551_MOESM5_ESM.zip › Fig3/Fig3H/Fig3H image/S366A.tif]

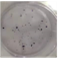

Supplement: Supplementary file 5 — Source data Fig. 3 [file 44319_2025_551_MOESM5_ESM.zip › Fig3/Fig3H/Fig3H image/STING.tif]

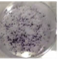

Supplement: Supplementary file 5 — Source data Fig. 3 [file 44319_2025_551_MOESM5_ESM.zip › Fig3/Fig3H/Fig3H image/Vector.tif]

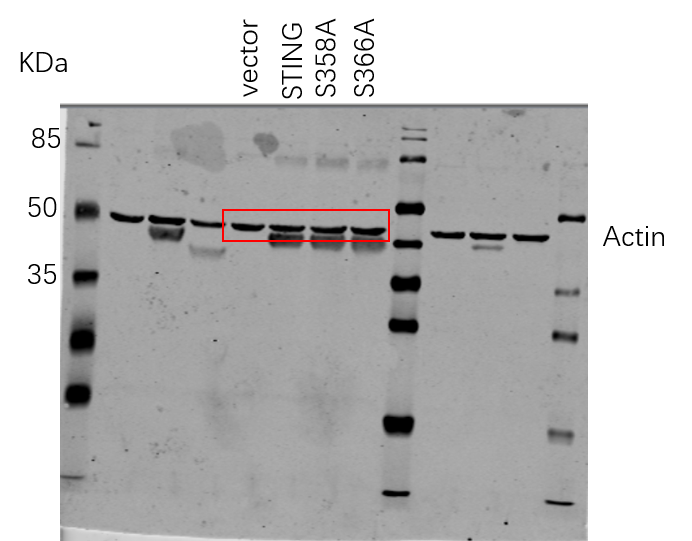

Supplement: Supplementary file 5 — Source data Fig. 3 [file 44319_2025_551_MOESM5_ESM.zip › Fig3/Fig3I/Fig3I IB actin.png]

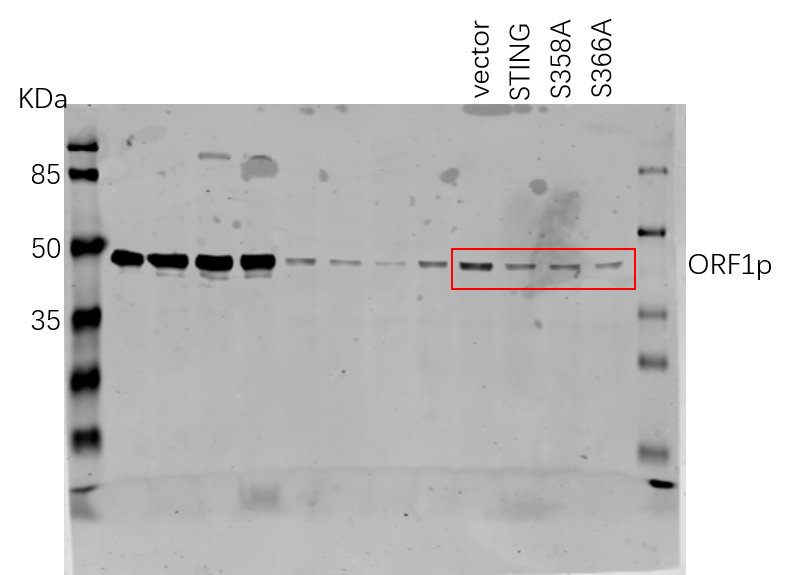

Supplement: Supplementary file 5 — Source data Fig. 3 [file 44319_2025_551_MOESM5_ESM.zip › Fig3/Fig3I/Fig3I IB ORF1p.png]

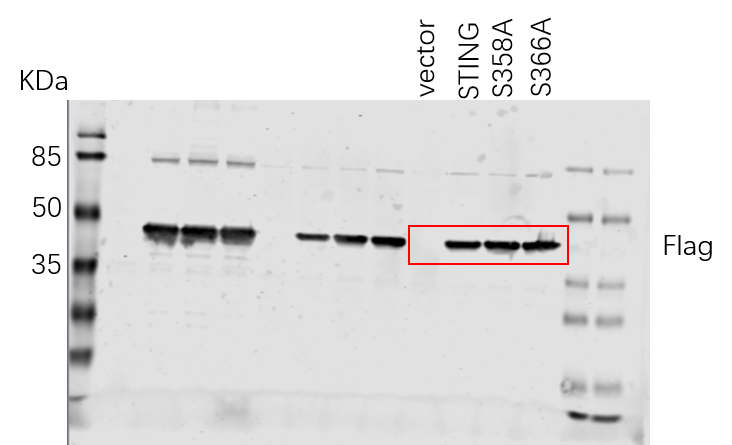

Supplement: Supplementary file 5 — Source data Fig. 3 [file 44319_2025_551_MOESM5_ESM.zip › Fig3/Fig3I/Fig3I IBFlag.png]

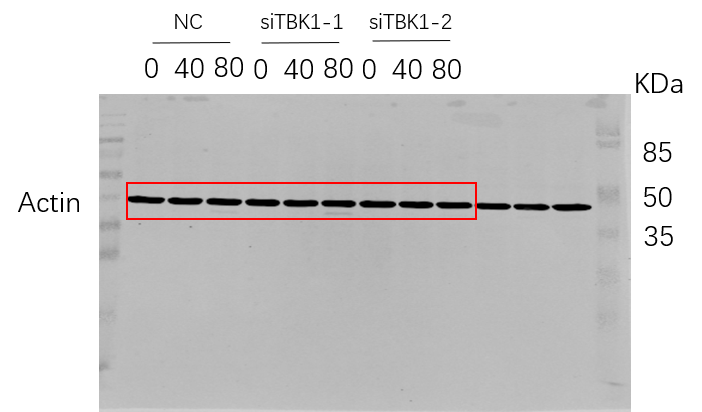

Supplement: Supplementary file 5 — Source data Fig. 3 [file 44319_2025_551_MOESM5_ESM.zip › Fig3/Fig3J/Fig3J IB Actin.png]

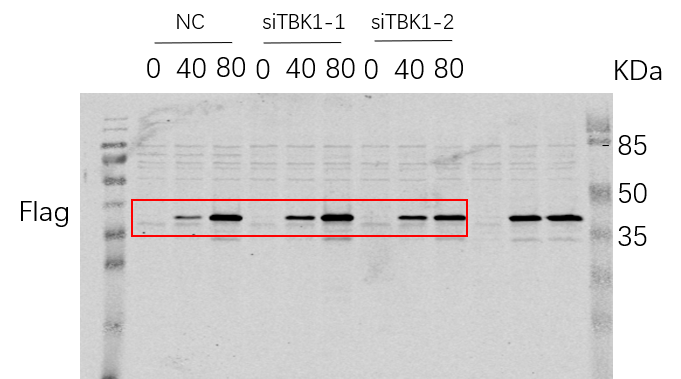

Supplement: Supplementary file 5 — Source data Fig. 3 [file 44319_2025_551_MOESM5_ESM.zip › Fig3/Fig3J/Fig3J IB Flag.png]

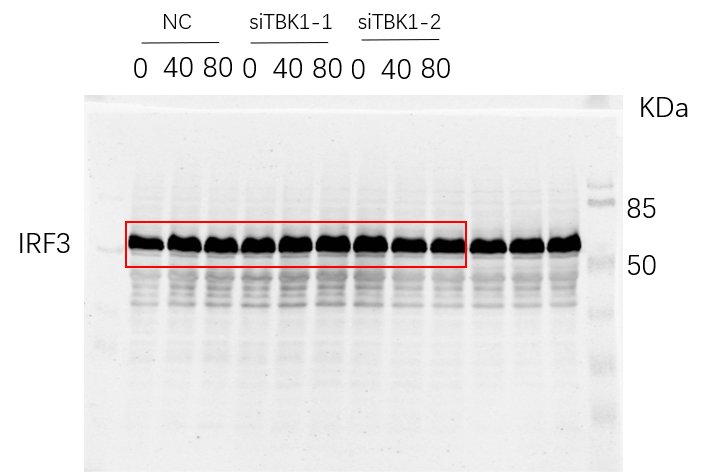

Supplement: Supplementary file 5 — Source data Fig. 3 [file 44319_2025_551_MOESM5_ESM.zip › Fig3/Fig3J/Fig3J IB IRF3.png]

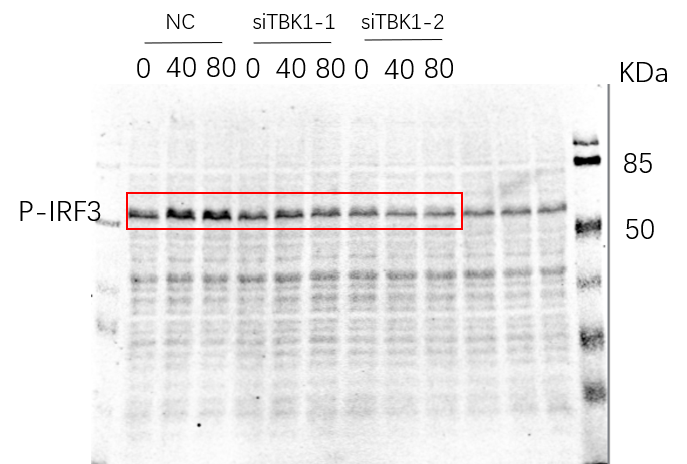

Supplement: Supplementary file 5 — Source data Fig. 3 [file 44319_2025_551_MOESM5_ESM.zip › Fig3/Fig3J/Fig3J IB pIRF3.png]

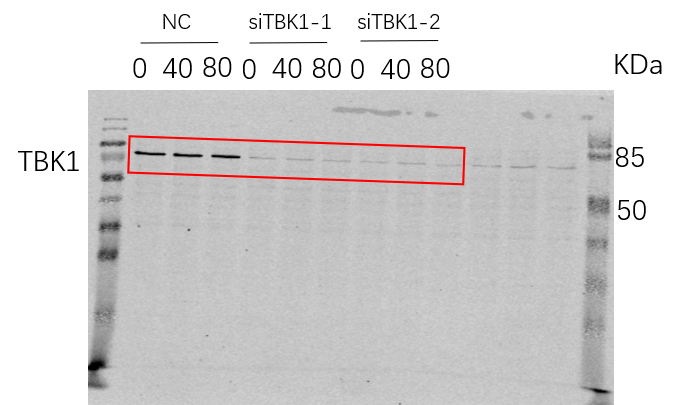

Supplement: Supplementary file 5 — Source data Fig. 3 [file 44319_2025_551_MOESM5_ESM.zip › Fig3/Fig3J/Fig3J IB TBK1.png]

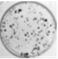

Supplement: Supplementary file 5 — Source data Fig. 3 [file 44319_2025_551_MOESM5_ESM.zip › Fig3/Fig3K/Fig3K image/NC STING 0ng.tif]

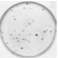

Supplement: Supplementary file 5 — Source data Fig. 3 [file 44319_2025_551_MOESM5_ESM.zip › Fig3/Fig3K/Fig3K image/NC STING 40ng.tif]

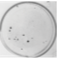

Supplement: Supplementary file 5 — Source data Fig. 3 [file 44319_2025_551_MOESM5_ESM.zip › Fig3/Fig3K/Fig3K image/NC STING 800ng.tif]

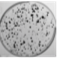

Supplement: Supplementary file 5 — Source data Fig. 3 [file 44319_2025_551_MOESM5_ESM.zip › Fig3/Fig3K/Fig3K image/siTBK1-1 STING 0ng.tif]

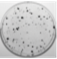

Supplement: Supplementary file 5 — Source data Fig. 3 [file 44319_2025_551_MOESM5_ESM.zip › Fig3/Fig3K/Fig3K image/siTBK1-1 STING 40ng.tif]

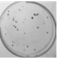

Supplement: Supplementary file 5 — Source data Fig. 3 [file 44319_2025_551_MOESM5_ESM.zip › Fig3/Fig3K/Fig3K image/siTBK1-1 STING 80ng.tif]

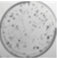

Supplement: Supplementary file 5 — Source data Fig. 3 [file 44319_2025_551_MOESM5_ESM.zip › Fig3/Fig3K/Fig3K image/siTBK1-2 STING 0ng.tif]

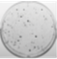

Supplement: Supplementary file 5 — Source data Fig. 3 [file 44319_2025_551_MOESM5_ESM.zip › Fig3/Fig3K/Fig3K image/siTBK1-2 STING 40ng.tif]

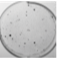

Supplement: Supplementary file 5 — Source data Fig. 3 [file 44319_2025_551_MOESM5_ESM.zip › Fig3/Fig3K/Fig3K image/siTBK1-2 STING 80ng.tif]

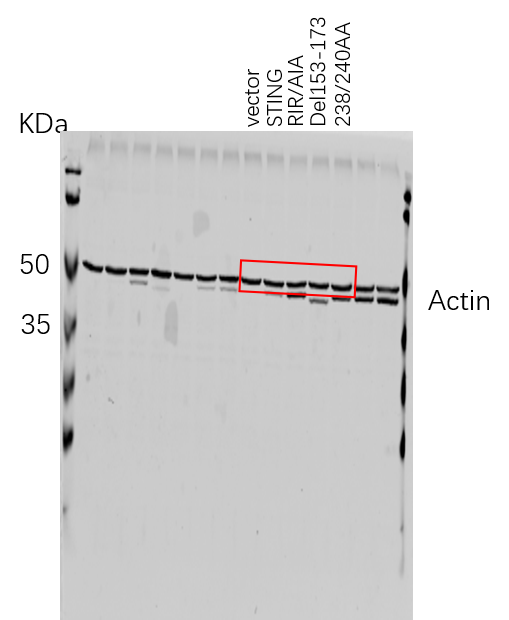

Supplement: Supplementary file 6 — Source data Fig. 4 [file 44319_2025_551_MOESM6_ESM.zip › Fig4/Fig4B/Fig4B IB Actin.png]
