# Supplementary figures and images for: STING inhibits LINE-1 retrotransposition through sorting ORF1p to lysosomes for degradation (part 2 of 4)
Source: EMBO Rep. 2025 Aug 18;26(18):4607–30. doi: 10.1038/s44319-025-00551-0 (PMC12457603; doi:10.1038/s44319-025-00551-0)

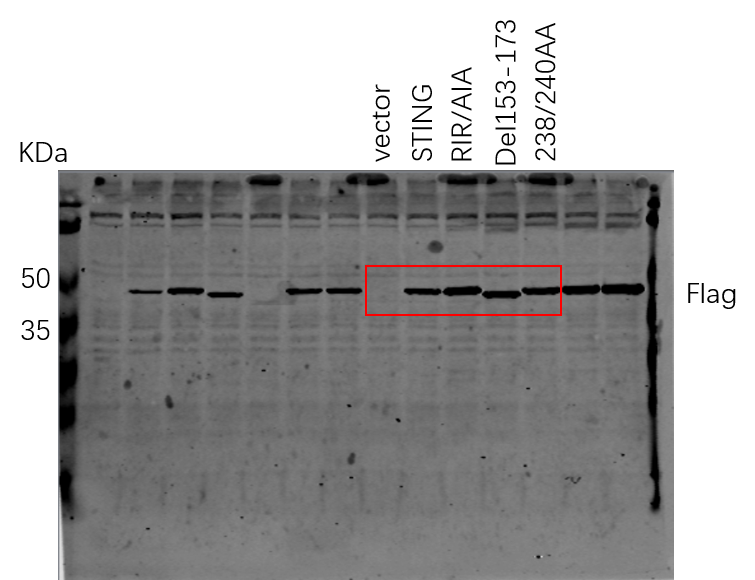

Supplement: Supplementary file 6 — Source data Fig. 4 [file 44319_2025_551_MOESM6_ESM.zip › Fig4/Fig4B/Fig4B IB Flag.png]

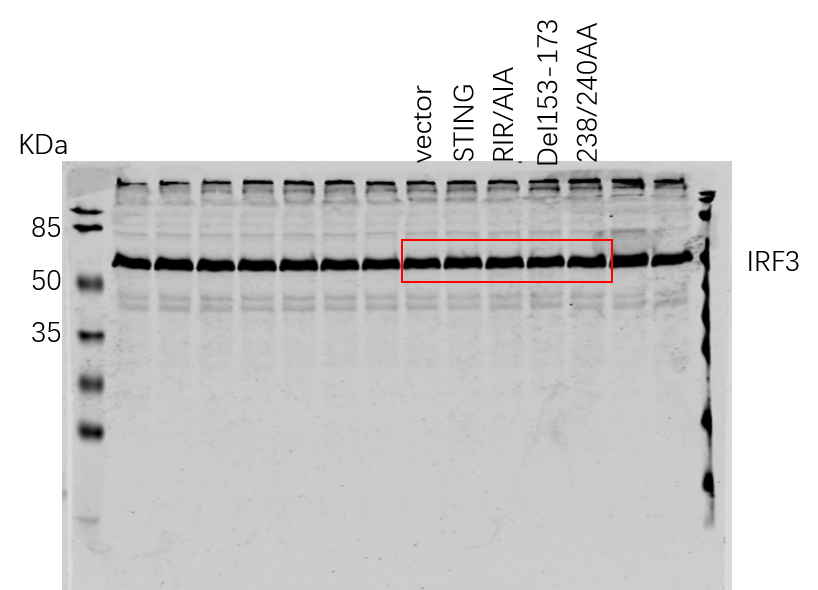

Supplement: Supplementary file 6 — Source data Fig. 4 [file 44319_2025_551_MOESM6_ESM.zip › Fig4/Fig4B/Fig4B IB IRF3.png]

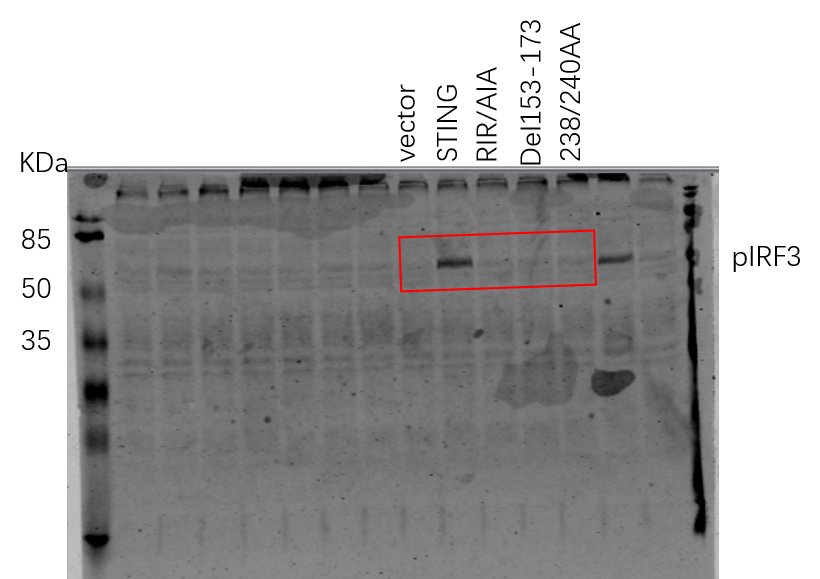

Supplement: Supplementary file 6 — Source data Fig. 4 [file 44319_2025_551_MOESM6_ESM.zip › Fig4/Fig4B/Fig4B IB pIRF3.png]

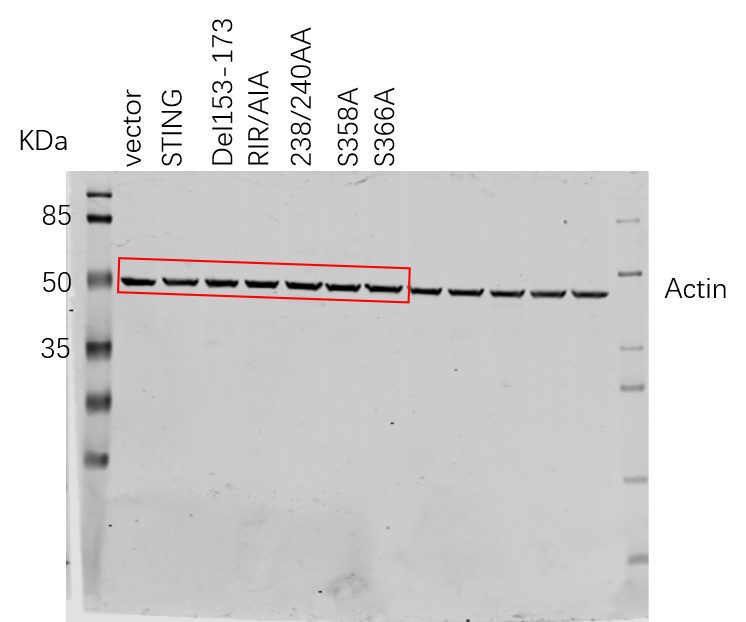

Supplement: Supplementary file 6 — Source data Fig. 4 [file 44319_2025_551_MOESM6_ESM.zip › Fig4/Fig4C/Fig4C Image/Fig4C IB Actin.png]

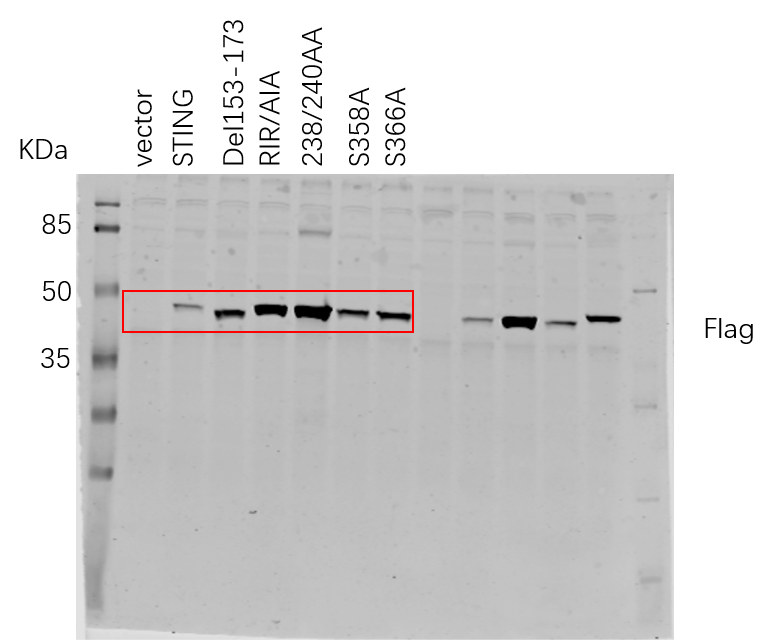

Supplement: Supplementary file 6 — Source data Fig. 4 [file 44319_2025_551_MOESM6_ESM.zip › Fig4/Fig4C/Fig4C Image/Fig4C IB Flag.png]

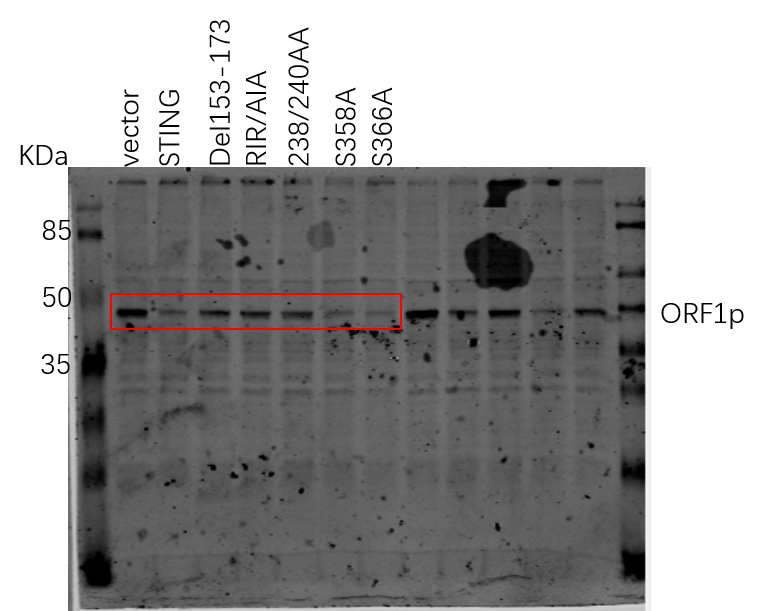

Supplement: Supplementary file 6 — Source data Fig. 4 [file 44319_2025_551_MOESM6_ESM.zip › Fig4/Fig4C/Fig4C Image/Fig4C IB ORF1p.png]

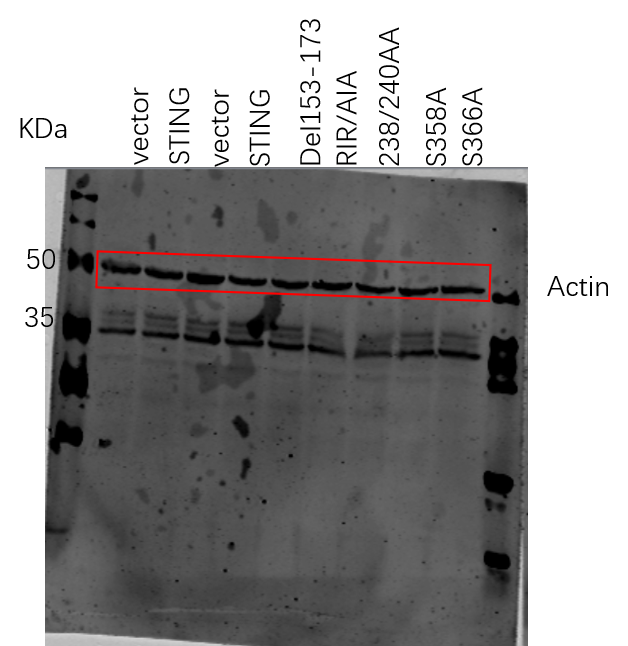

Supplement: Supplementary file 6 — Source data Fig. 4 [file 44319_2025_551_MOESM6_ESM.zip › Fig4/Fig4D/Fig4D IB Actin.png]

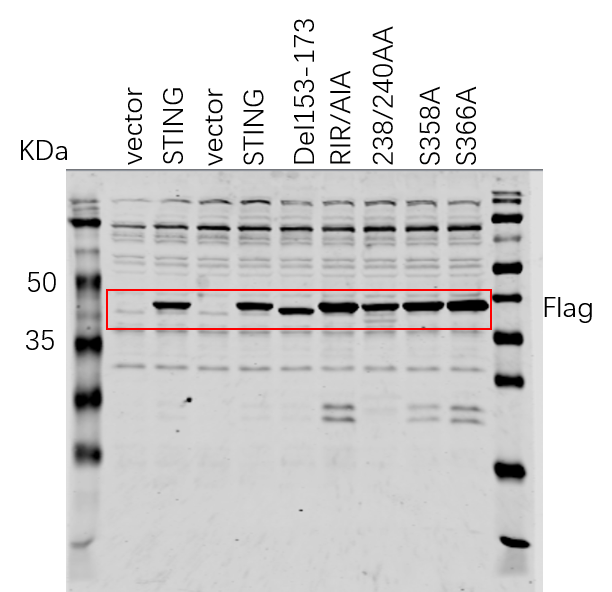

Supplement: Supplementary file 6 — Source data Fig. 4 [file 44319_2025_551_MOESM6_ESM.zip › Fig4/Fig4D/Fig4D IB Flag.png]

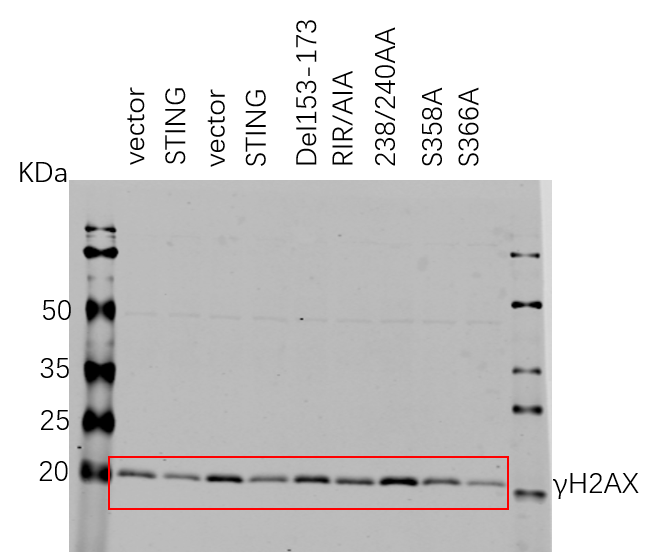

Supplement: Supplementary file 6 — Source data Fig. 4 [file 44319_2025_551_MOESM6_ESM.zip › Fig4/Fig4D/Fig4D IB γH2AX.png]

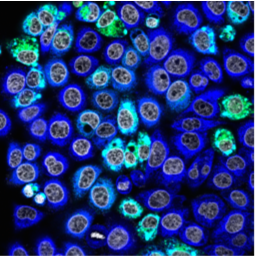

Supplement: Supplementary file 6 — Source data Fig. 4 [file 44319_2025_551_MOESM6_ESM.zip › Fig4/Fig4E/Fig4E ER/STING-EGFP/1X merge.png]

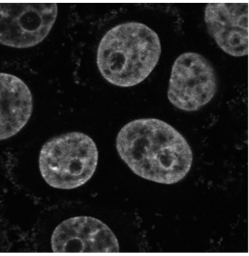

Supplement: Supplementary file 6 — Source data Fig. 4 [file 44319_2025_551_MOESM6_ESM.zip › Fig4/Fig4E/Fig4E ER/STING-EGFP/3X DAPI.png]

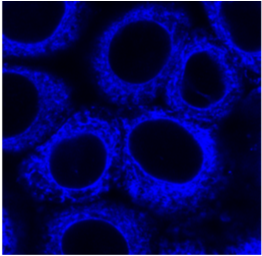

Supplement: Supplementary file 6 — Source data Fig. 4 [file 44319_2025_551_MOESM6_ESM.zip › Fig4/Fig4E/Fig4E ER/STING-EGFP/3X ER.png]

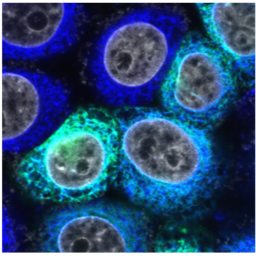

Supplement: Supplementary file 6 — Source data Fig. 4 [file 44319_2025_551_MOESM6_ESM.zip › Fig4/Fig4E/Fig4E ER/STING-EGFP/3X merge.png]

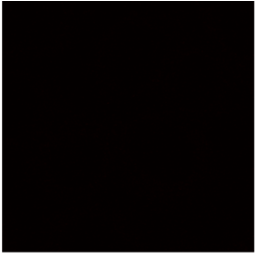

Supplement: Supplementary file 6 — Source data Fig. 4 [file 44319_2025_551_MOESM6_ESM.zip › Fig4/Fig4E/Fig4E ER/STING-EGFP/3X ORF1p.png]

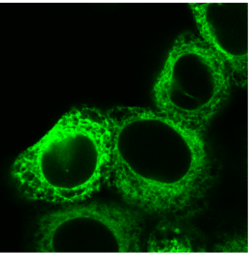

Supplement: Supplementary file 6 — Source data Fig. 4 [file 44319_2025_551_MOESM6_ESM.zip › Fig4/Fig4E/Fig4E ER/STING-EGFP/3X STING.png]

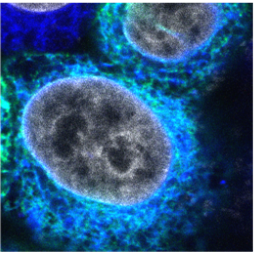

Supplement: Supplementary file 6 — Source data Fig. 4 [file 44319_2025_551_MOESM6_ESM.zip › Fig4/Fig4E/Fig4E ER/STING-EGFP/6X merge.png]

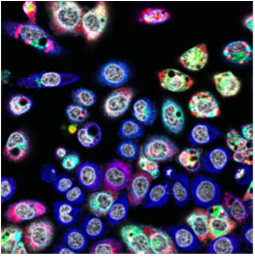

Supplement: Supplementary file 6 — Source data Fig. 4 [file 44319_2025_551_MOESM6_ESM.zip › Fig4/Fig4E/Fig4E ER/STING-EGFP+L1/1X merge.png]

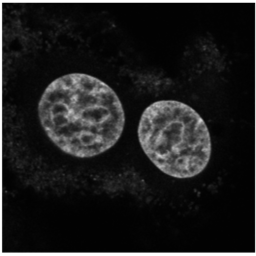

Supplement: Supplementary file 6 — Source data Fig. 4 [file 44319_2025_551_MOESM6_ESM.zip › Fig4/Fig4E/Fig4E ER/STING-EGFP+L1/3X DAPI.png]

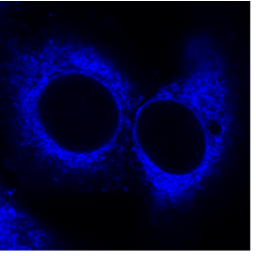

Supplement: Supplementary file 6 — Source data Fig. 4 [file 44319_2025_551_MOESM6_ESM.zip › Fig4/Fig4E/Fig4E ER/STING-EGFP+L1/3X ER.png]

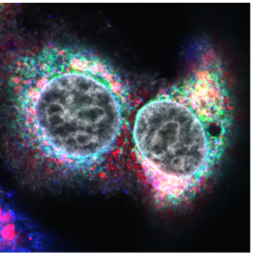

Supplement: Supplementary file 6 — Source data Fig. 4 [file 44319_2025_551_MOESM6_ESM.zip › Fig4/Fig4E/Fig4E ER/STING-EGFP+L1/3X merge.png]

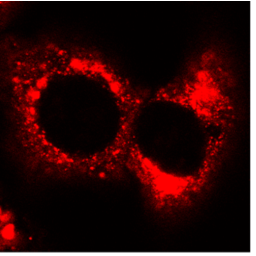

Supplement: Supplementary file 6 — Source data Fig. 4 [file 44319_2025_551_MOESM6_ESM.zip › Fig4/Fig4E/Fig4E ER/STING-EGFP+L1/3X ORF1p.png]

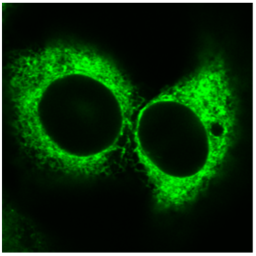

Supplement: Supplementary file 6 — Source data Fig. 4 [file 44319_2025_551_MOESM6_ESM.zip › Fig4/Fig4E/Fig4E ER/STING-EGFP+L1/3X STING.png]

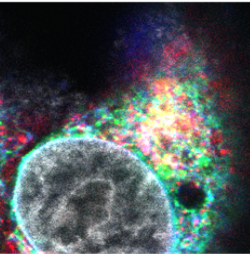

Supplement: Supplementary file 6 — Source data Fig. 4 [file 44319_2025_551_MOESM6_ESM.zip › Fig4/Fig4E/Fig4E ER/STING-EGFP+L1/6X merge.png]

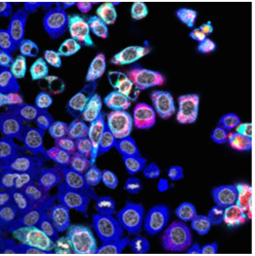

Supplement: Supplementary file 6 — Source data Fig. 4 [file 44319_2025_551_MOESM6_ESM.zip › Fig4/Fig4E/Fig4E ER/STING-EGFP+L1+BFA/1X merge.png]

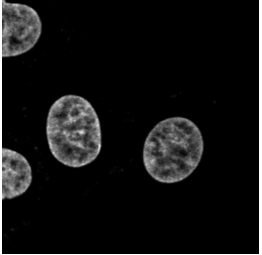

Supplement: Supplementary file 6 — Source data Fig. 4 [file 44319_2025_551_MOESM6_ESM.zip › Fig4/Fig4E/Fig4E ER/STING-EGFP+L1+BFA/3X DAPI.png]

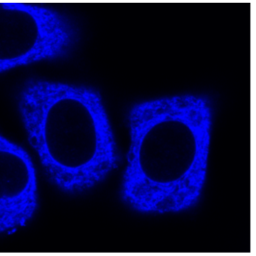

Supplement: Supplementary file 6 — Source data Fig. 4 [file 44319_2025_551_MOESM6_ESM.zip › Fig4/Fig4E/Fig4E ER/STING-EGFP+L1+BFA/3X ER.png]

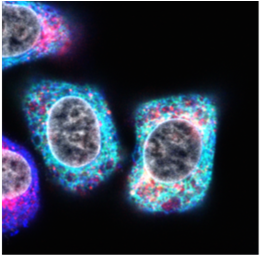

Supplement: Supplementary file 6 — Source data Fig. 4 [file 44319_2025_551_MOESM6_ESM.zip › Fig4/Fig4E/Fig4E ER/STING-EGFP+L1+BFA/3X merge.png]

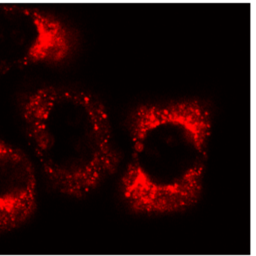

Supplement: Supplementary file 6 — Source data Fig. 4 [file 44319_2025_551_MOESM6_ESM.zip › Fig4/Fig4E/Fig4E ER/STING-EGFP+L1+BFA/3X ORF1p.png]

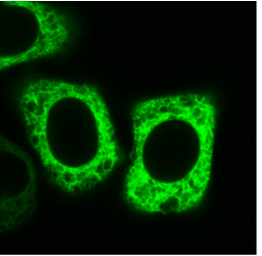

Supplement: Supplementary file 6 — Source data Fig. 4 [file 44319_2025_551_MOESM6_ESM.zip › Fig4/Fig4E/Fig4E ER/STING-EGFP+L1+BFA/3X STING.png]

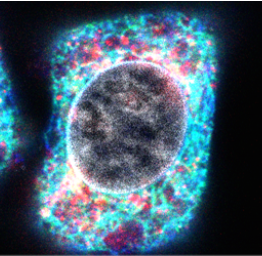

Supplement: Supplementary file 6 — Source data Fig. 4 [file 44319_2025_551_MOESM6_ESM.zip › Fig4/Fig4E/Fig4E ER/STING-EGFP+L1+BFA/6X merge.png]

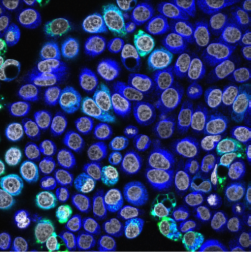

Supplement: Supplementary file 6 — Source data Fig. 4 [file 44319_2025_551_MOESM6_ESM.zip › Fig4/Fig4E/Fig4E ERGIC/STING-EGFP/1X merge.png]

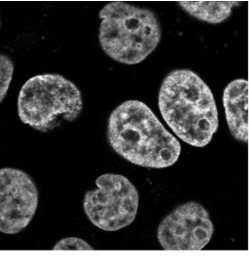

Supplement: Supplementary file 6 — Source data Fig. 4 [file 44319_2025_551_MOESM6_ESM.zip › Fig4/Fig4E/Fig4E ERGIC/STING-EGFP/3X DAPI.png]

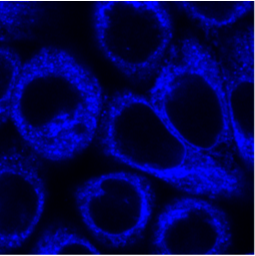

Supplement: Supplementary file 6 — Source data Fig. 4 [file 44319_2025_551_MOESM6_ESM.zip › Fig4/Fig4E/Fig4E ERGIC/STING-EGFP/3X ERGIC.png]

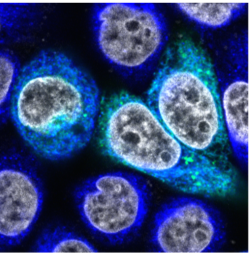

Supplement: Supplementary file 6 — Source data Fig. 4 [file 44319_2025_551_MOESM6_ESM.zip › Fig4/Fig4E/Fig4E ERGIC/STING-EGFP/3X merge.png]

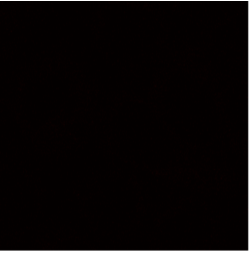

Supplement: Supplementary file 6 — Source data Fig. 4 [file 44319_2025_551_MOESM6_ESM.zip › Fig4/Fig4E/Fig4E ERGIC/STING-EGFP/3X ORF1p.png]

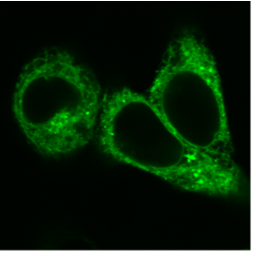

Supplement: Supplementary file 6 — Source data Fig. 4 [file 44319_2025_551_MOESM6_ESM.zip › Fig4/Fig4E/Fig4E ERGIC/STING-EGFP/3X STING.png]

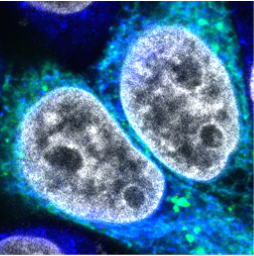

Supplement: Supplementary file 6 — Source data Fig. 4 [file 44319_2025_551_MOESM6_ESM.zip › Fig4/Fig4E/Fig4E ERGIC/STING-EGFP/6X merge.png]

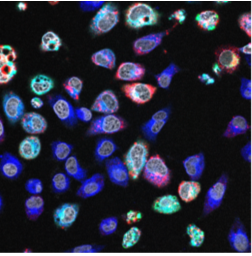

Supplement: Supplementary file 6 — Source data Fig. 4 [file 44319_2025_551_MOESM6_ESM.zip › Fig4/Fig4E/Fig4E ERGIC/STING-EGFP+L1/1X merge.png]

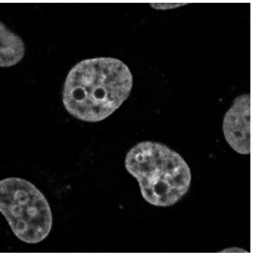

Supplement: Supplementary file 6 — Source data Fig. 4 [file 44319_2025_551_MOESM6_ESM.zip › Fig4/Fig4E/Fig4E ERGIC/STING-EGFP+L1/3X DAPI.png]

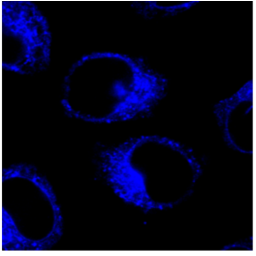

Supplement: Supplementary file 6 — Source data Fig. 4 [file 44319_2025_551_MOESM6_ESM.zip › Fig4/Fig4E/Fig4E ERGIC/STING-EGFP+L1/3X ERGIC.png]

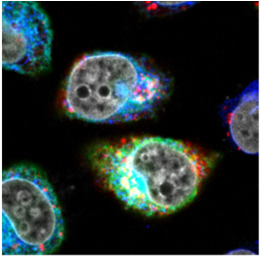

Supplement: Supplementary file 6 — Source data Fig. 4 [file 44319_2025_551_MOESM6_ESM.zip › Fig4/Fig4E/Fig4E ERGIC/STING-EGFP+L1/3X merge.png]

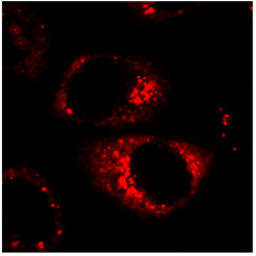

Supplement: Supplementary file 6 — Source data Fig. 4 [file 44319_2025_551_MOESM6_ESM.zip › Fig4/Fig4E/Fig4E ERGIC/STING-EGFP+L1/3X ORF1p.png]

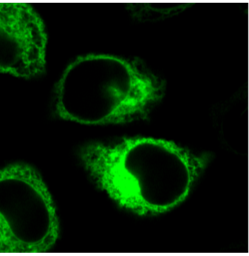

Supplement: Supplementary file 6 — Source data Fig. 4 [file 44319_2025_551_MOESM6_ESM.zip › Fig4/Fig4E/Fig4E ERGIC/STING-EGFP+L1/3X STING.png]

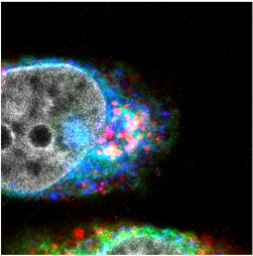

Supplement: Supplementary file 6 — Source data Fig. 4 [file 44319_2025_551_MOESM6_ESM.zip › Fig4/Fig4E/Fig4E ERGIC/STING-EGFP+L1/6X merge.png]

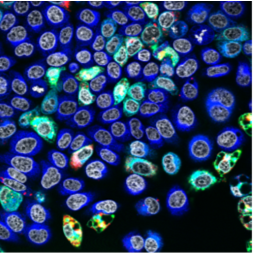

Supplement: Supplementary file 6 — Source data Fig. 4 [file 44319_2025_551_MOESM6_ESM.zip › Fig4/Fig4E/Fig4E ERGIC/STING-EGFP+L1+BFA/1X merge.png]

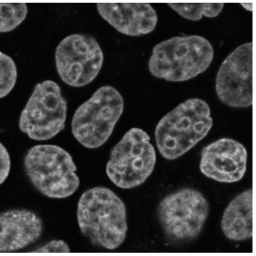

Supplement: Supplementary file 6 — Source data Fig. 4 [file 44319_2025_551_MOESM6_ESM.zip › Fig4/Fig4E/Fig4E ERGIC/STING-EGFP+L1+BFA/3X DAPI.png]

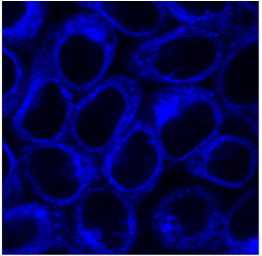

Supplement: Supplementary file 6 — Source data Fig. 4 [file 44319_2025_551_MOESM6_ESM.zip › Fig4/Fig4E/Fig4E ERGIC/STING-EGFP+L1+BFA/3X ERGIC.png]

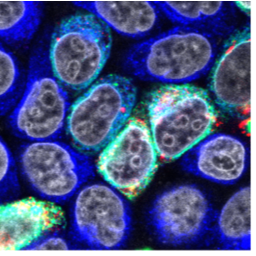

Supplement: Supplementary file 6 — Source data Fig. 4 [file 44319_2025_551_MOESM6_ESM.zip › Fig4/Fig4E/Fig4E ERGIC/STING-EGFP+L1+BFA/3X merge.png]

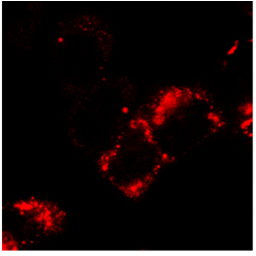

Supplement: Supplementary file 6 — Source data Fig. 4 [file 44319_2025_551_MOESM6_ESM.zip › Fig4/Fig4E/Fig4E ERGIC/STING-EGFP+L1+BFA/3X ORF1p.png]

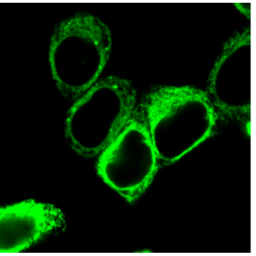

Supplement: Supplementary file 6 — Source data Fig. 4 [file 44319_2025_551_MOESM6_ESM.zip › Fig4/Fig4E/Fig4E ERGIC/STING-EGFP+L1+BFA/3X STING.png]

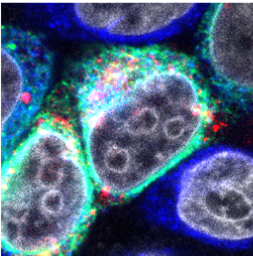

Supplement: Supplementary file 6 — Source data Fig. 4 [file 44319_2025_551_MOESM6_ESM.zip › Fig4/Fig4E/Fig4E ERGIC/STING-EGFP+L1+BFA/6X merge.png]

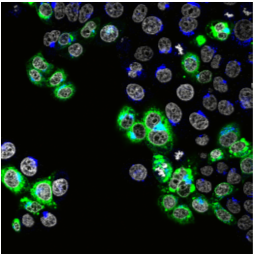

Supplement: Supplementary file 6 — Source data Fig. 4 [file 44319_2025_551_MOESM6_ESM.zip › Fig4/Fig4E/Fig4E Golgi/STING-EGFP/1X merge.png]

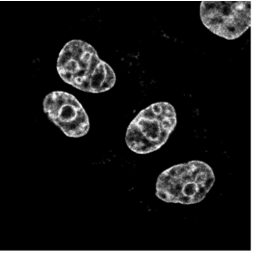

Supplement: Supplementary file 6 — Source data Fig. 4 [file 44319_2025_551_MOESM6_ESM.zip › Fig4/Fig4E/Fig4E Golgi/STING-EGFP/3X DAPI.png]

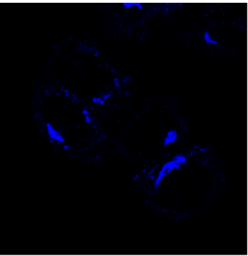

Supplement: Supplementary file 6 — Source data Fig. 4 [file 44319_2025_551_MOESM6_ESM.zip › Fig4/Fig4E/Fig4E Golgi/STING-EGFP/3X Golgi.png]

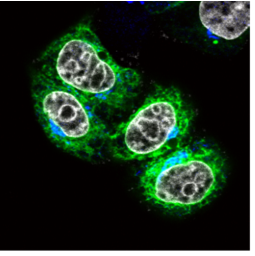

Supplement: Supplementary file 6 — Source data Fig. 4 [file 44319_2025_551_MOESM6_ESM.zip › Fig4/Fig4E/Fig4E Golgi/STING-EGFP/3X Merge.png]

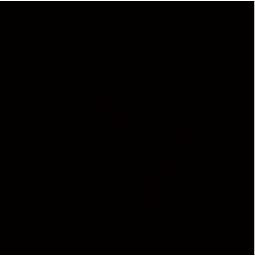

Supplement: Supplementary file 6 — Source data Fig. 4 [file 44319_2025_551_MOESM6_ESM.zip › Fig4/Fig4E/Fig4E Golgi/STING-EGFP/3X ORF1p.png]

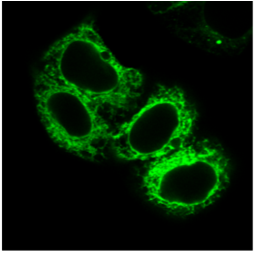

Supplement: Supplementary file 6 — Source data Fig. 4 [file 44319_2025_551_MOESM6_ESM.zip › Fig4/Fig4E/Fig4E Golgi/STING-EGFP/3X STING.png]

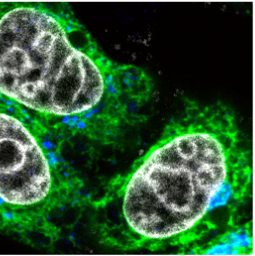

Supplement: Supplementary file 6 — Source data Fig. 4 [file 44319_2025_551_MOESM6_ESM.zip › Fig4/Fig4E/Fig4E Golgi/STING-EGFP/6X merge.png]

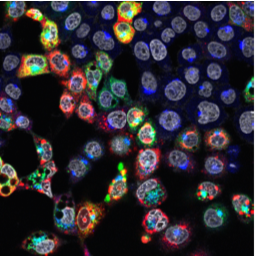

Supplement: Supplementary file 6 — Source data Fig. 4 [file 44319_2025_551_MOESM6_ESM.zip › Fig4/Fig4E/Fig4E Golgi/STING-EGFP+L1/1X merge.png]

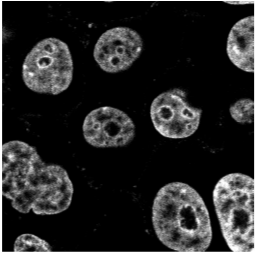

Supplement: Supplementary file 6 — Source data Fig. 4 [file 44319_2025_551_MOESM6_ESM.zip › Fig4/Fig4E/Fig4E Golgi/STING-EGFP+L1/3X DAPI.png]

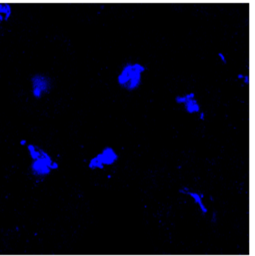

Supplement: Supplementary file 6 — Source data Fig. 4 [file 44319_2025_551_MOESM6_ESM.zip › Fig4/Fig4E/Fig4E Golgi/STING-EGFP+L1/3X Golgi.png]

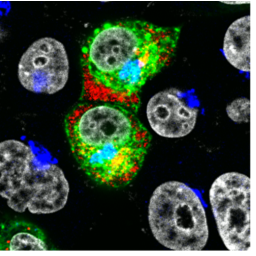

Supplement: Supplementary file 6 — Source data Fig. 4 [file 44319_2025_551_MOESM6_ESM.zip › Fig4/Fig4E/Fig4E Golgi/STING-EGFP+L1/3X merge.png]

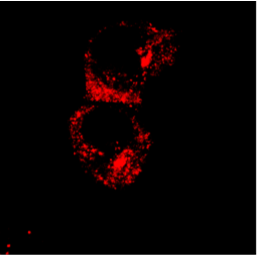

Supplement: Supplementary file 6 — Source data Fig. 4 [file 44319_2025_551_MOESM6_ESM.zip › Fig4/Fig4E/Fig4E Golgi/STING-EGFP+L1/3X ORF1p.png]

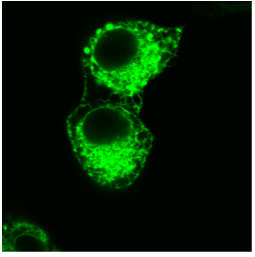

Supplement: Supplementary file 6 — Source data Fig. 4 [file 44319_2025_551_MOESM6_ESM.zip › Fig4/Fig4E/Fig4E Golgi/STING-EGFP+L1/3X STING.png]

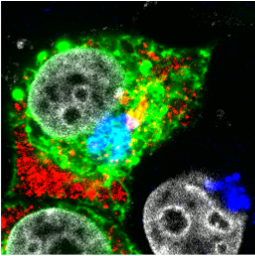

Supplement: Supplementary file 6 — Source data Fig. 4 [file 44319_2025_551_MOESM6_ESM.zip › Fig4/Fig4E/Fig4E Golgi/STING-EGFP+L1/6X merge.png]

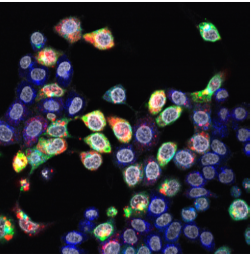

Supplement: Supplementary file 6 — Source data Fig. 4 [file 44319_2025_551_MOESM6_ESM.zip › Fig4/Fig4E/Fig4E Golgi/STING-EGFP+L1+BFA/1X merge.png]

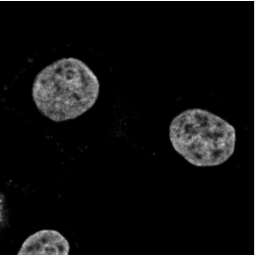

Supplement: Supplementary file 6 — Source data Fig. 4 [file 44319_2025_551_MOESM6_ESM.zip › Fig4/Fig4E/Fig4E Golgi/STING-EGFP+L1+BFA/3X DAPI.png]

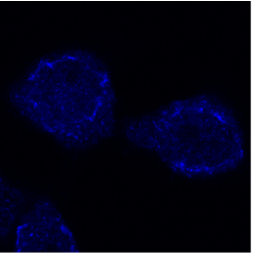

Supplement: Supplementary file 6 — Source data Fig. 4 [file 44319_2025_551_MOESM6_ESM.zip › Fig4/Fig4E/Fig4E Golgi/STING-EGFP+L1+BFA/3X Golgi.png]

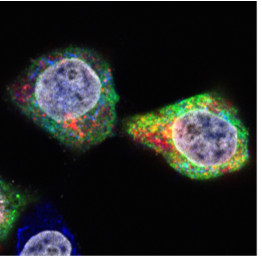

Supplement: Supplementary file 6 — Source data Fig. 4 [file 44319_2025_551_MOESM6_ESM.zip › Fig4/Fig4E/Fig4E Golgi/STING-EGFP+L1+BFA/3X merge.png]

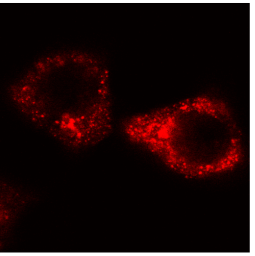

Supplement: Supplementary file 6 — Source data Fig. 4 [file 44319_2025_551_MOESM6_ESM.zip › Fig4/Fig4E/Fig4E Golgi/STING-EGFP+L1+BFA/3X ORF1p.png]

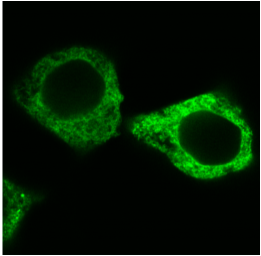

Supplement: Supplementary file 6 — Source data Fig. 4 [file 44319_2025_551_MOESM6_ESM.zip › Fig4/Fig4E/Fig4E Golgi/STING-EGFP+L1+BFA/3X STING.png]

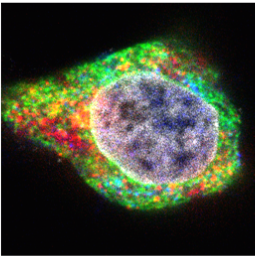

Supplement: Supplementary file 6 — Source data Fig. 4 [file 44319_2025_551_MOESM6_ESM.zip › Fig4/Fig4E/Fig4E Golgi/STING-EGFP+L1+BFA/6X merge.png]

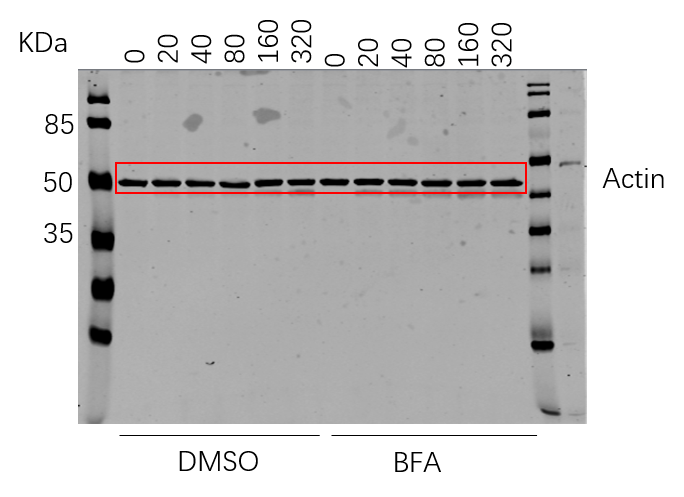

Supplement: Supplementary file 6 — Source data Fig. 4 [file 44319_2025_551_MOESM6_ESM.zip › Fig4/Fig4F/Fig4F IB Actin.png]

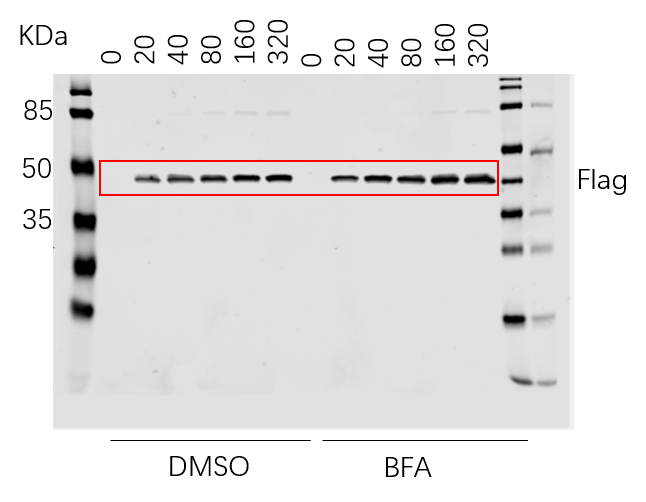

Supplement: Supplementary file 6 — Source data Fig. 4 [file 44319_2025_551_MOESM6_ESM.zip › Fig4/Fig4F/Fig4F IB Flag.png]

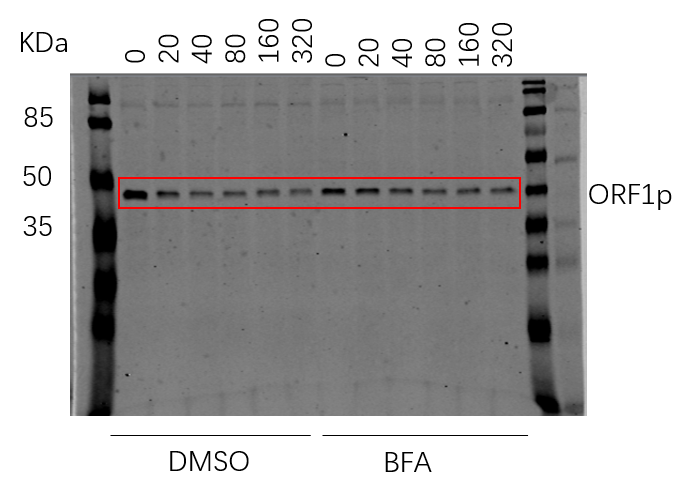

Supplement: Supplementary file 6 — Source data Fig. 4 [file 44319_2025_551_MOESM6_ESM.zip › Fig4/Fig4F/Fig4F IB ORF1p.png]

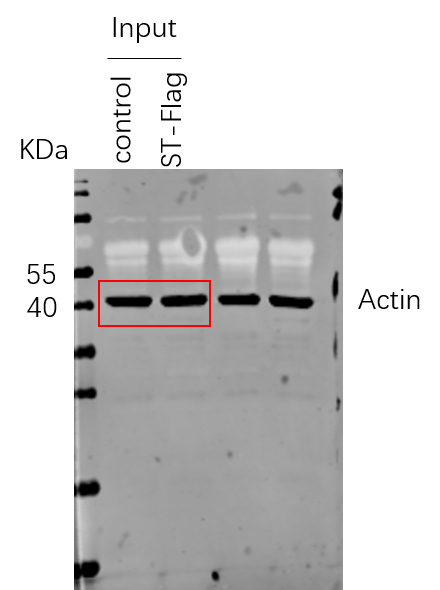

Supplement: Supplementary file 7 — Source data Fig. 5 [file 44319_2025_551_MOESM7_ESM.zip › Fig5/Fig5A/Fig5A endoL1/Fig5A Actin antibody.png]

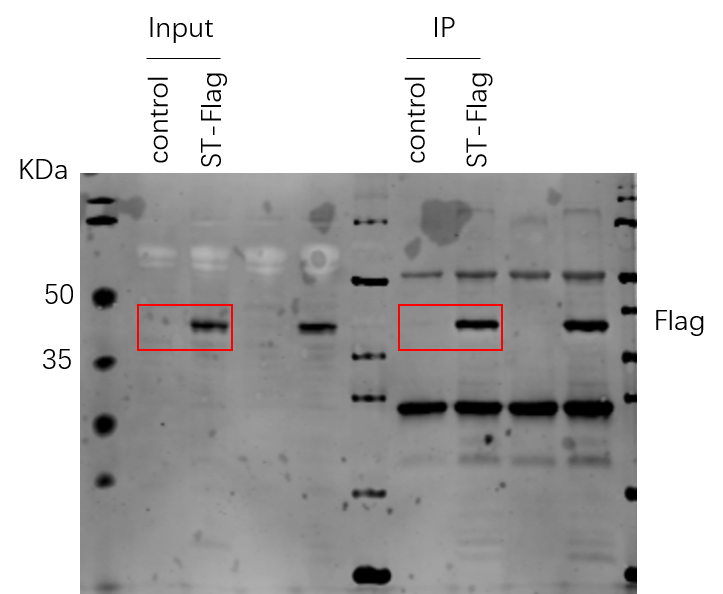

Supplement: Supplementary file 7 — Source data Fig. 5 [file 44319_2025_551_MOESM7_ESM.zip › Fig5/Fig5A/Fig5A endoL1/Fig5A Flag antibody.png]

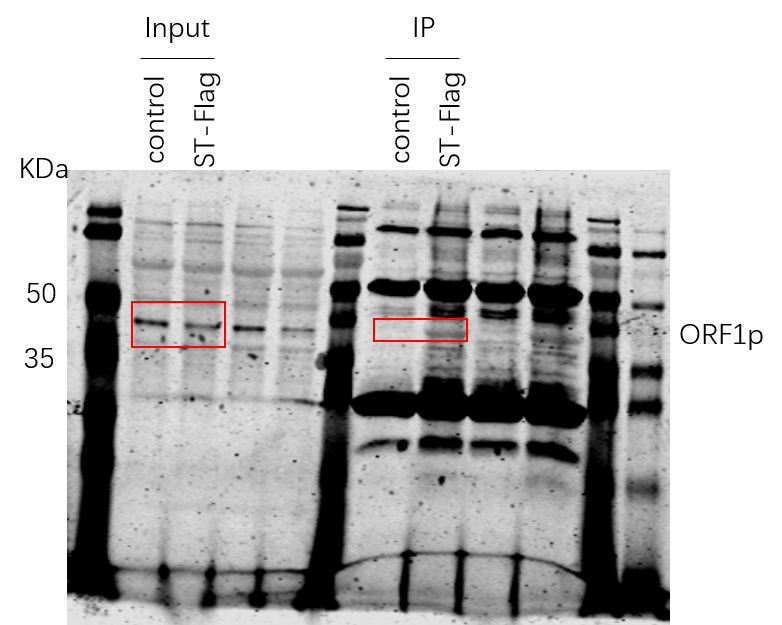

Supplement: Supplementary file 7 — Source data Fig. 5 [file 44319_2025_551_MOESM7_ESM.zip › Fig5/Fig5A/Fig5A endoL1/Fig5A ORF1p antibody.png]

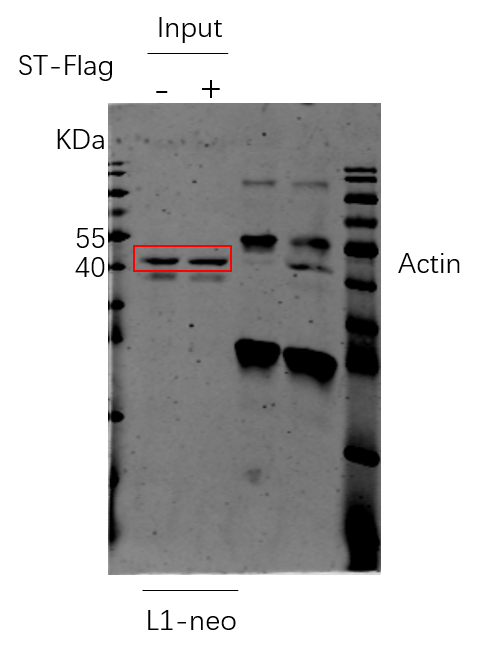

Supplement: Supplementary file 7 — Source data Fig. 5 [file 44319_2025_551_MOESM7_ESM.zip › Fig5/Fig5A/Fig5A L1 neo/Fig5A IB Actin.tif]

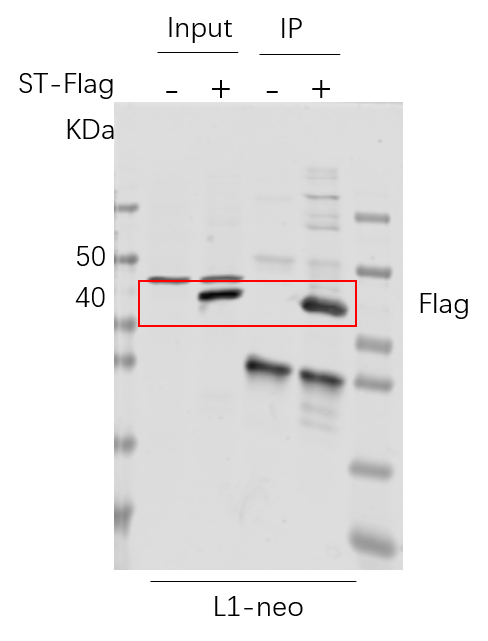

Supplement: Supplementary file 7 — Source data Fig. 5 [file 44319_2025_551_MOESM7_ESM.zip › Fig5/Fig5A/Fig5A L1 neo/Fig5A IB Flag.tif]

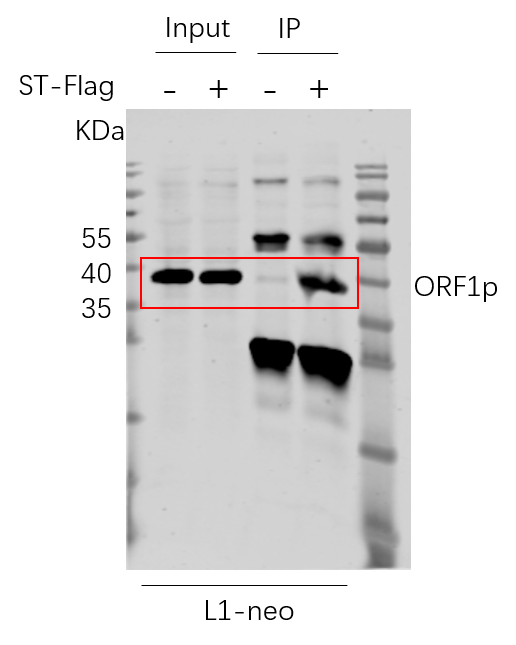

Supplement: Supplementary file 7 — Source data Fig. 5 [file 44319_2025_551_MOESM7_ESM.zip › Fig5/Fig5A/Fig5A L1 neo/Fig5A IB ORF1p.tif]

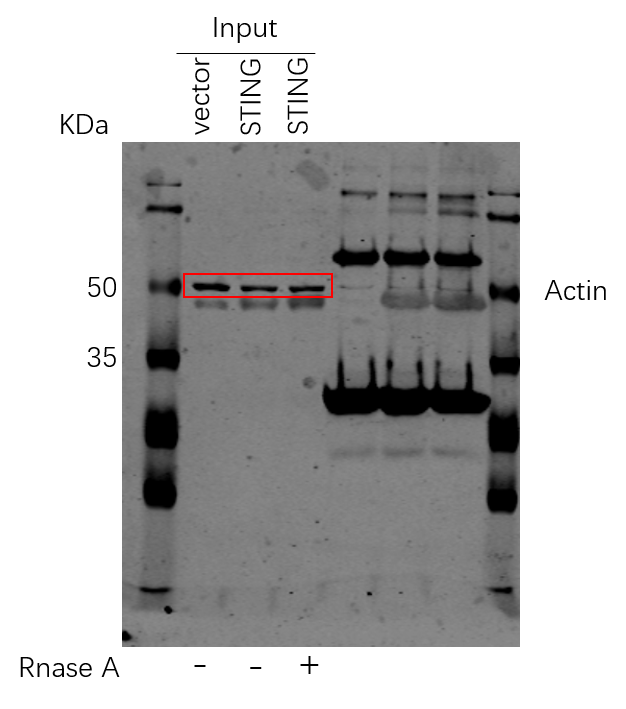

Supplement: Supplementary file 7 — Source data Fig. 5 [file 44319_2025_551_MOESM7_ESM.zip › Fig5/Fig5B/Fig 5B IB Actin.tif]

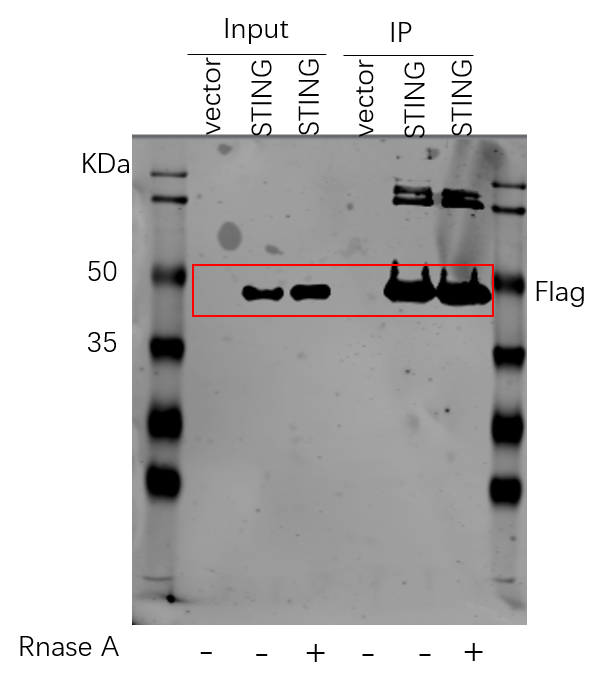

Supplement: Supplementary file 7 — Source data Fig. 5 [file 44319_2025_551_MOESM7_ESM.zip › Fig5/Fig5B/Fig 5B IB Flag.tif]

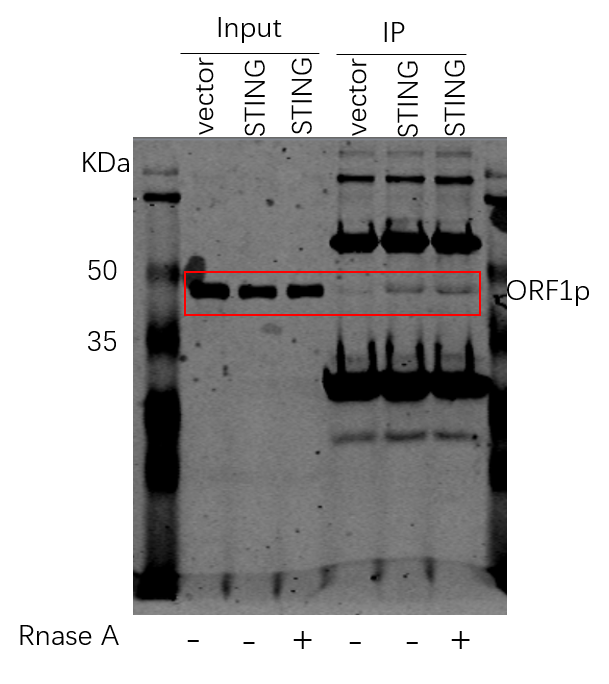

Supplement: Supplementary file 7 — Source data Fig. 5 [file 44319_2025_551_MOESM7_ESM.zip › Fig5/Fig5B/Fig 5B IB ORF1p.tif]

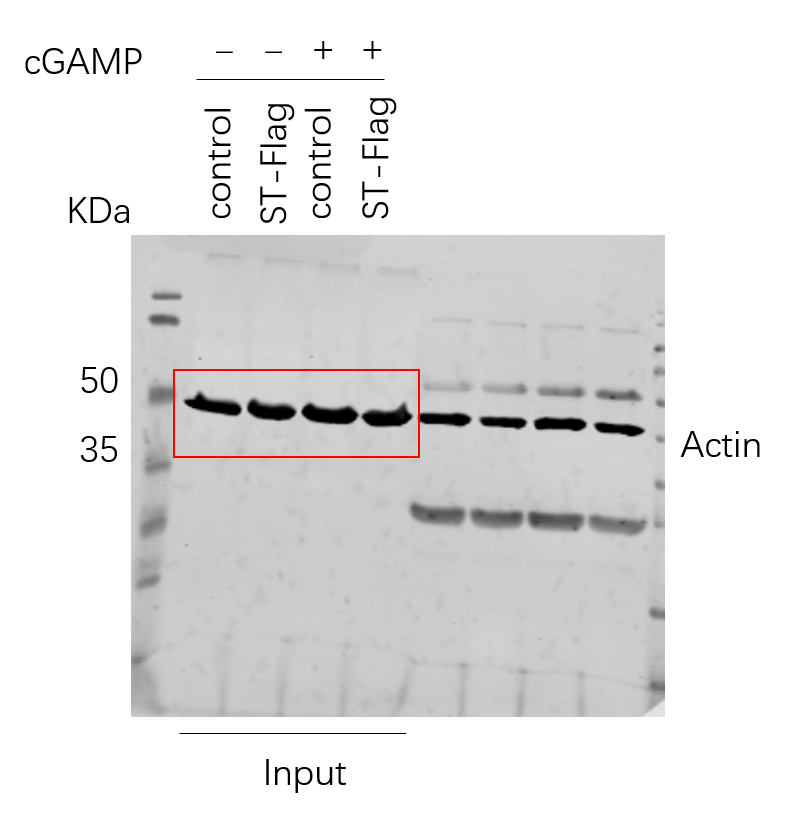

Supplement: Supplementary file 7 — Source data Fig. 5 [file 44319_2025_551_MOESM7_ESM.zip › Fig5/Fig5C/Fig5C Input IB Actin.png]

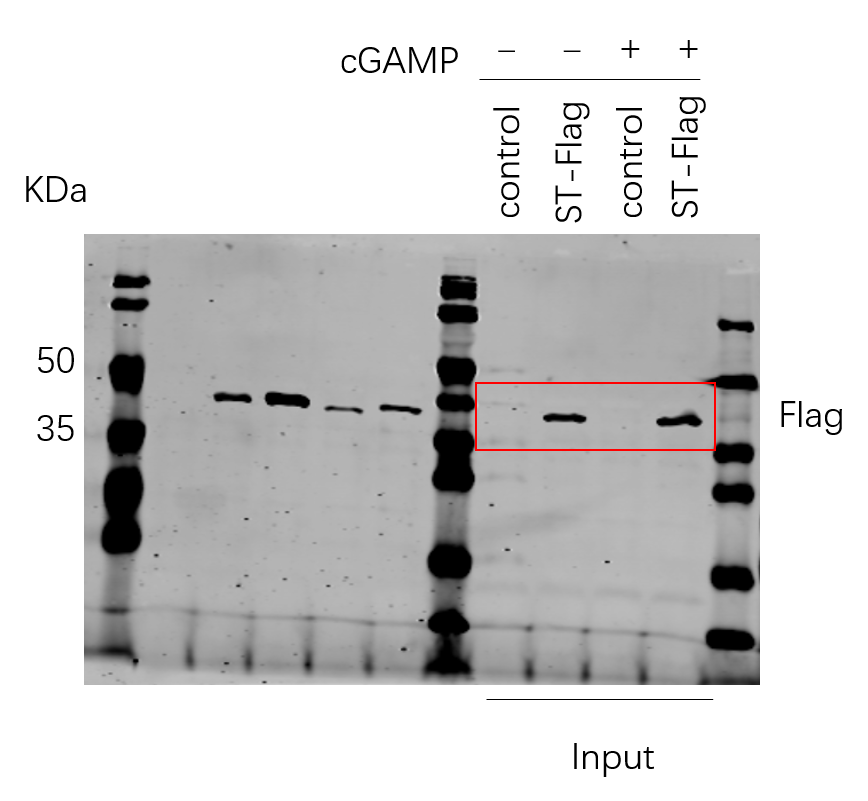

Supplement: Supplementary file 7 — Source data Fig. 5 [file 44319_2025_551_MOESM7_ESM.zip › Fig5/Fig5C/Fig5C Input IB Flag.png]

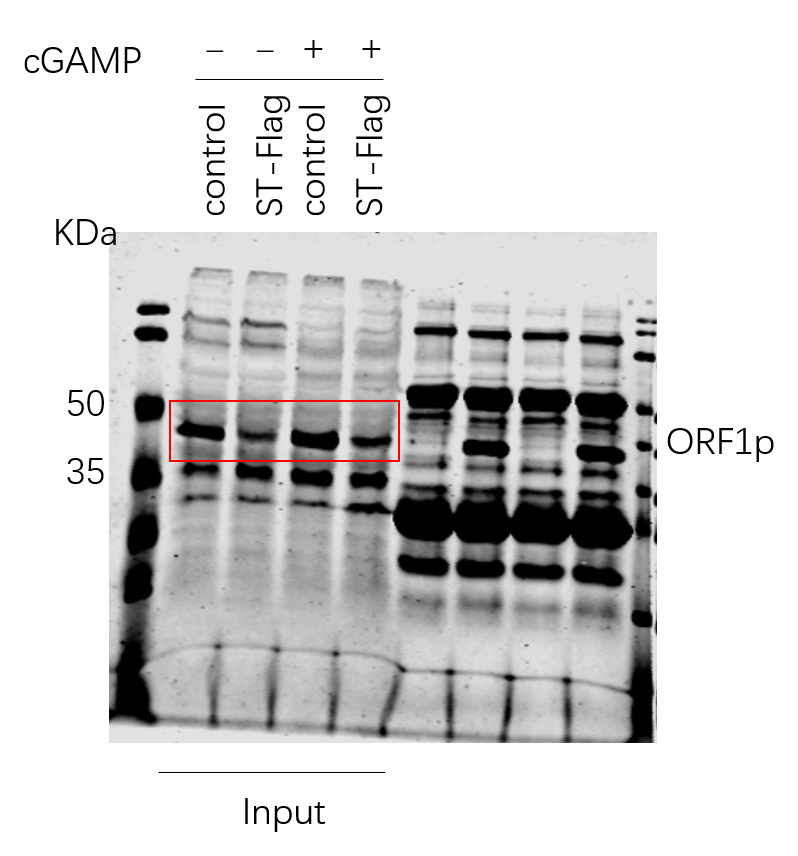

Supplement: Supplementary file 7 — Source data Fig. 5 [file 44319_2025_551_MOESM7_ESM.zip › Fig5/Fig5C/Fig5C Input IB ORF1p.png]

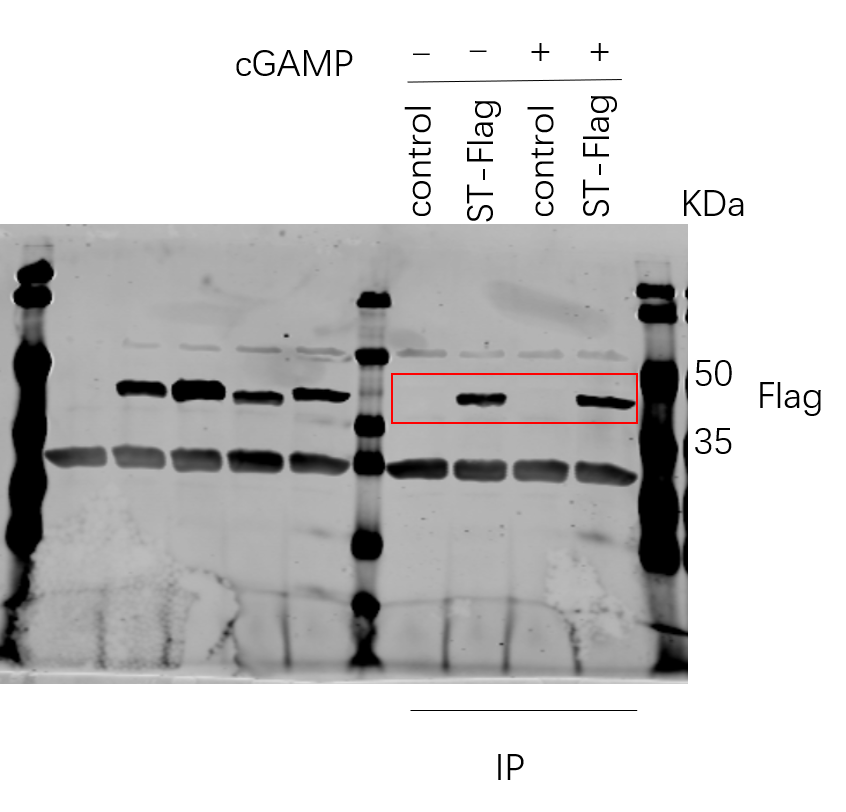

Supplement: Supplementary file 7 — Source data Fig. 5 [file 44319_2025_551_MOESM7_ESM.zip › Fig5/Fig5C/Fig5C IP IB Flag.png]

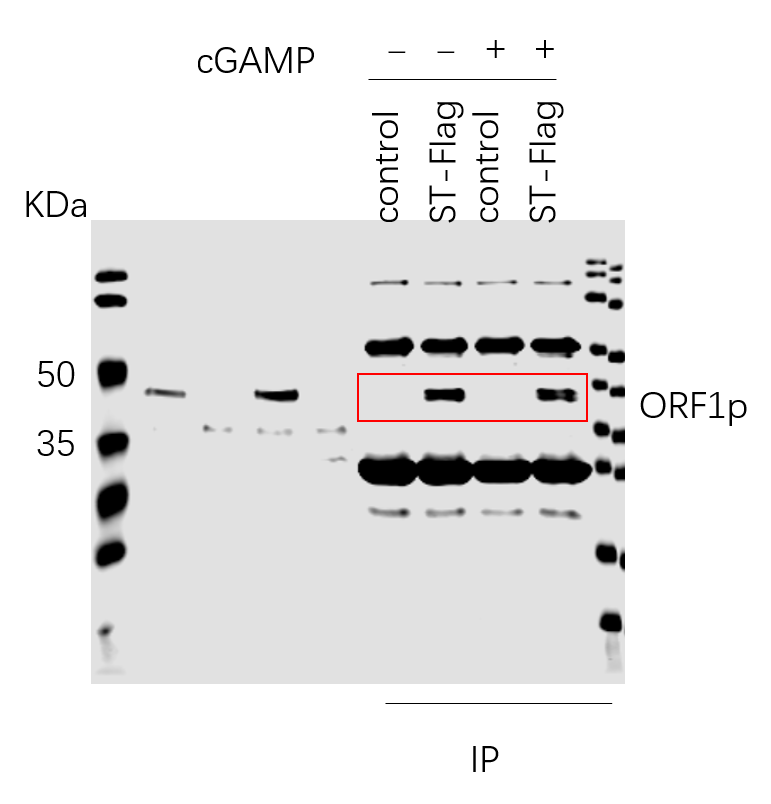

Supplement: Supplementary file 7 — Source data Fig. 5 [file 44319_2025_551_MOESM7_ESM.zip › Fig5/Fig5C/Fig5C IP IB ORF1p.png]

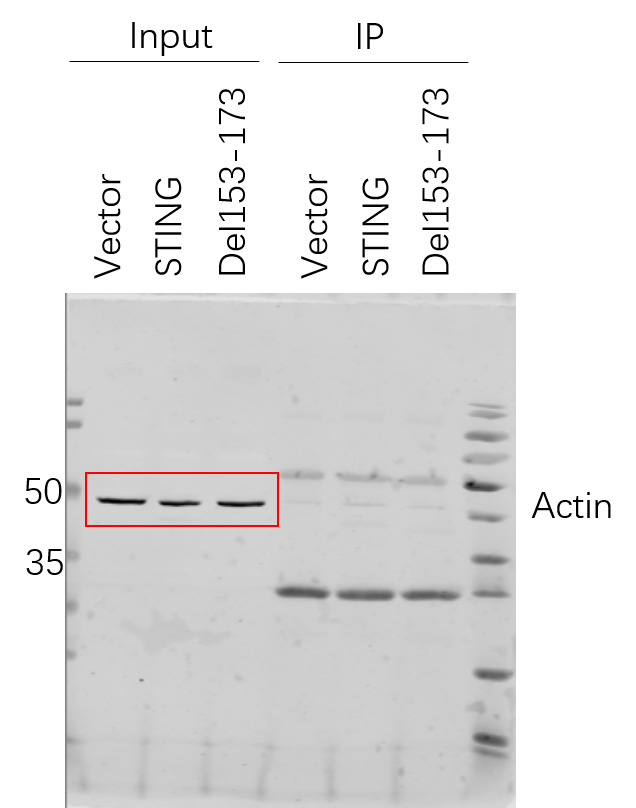

Supplement: Supplementary file 7 — Source data Fig. 5 [file 44319_2025_551_MOESM7_ESM.zip › Fig5/Fig5D/Fig5D IB Actin.png]

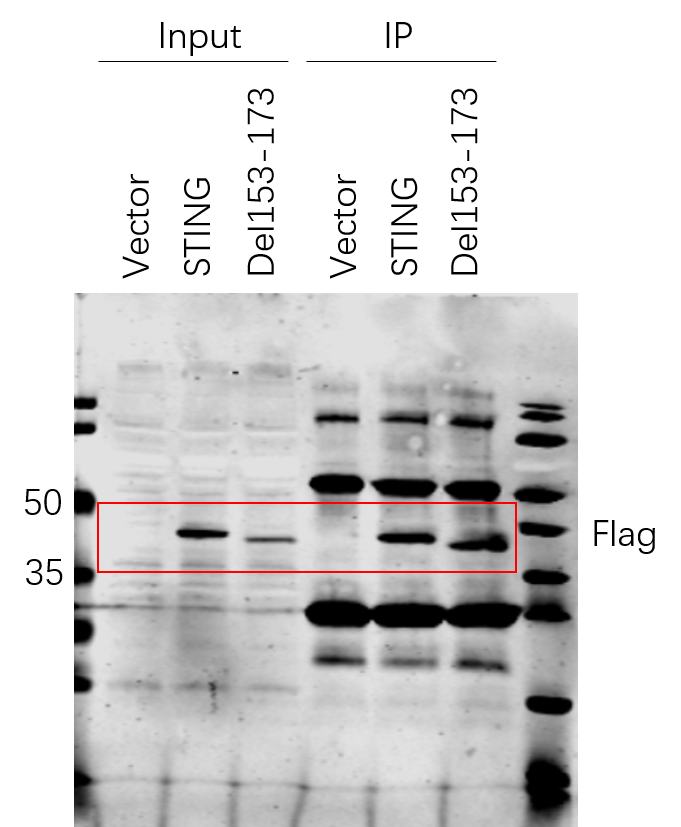

Supplement: Supplementary file 7 — Source data Fig. 5 [file 44319_2025_551_MOESM7_ESM.zip › Fig5/Fig5D/Fig5D IB Flag.png]

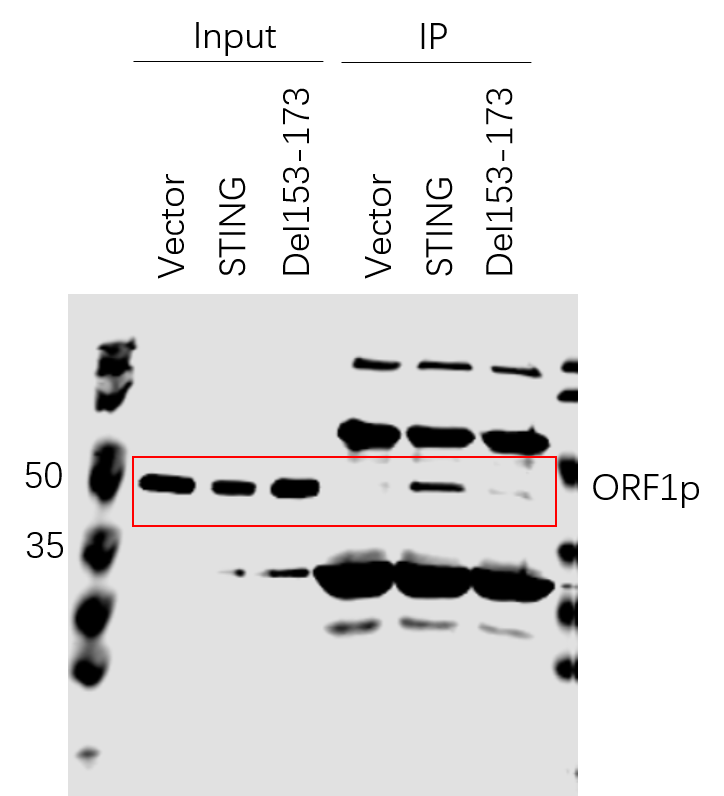

Supplement: Supplementary file 7 — Source data Fig. 5 [file 44319_2025_551_MOESM7_ESM.zip › Fig5/Fig5D/Fig5D IB ORF1p.png]

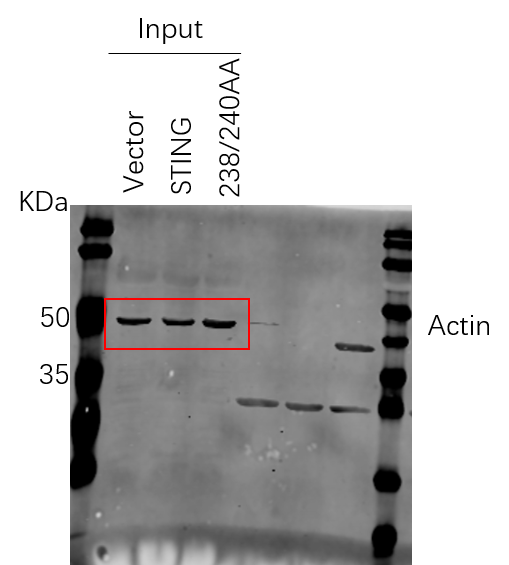

Supplement: Supplementary file 7 — Source data Fig. 5 [file 44319_2025_551_MOESM7_ESM.zip › Fig5/Fig5E/Fig5E IB Actin.png]

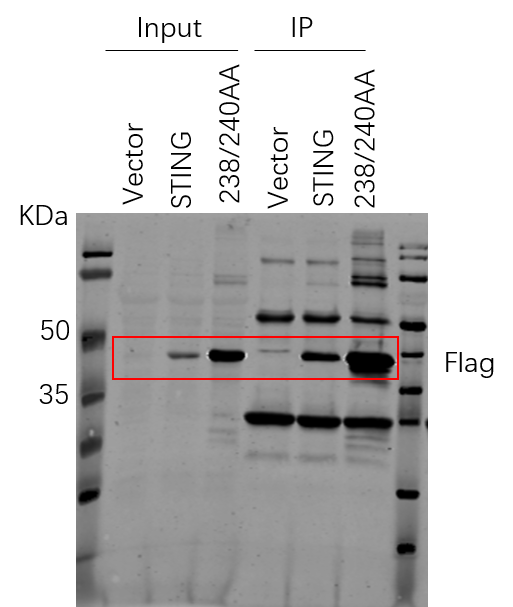

Supplement: Supplementary file 7 — Source data Fig. 5 [file 44319_2025_551_MOESM7_ESM.zip › Fig5/Fig5E/Fig5E IB Flag.png]

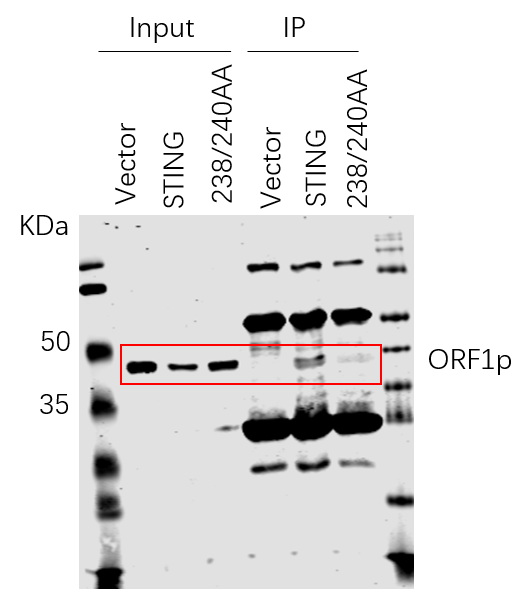

Supplement: Supplementary file 7 — Source data Fig. 5 [file 44319_2025_551_MOESM7_ESM.zip › Fig5/Fig5E/Fig5E IB ORF1p.png]

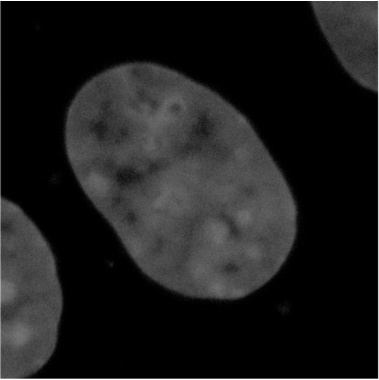

Supplement: Supplementary file 7 — Source data Fig. 5 [file 44319_2025_551_MOESM7_ESM.zip › Fig5/Fig5F/STING/STING DAPI.tif]

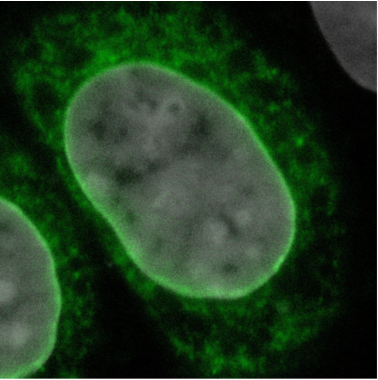

Supplement: Supplementary file 7 — Source data Fig. 5 [file 44319_2025_551_MOESM7_ESM.zip › Fig5/Fig5F/STING/STING Merge.tif]

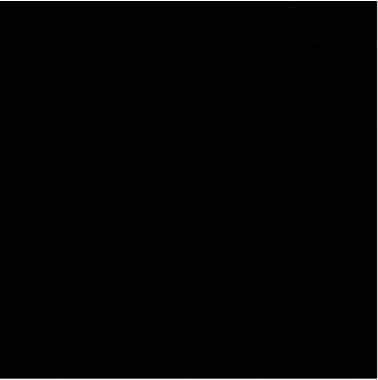

Supplement: Supplementary file 7 — Source data Fig. 5 [file 44319_2025_551_MOESM7_ESM.zip › Fig5/Fig5F/STING/STING ORF1p.tif]

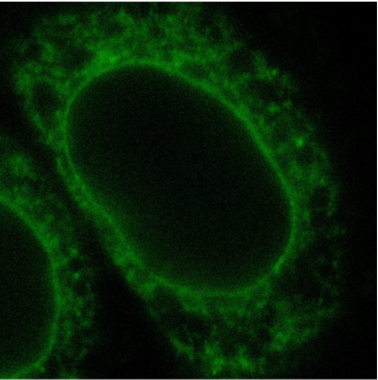

Supplement: Supplementary file 7 — Source data Fig. 5 [file 44319_2025_551_MOESM7_ESM.zip › Fig5/Fig5F/STING/STING STING.tif]

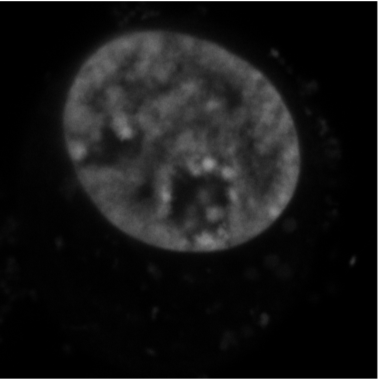

Supplement: Supplementary file 7 — Source data Fig. 5 [file 44319_2025_551_MOESM7_ESM.zip › Fig5/Fig5F/STING+L1/STING+L1 DAPI.tif]
