# Supplementary figures and images for: STING inhibits LINE-1 retrotransposition through sorting ORF1p to lysosomes for degradation (part 3 of 4)
Source: EMBO Rep. 2025 Aug 18;26(18):4607–30. doi: 10.1038/s44319-025-00551-0 (PMC12457603; doi:10.1038/s44319-025-00551-0)

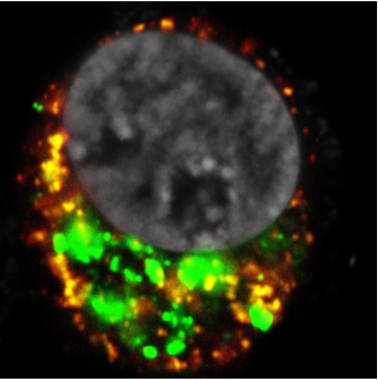

Supplement: Supplementary file 7 — Source data Fig. 5 [file 44319_2025_551_MOESM7_ESM.zip › Fig5/Fig5F/STING+L1/STING+L1 Merge.tif]

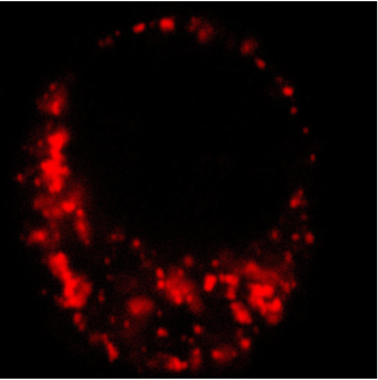

Supplement: Supplementary file 7 — Source data Fig. 5 [file 44319_2025_551_MOESM7_ESM.zip › Fig5/Fig5F/STING+L1/STING+L1 ORF1p.tif]

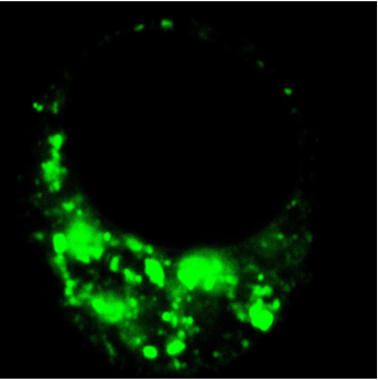

Supplement: Supplementary file 7 — Source data Fig. 5 [file 44319_2025_551_MOESM7_ESM.zip › Fig5/Fig5F/STING+L1/STING+L1 STING.tif]

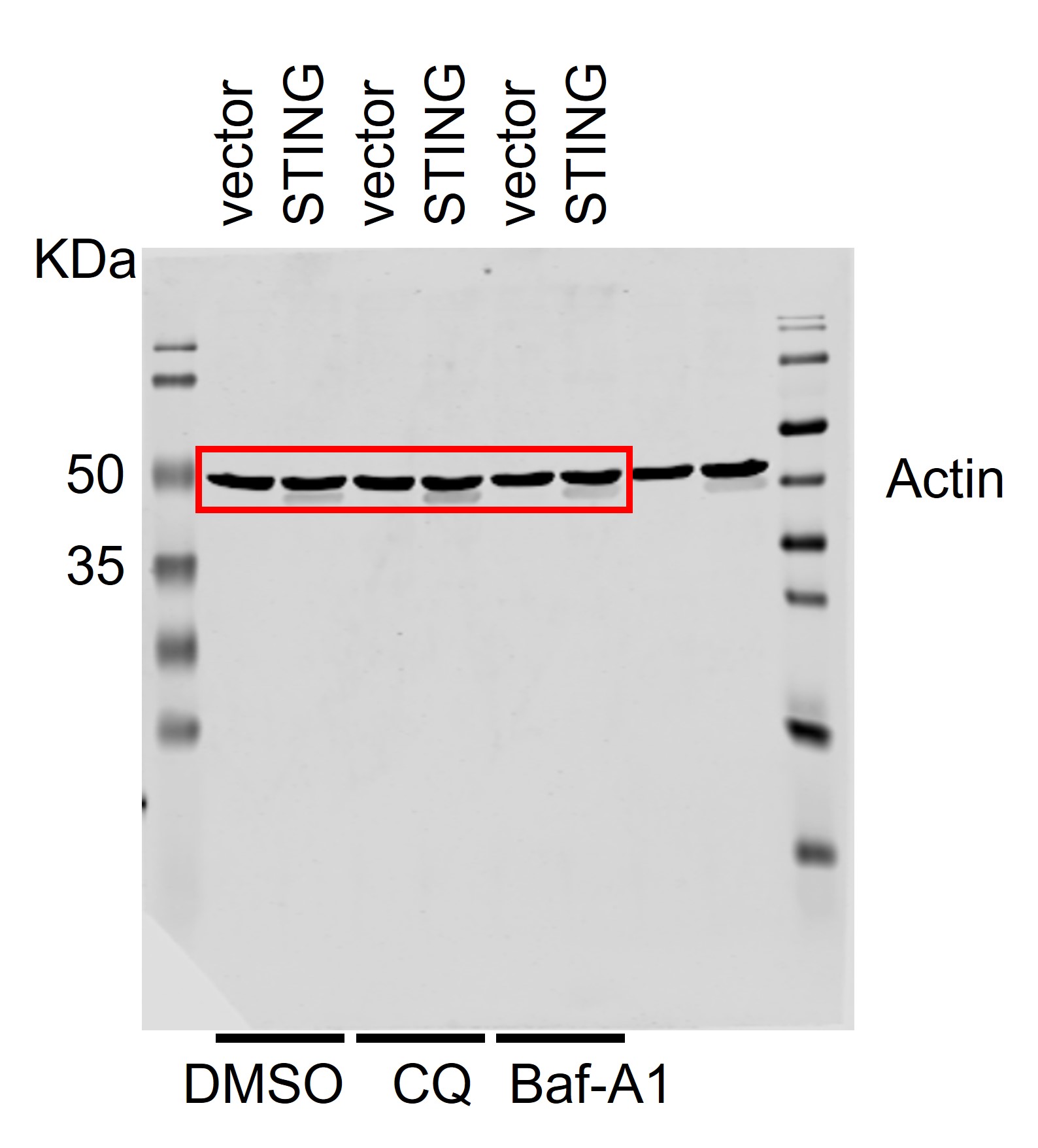

Supplement: Supplementary file 7 — Source data Fig. 5 [file 44319_2025_551_MOESM7_ESM.zip › Fig5/Fig5G/Fig5G IB Actin.jpg]

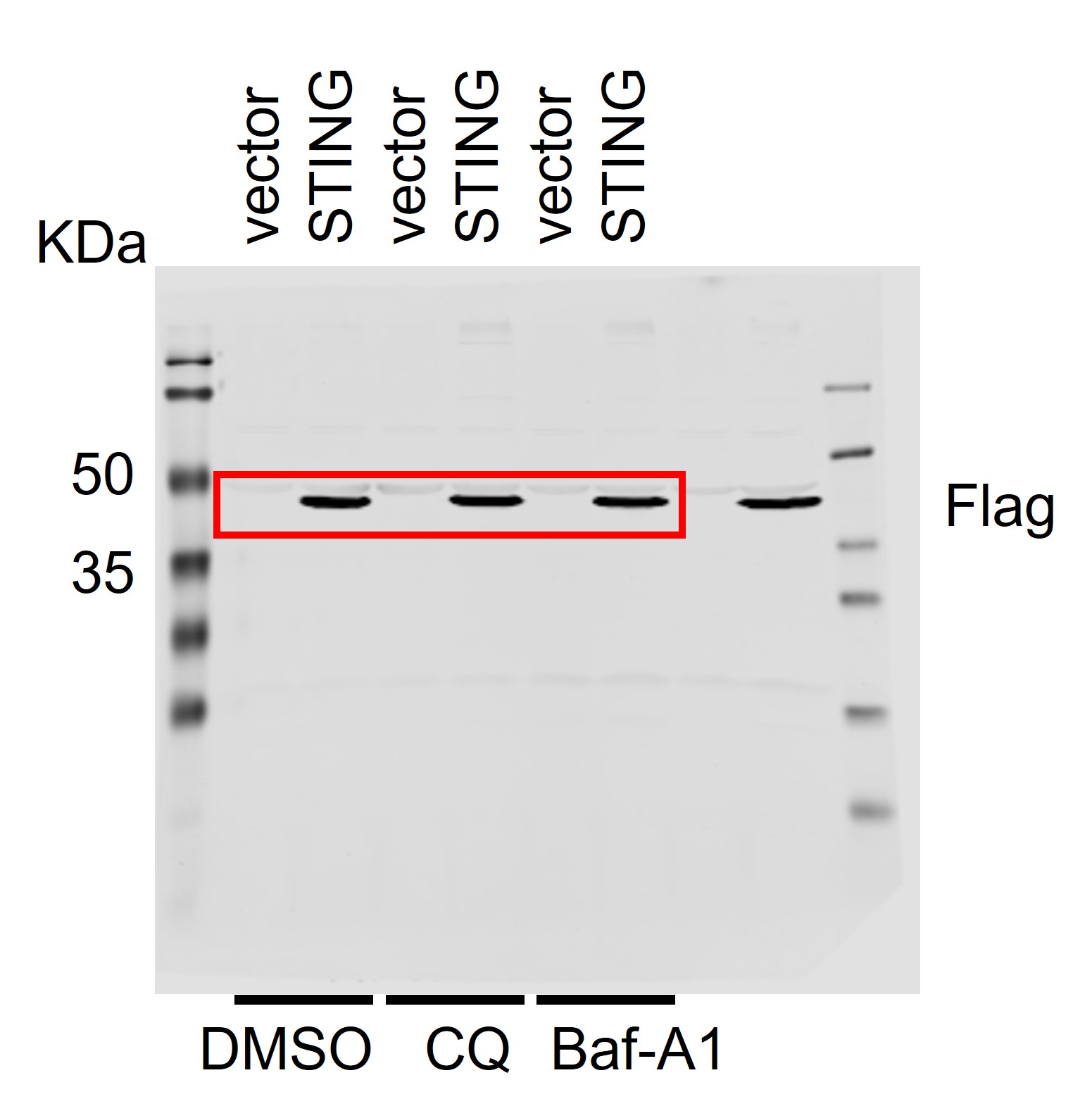

Supplement: Supplementary file 7 — Source data Fig. 5 [file 44319_2025_551_MOESM7_ESM.zip › Fig5/Fig5G/Fig5G IB Flag.jpg]

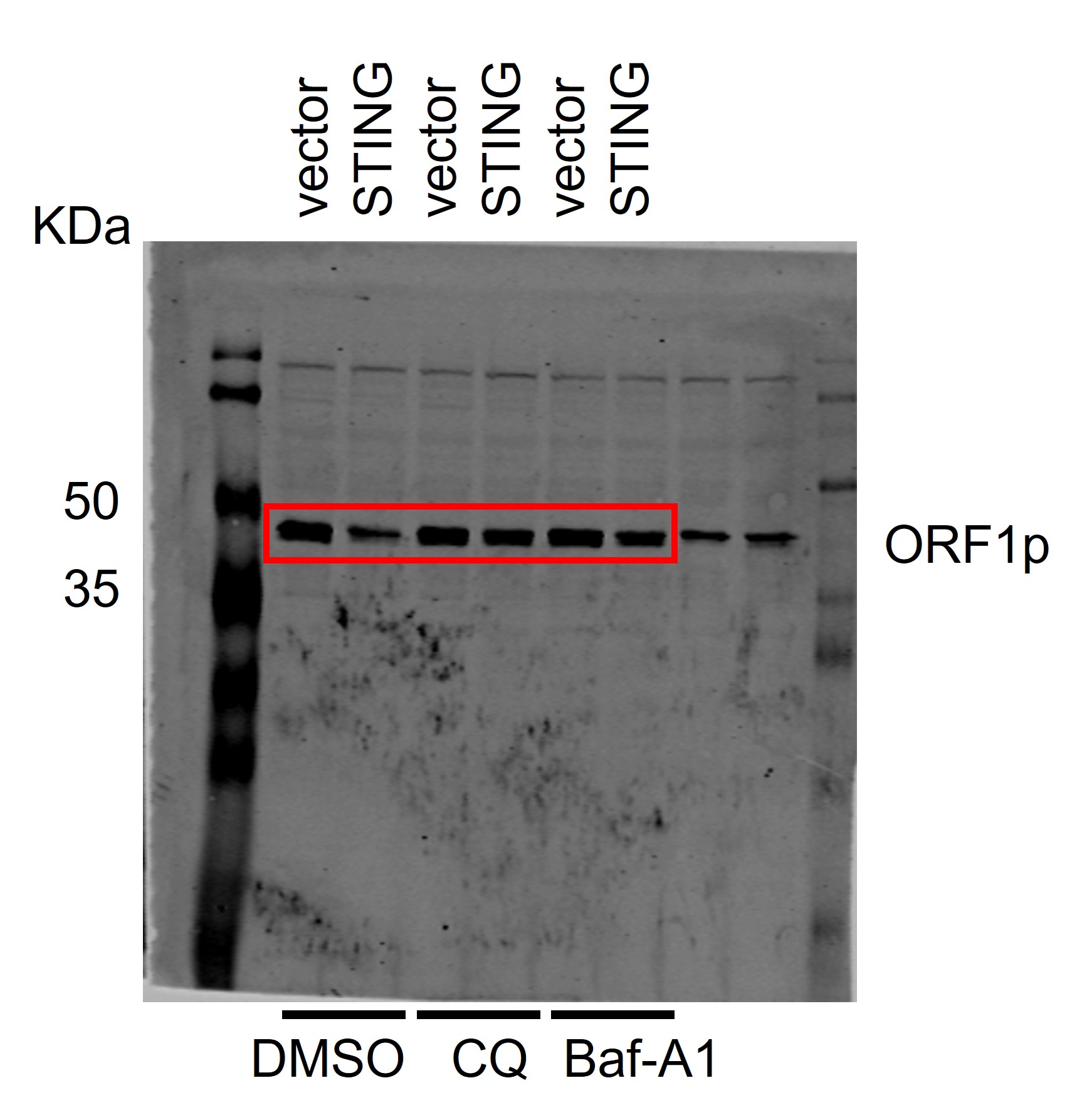

Supplement: Supplementary file 7 — Source data Fig. 5 [file 44319_2025_551_MOESM7_ESM.zip › Fig5/Fig5G/Fig5G IB ORF1p.jpg]

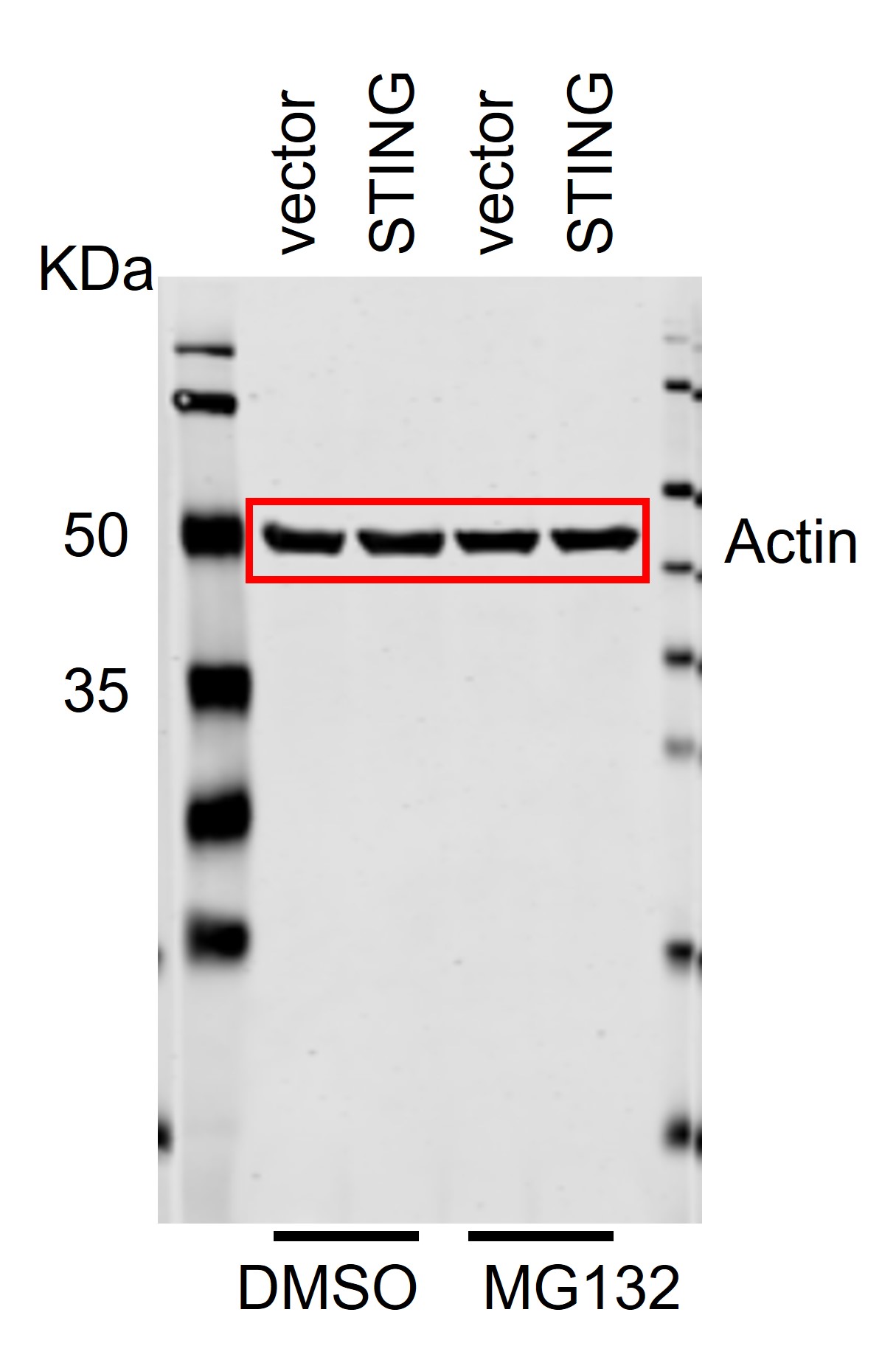

Supplement: Supplementary file 7 — Source data Fig. 5 [file 44319_2025_551_MOESM7_ESM.zip › Fig5/Fig5H/Fig5H Actin.jpg]

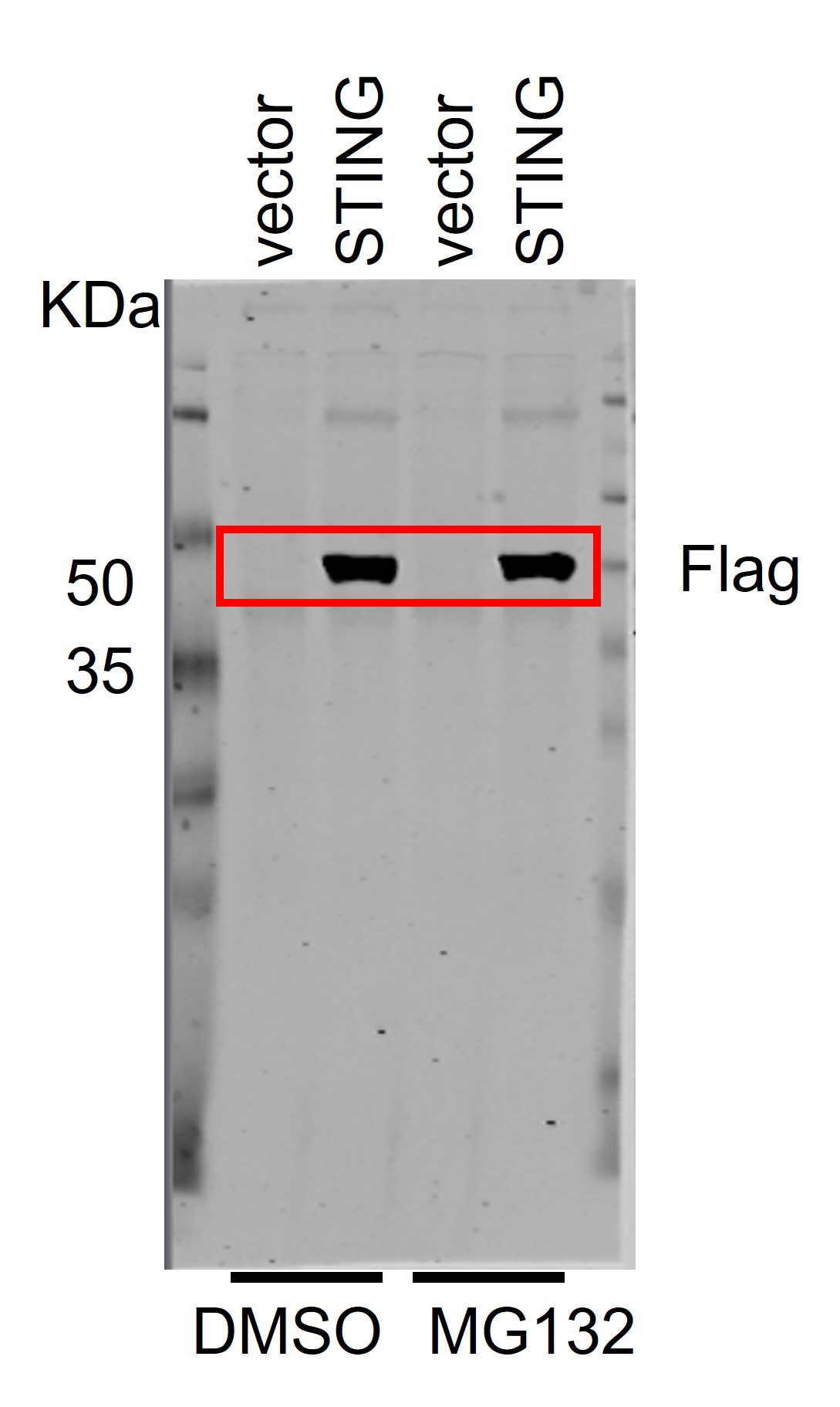

Supplement: Supplementary file 7 — Source data Fig. 5 [file 44319_2025_551_MOESM7_ESM.zip › Fig5/Fig5H/Fig5H Flag.jpg]

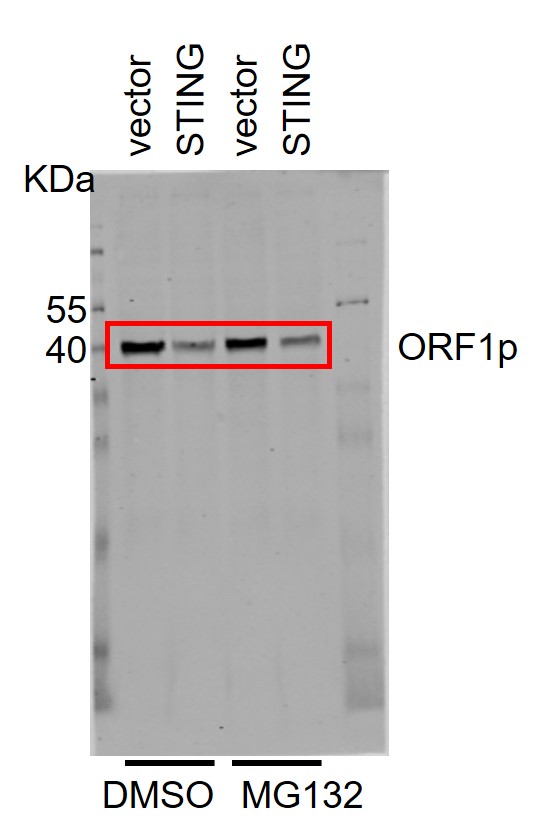

Supplement: Supplementary file 7 — Source data Fig. 5 [file 44319_2025_551_MOESM7_ESM.zip › Fig5/Fig5H/Fig5H ORF1p.jpg]

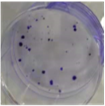

Supplement: Supplementary file 7 — Source data Fig. 5 [file 44319_2025_551_MOESM7_ESM.zip › Fig5/Fig5I/Fig5I image/333 334AA.tif]

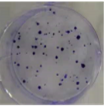

Supplement: Supplementary file 7 — Source data Fig. 5 [file 44319_2025_551_MOESM7_ESM.zip › Fig5/Fig5I/Fig5I image/STING.tif]

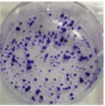

Supplement: Supplementary file 7 — Source data Fig. 5 [file 44319_2025_551_MOESM7_ESM.zip › Fig5/Fig5I/Fig5I image/Vector.tif]

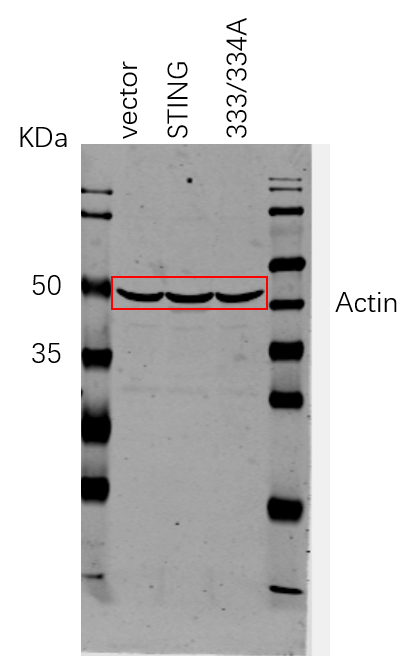

Supplement: Supplementary file 7 — Source data Fig. 5 [file 44319_2025_551_MOESM7_ESM.zip › Fig5/Fig5J/Fig5J IB Actin.tif]

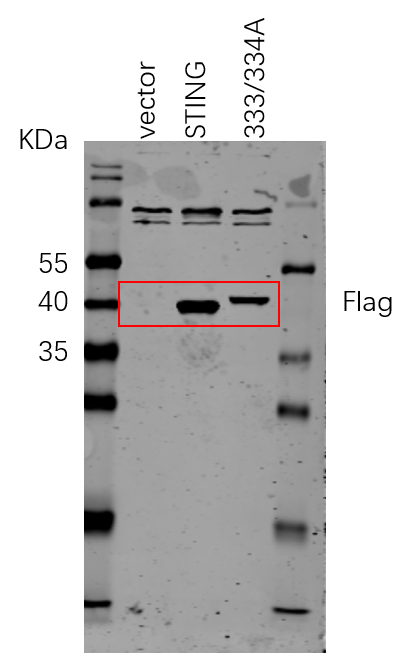

Supplement: Supplementary file 7 — Source data Fig. 5 [file 44319_2025_551_MOESM7_ESM.zip › Fig5/Fig5J/Fig5J IB Flag.tif]

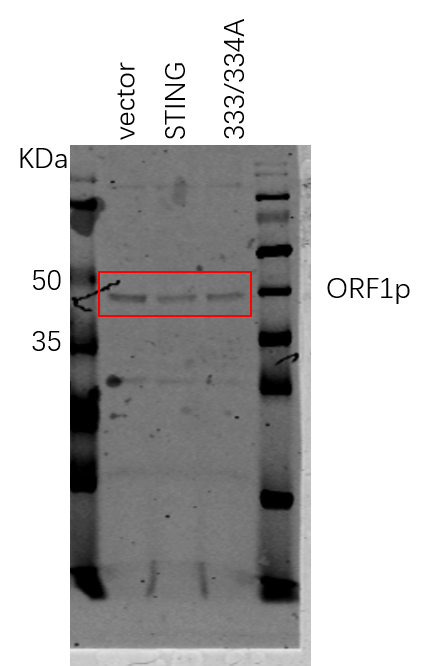

Supplement: Supplementary file 7 — Source data Fig. 5 [file 44319_2025_551_MOESM7_ESM.zip › Fig5/Fig5J/Fig5J IB ORF1p.tif]

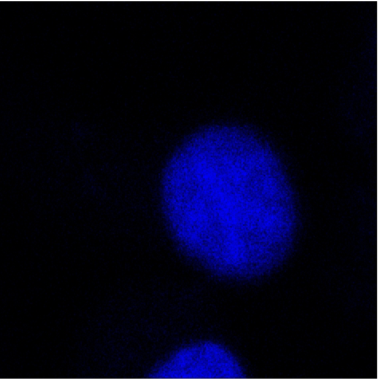

Supplement: Supplementary file 7 — Source data Fig. 5 [file 44319_2025_551_MOESM7_ESM.zip › Fig5/Fig5K/ST-Flag BafA1/ST-Flag BafA1 DAPI.tif]

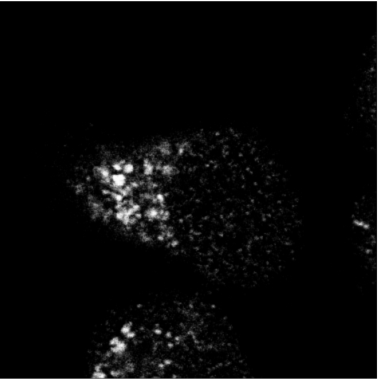

Supplement: Supplementary file 7 — Source data Fig. 5 [file 44319_2025_551_MOESM7_ESM.zip › Fig5/Fig5K/ST-Flag BafA1/ST-Flag BafA1 LC3.tif]

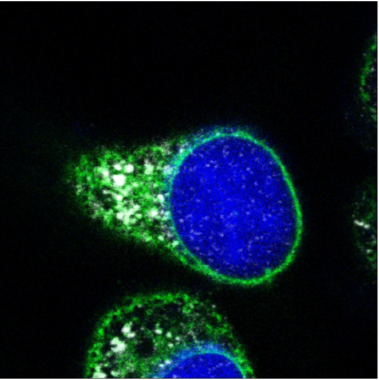

Supplement: Supplementary file 7 — Source data Fig. 5 [file 44319_2025_551_MOESM7_ESM.zip › Fig5/Fig5K/ST-Flag BafA1/ST-Flag BafA1 Merge.tif]

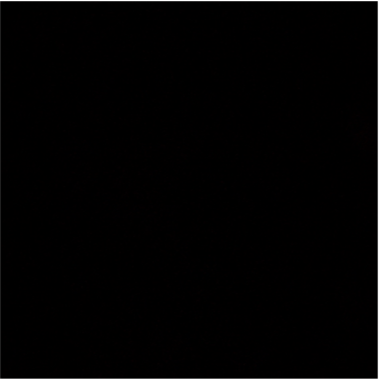

Supplement: Supplementary file 7 — Source data Fig. 5 [file 44319_2025_551_MOESM7_ESM.zip › Fig5/Fig5K/ST-Flag BafA1/ST-Flag BafA1 ORF1p.tif]

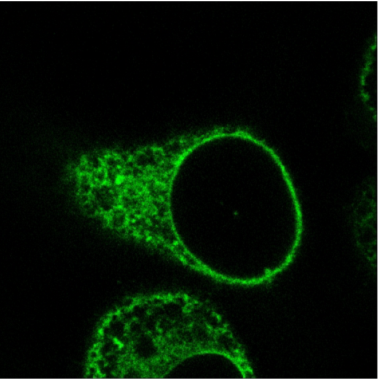

Supplement: Supplementary file 7 — Source data Fig. 5 [file 44319_2025_551_MOESM7_ESM.zip › Fig5/Fig5K/ST-Flag BafA1/ST-Flag BafA1 STING.tif]

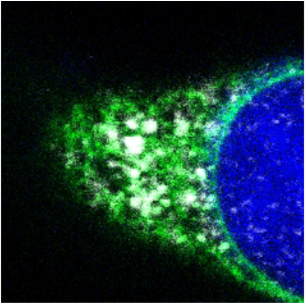

Supplement: Supplementary file 7 — Source data Fig. 5 [file 44319_2025_551_MOESM7_ESM.zip › Fig5/Fig5K/ST-Flag BafA1/ST-Flag BafA1 Zoom .tif]

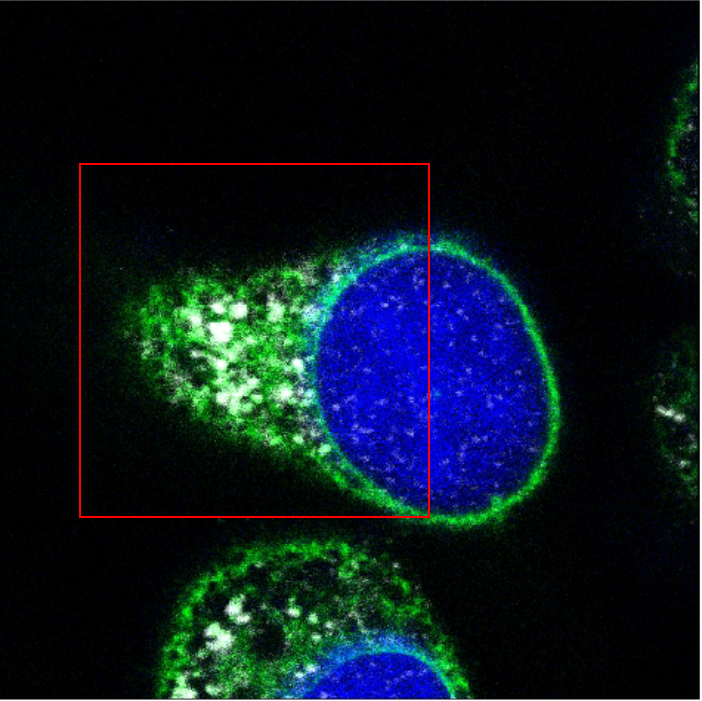

Supplement: Supplementary file 7 — Source data Fig. 5 [file 44319_2025_551_MOESM7_ESM.zip › Fig5/Fig5K/ST-Flag BafA1/ST-Flag BafA1 Zoom area.tif]

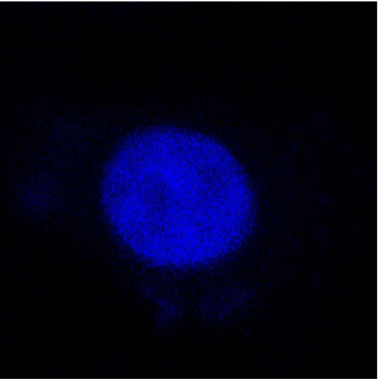

Supplement: Supplementary file 7 — Source data Fig. 5 [file 44319_2025_551_MOESM7_ESM.zip › Fig5/Fig5K/ST-Flag+L1/ST-Flag+L1 DAPI.tif]

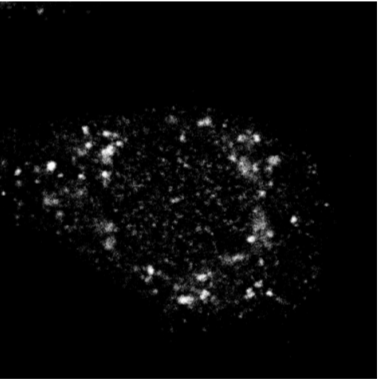

Supplement: Supplementary file 7 — Source data Fig. 5 [file 44319_2025_551_MOESM7_ESM.zip › Fig5/Fig5K/ST-Flag+L1/ST-Flag+L1 LC3.tif]

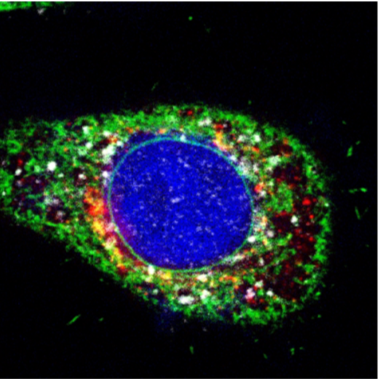

Supplement: Supplementary file 7 — Source data Fig. 5 [file 44319_2025_551_MOESM7_ESM.zip › Fig5/Fig5K/ST-Flag+L1/ST-Flag+L1 Merge.tif]

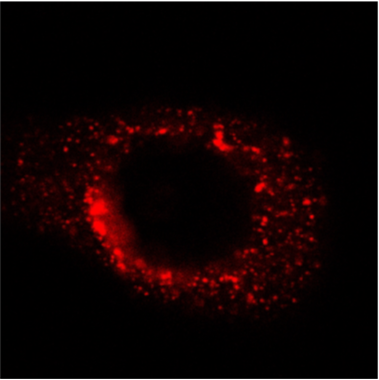

Supplement: Supplementary file 7 — Source data Fig. 5 [file 44319_2025_551_MOESM7_ESM.zip › Fig5/Fig5K/ST-Flag+L1/ST-Flag+L1 ORF1p.tif]

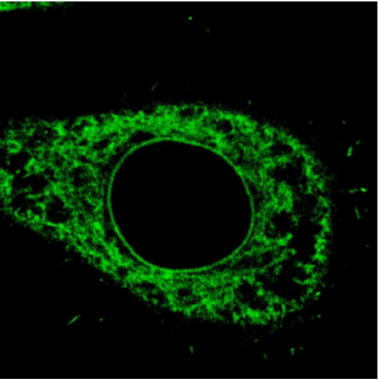

Supplement: Supplementary file 7 — Source data Fig. 5 [file 44319_2025_551_MOESM7_ESM.zip › Fig5/Fig5K/ST-Flag+L1/ST-Flag+L1 STING.tif]

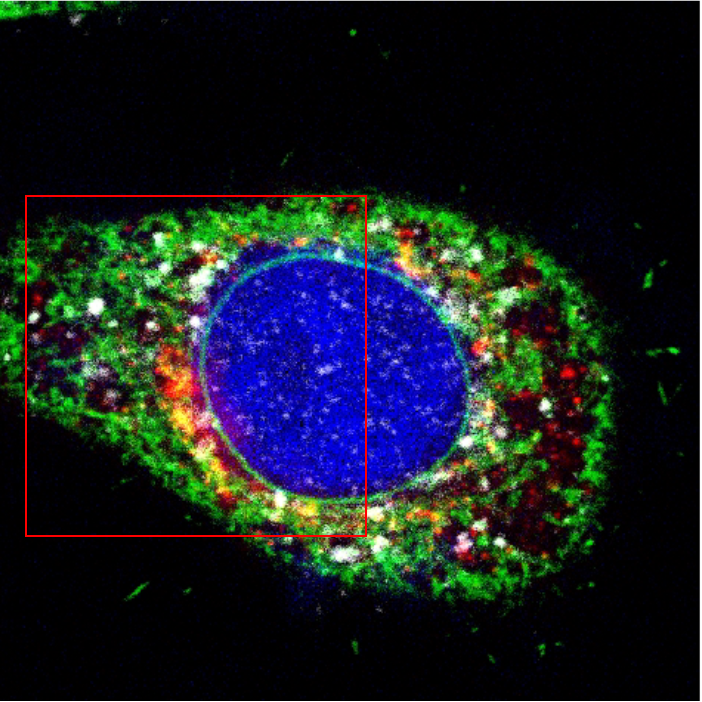

Supplement: Supplementary file 7 — Source data Fig. 5 [file 44319_2025_551_MOESM7_ESM.zip › Fig5/Fig5K/ST-Flag+L1/ST-Flag+L1 Zoom area.tif]

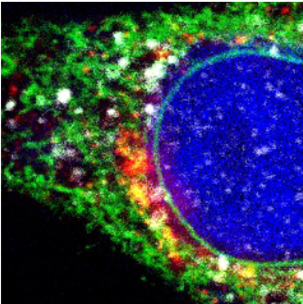

Supplement: Supplementary file 7 — Source data Fig. 5 [file 44319_2025_551_MOESM7_ESM.zip › Fig5/Fig5K/ST-Flag+L1/ST-Flag+L1 Zoom.tif]

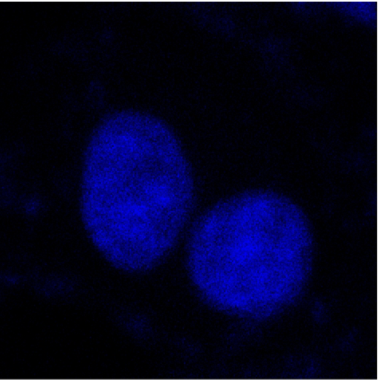

Supplement: Supplementary file 7 — Source data Fig. 5 [file 44319_2025_551_MOESM7_ESM.zip › Fig5/Fig5K/ST-Flag+L1 BafA1/ST-Flag+L1 BafA1 DAPI.tif]

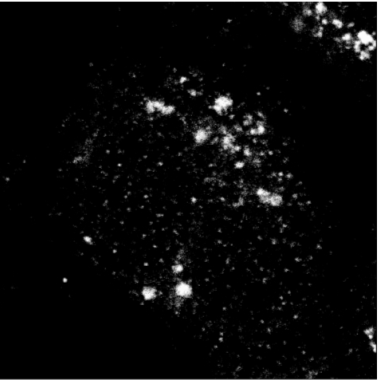

Supplement: Supplementary file 7 — Source data Fig. 5 [file 44319_2025_551_MOESM7_ESM.zip › Fig5/Fig5K/ST-Flag+L1 BafA1/ST-Flag+L1 BafA1 LC3.tif]

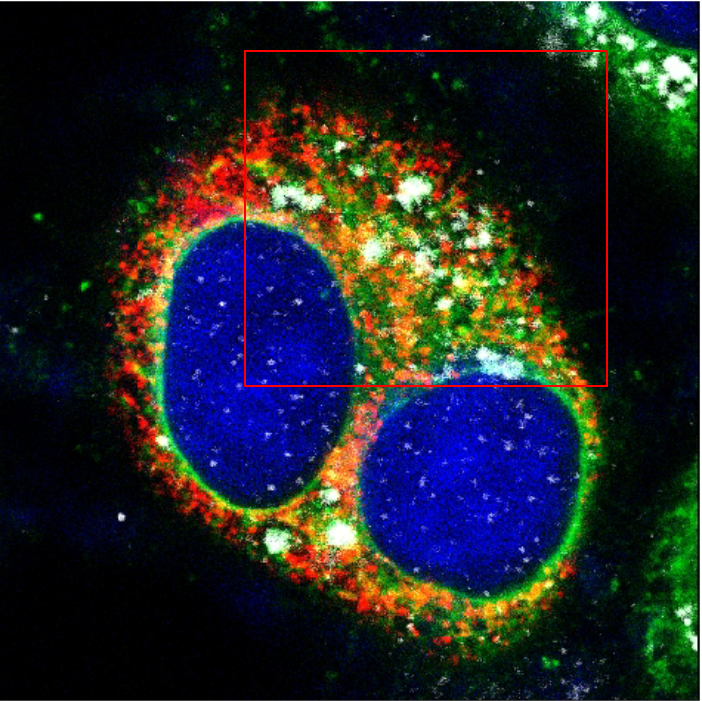

Supplement: Supplementary file 7 — Source data Fig. 5 [file 44319_2025_551_MOESM7_ESM.zip › Fig5/Fig5K/ST-Flag+L1 BafA1/ST-Flag+L1 BafA1 Merge Zoom area.tif]

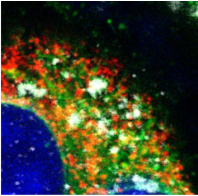

Supplement: Supplementary file 7 — Source data Fig. 5 [file 44319_2025_551_MOESM7_ESM.zip › Fig5/Fig5K/ST-Flag+L1 BafA1/ST-Flag+L1 BafA1 Merge Zoom.tif]

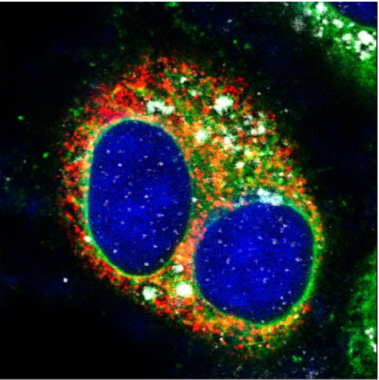

Supplement: Supplementary file 7 — Source data Fig. 5 [file 44319_2025_551_MOESM7_ESM.zip › Fig5/Fig5K/ST-Flag+L1 BafA1/ST-Flag+L1 BafA1 Merge.tif]

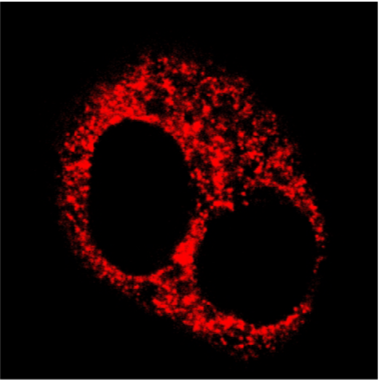

Supplement: Supplementary file 7 — Source data Fig. 5 [file 44319_2025_551_MOESM7_ESM.zip › Fig5/Fig5K/ST-Flag+L1 BafA1/ST-Flag+L1 BafA1 ORF1p.tif]

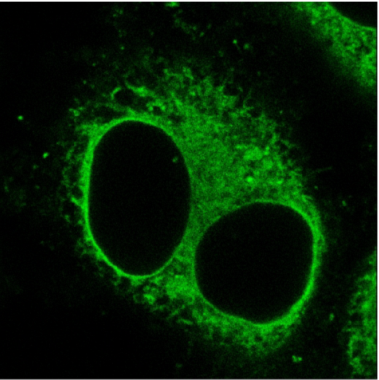

Supplement: Supplementary file 7 — Source data Fig. 5 [file 44319_2025_551_MOESM7_ESM.zip › Fig5/Fig5K/ST-Flag+L1 BafA1/ST-Flag+L1 BafA1 STING.tif]

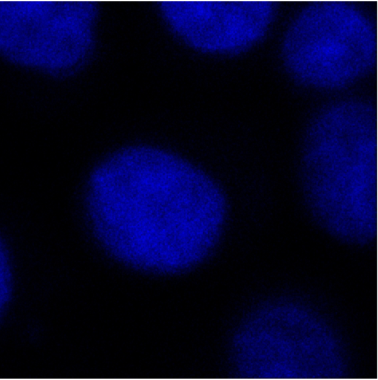

Supplement: Supplementary file 7 — Source data Fig. 5 [file 44319_2025_551_MOESM7_ESM.zip › Fig5/Fig5K/STING-Flag/STING-Flag DAPI.tif]

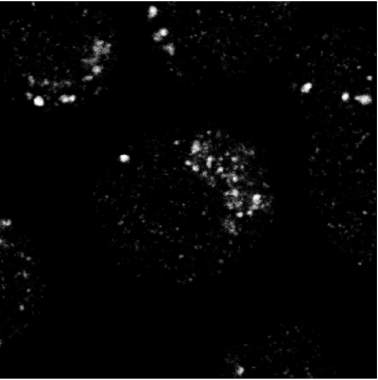

Supplement: Supplementary file 7 — Source data Fig. 5 [file 44319_2025_551_MOESM7_ESM.zip › Fig5/Fig5K/STING-Flag/STING-Flag LC3.tif]

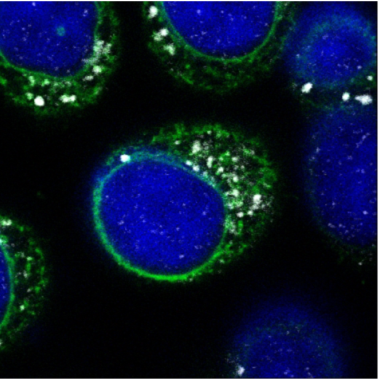

Supplement: Supplementary file 7 — Source data Fig. 5 [file 44319_2025_551_MOESM7_ESM.zip › Fig5/Fig5K/STING-Flag/STING-Flag Merge.tif]

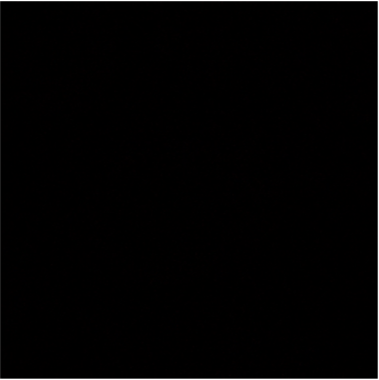

Supplement: Supplementary file 7 — Source data Fig. 5 [file 44319_2025_551_MOESM7_ESM.zip › Fig5/Fig5K/STING-Flag/STING-Flag ORF1p.tif]

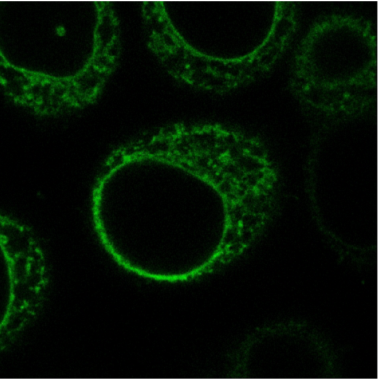

Supplement: Supplementary file 7 — Source data Fig. 5 [file 44319_2025_551_MOESM7_ESM.zip › Fig5/Fig5K/STING-Flag/STING-Flag STING.tif]

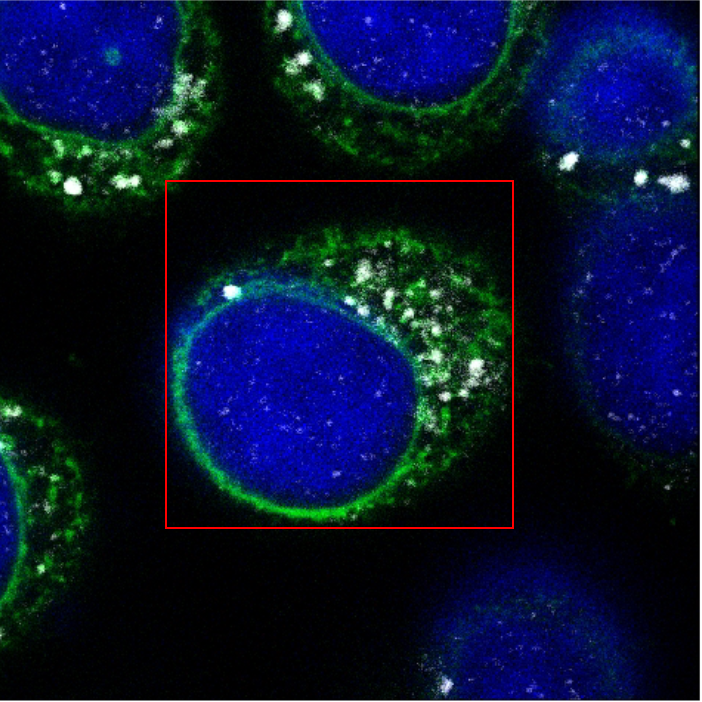

Supplement: Supplementary file 7 — Source data Fig. 5 [file 44319_2025_551_MOESM7_ESM.zip › Fig5/Fig5K/STING-Flag/STING-Flag Zoom area.tif]

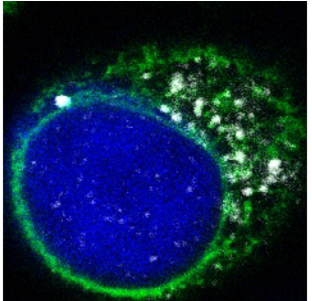

Supplement: Supplementary file 7 — Source data Fig. 5 [file 44319_2025_551_MOESM7_ESM.zip › Fig5/Fig5K/STING-Flag/STING-Flag Zoom.tif]

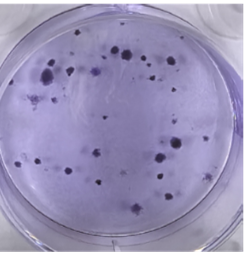

Supplement: Supplementary file 7 — Source data Fig. 5 [file 44319_2025_551_MOESM7_ESM.zip › Fig5/Fig5L/ATG5KO1 STING.png]

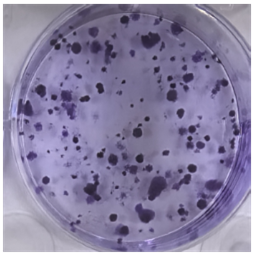

Supplement: Supplementary file 7 — Source data Fig. 5 [file 44319_2025_551_MOESM7_ESM.zip › Fig5/Fig5L/ATG5KO1 vector.png]

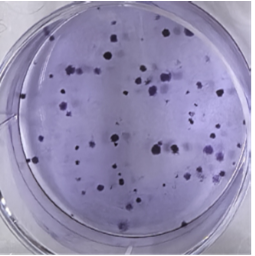

Supplement: Supplementary file 7 — Source data Fig. 5 [file 44319_2025_551_MOESM7_ESM.zip › Fig5/Fig5L/ATG5KO2 STING.png]

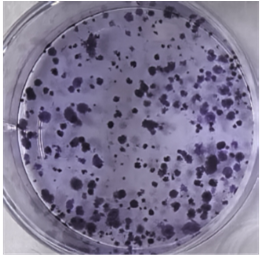

Supplement: Supplementary file 7 — Source data Fig. 5 [file 44319_2025_551_MOESM7_ESM.zip › Fig5/Fig5L/ATG5KO2 vector.png]

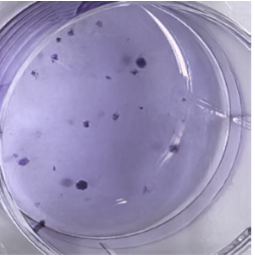

Supplement: Supplementary file 7 — Source data Fig. 5 [file 44319_2025_551_MOESM7_ESM.zip › Fig5/Fig5L/control STING.png]

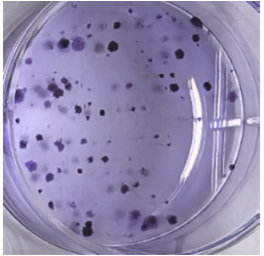

Supplement: Supplementary file 7 — Source data Fig. 5 [file 44319_2025_551_MOESM7_ESM.zip › Fig5/Fig5L/control vector.png]

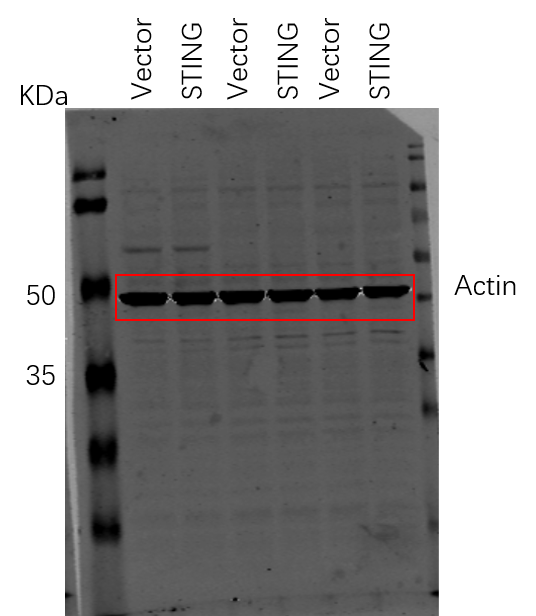

Supplement: Supplementary file 7 — Source data Fig. 5 [file 44319_2025_551_MOESM7_ESM.zip › Fig5/Fig5M/Fig5M IB Actin.png]

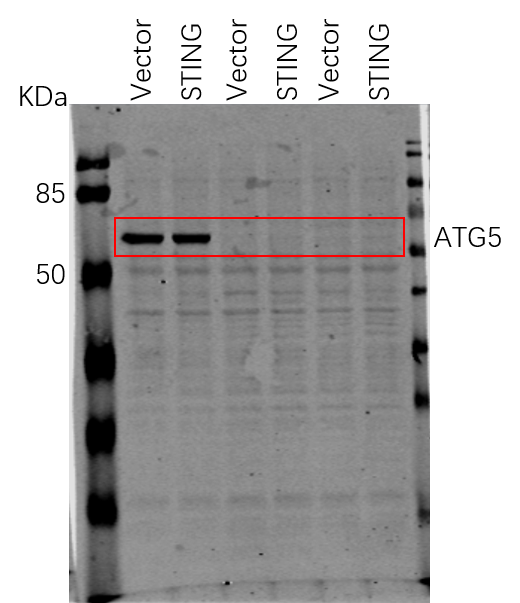

Supplement: Supplementary file 7 — Source data Fig. 5 [file 44319_2025_551_MOESM7_ESM.zip › Fig5/Fig5M/Fig5M IB ATG5.png]

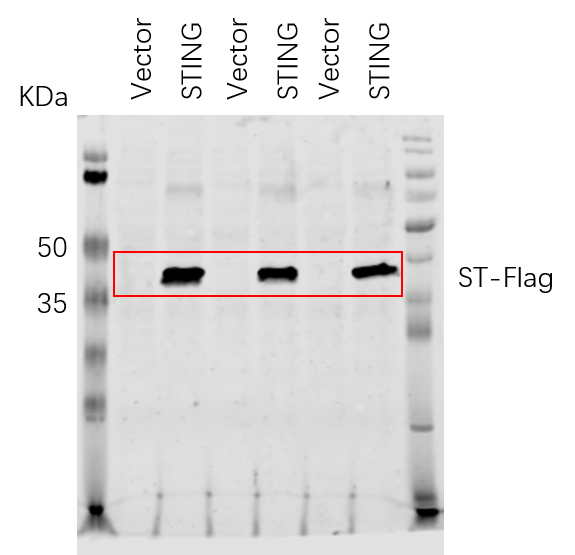

Supplement: Supplementary file 7 — Source data Fig. 5 [file 44319_2025_551_MOESM7_ESM.zip › Fig5/Fig5M/Fig5M IB Flag.png]

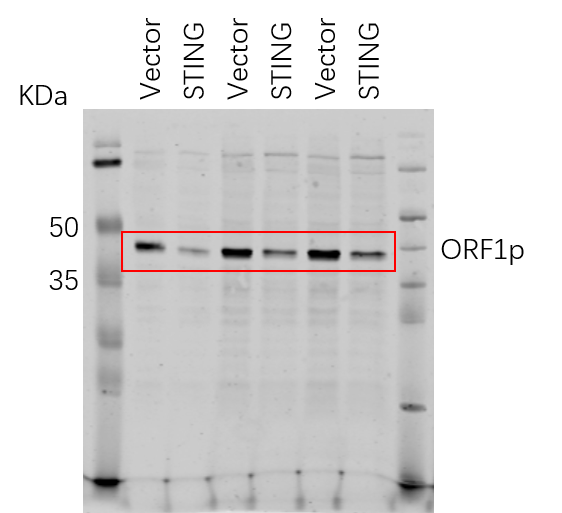

Supplement: Supplementary file 7 — Source data Fig. 5 [file 44319_2025_551_MOESM7_ESM.zip › Fig5/Fig5M/Fig5M IB ORF1p.png]

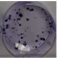

Supplement: Supplementary file 8 — Source data Fig. 6 [file 44319_2025_551_MOESM8_ESM.zip › Fig6/Fig6A/Fig6A image/E282A D283A.tif]

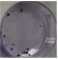

Supplement: Supplementary file 8 — Source data Fig. 6 [file 44319_2025_551_MOESM8_ESM.zip › Fig6/Fig6A/Fig6A image/E296A D297A.tif]

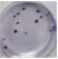

Supplement: Supplementary file 8 — Source data Fig. 6 [file 44319_2025_551_MOESM8_ESM.zip › Fig6/Fig6A/Fig6A image/L333A R334A.tif]

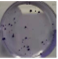

Supplement: Supplementary file 8 — Source data Fig. 6 [file 44319_2025_551_MOESM8_ESM.zip › Fig6/Fig6A/Fig6A image/STING.tif]

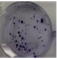

Supplement: Supplementary file 8 — Source data Fig. 6 [file 44319_2025_551_MOESM8_ESM.zip › Fig6/Fig6A/Fig6A image/vector.tif]

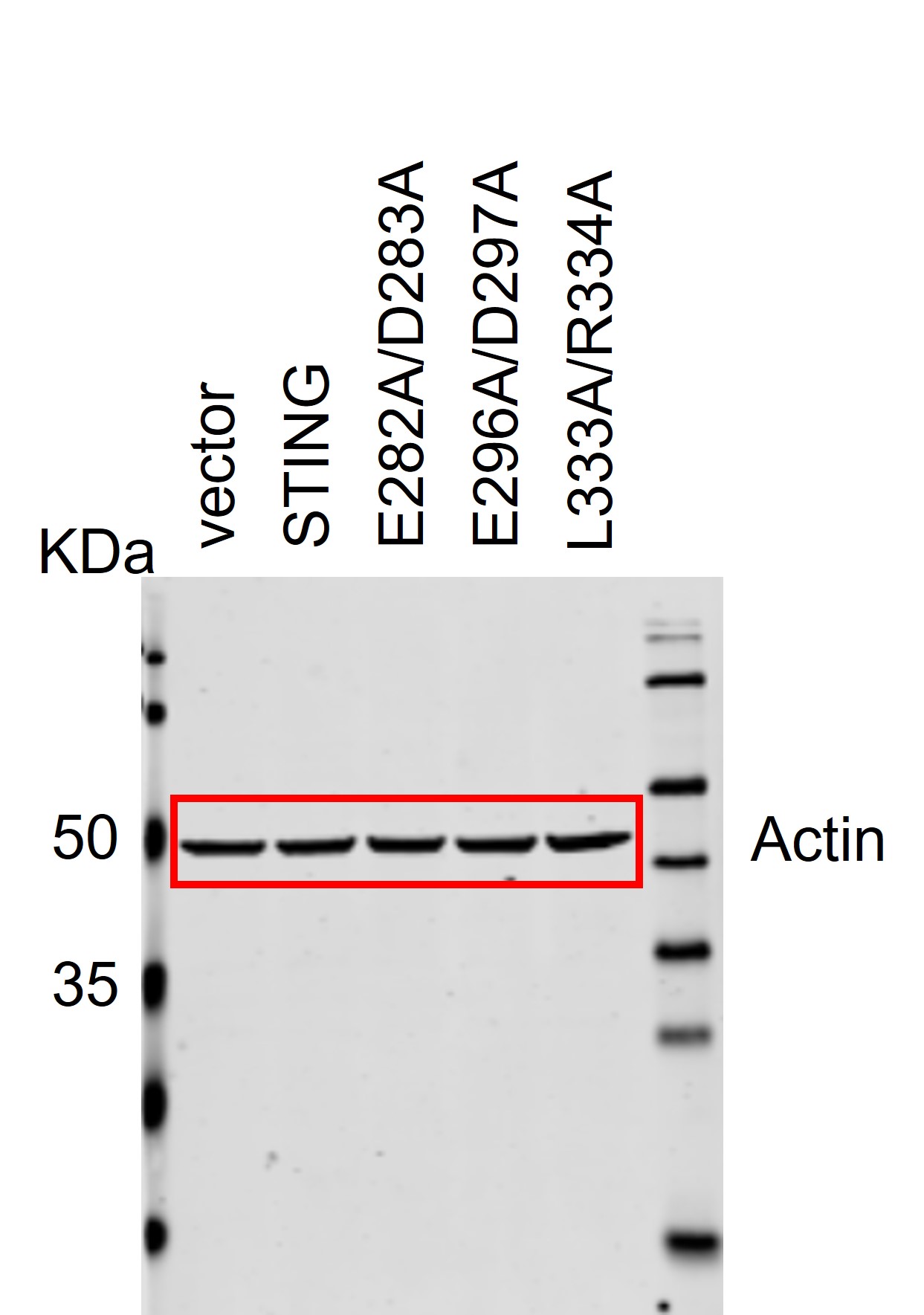

Supplement: Supplementary file 8 — Source data Fig. 6 [file 44319_2025_551_MOESM8_ESM.zip › Fig6/Fig6B/Fig6B Actin.jpg]

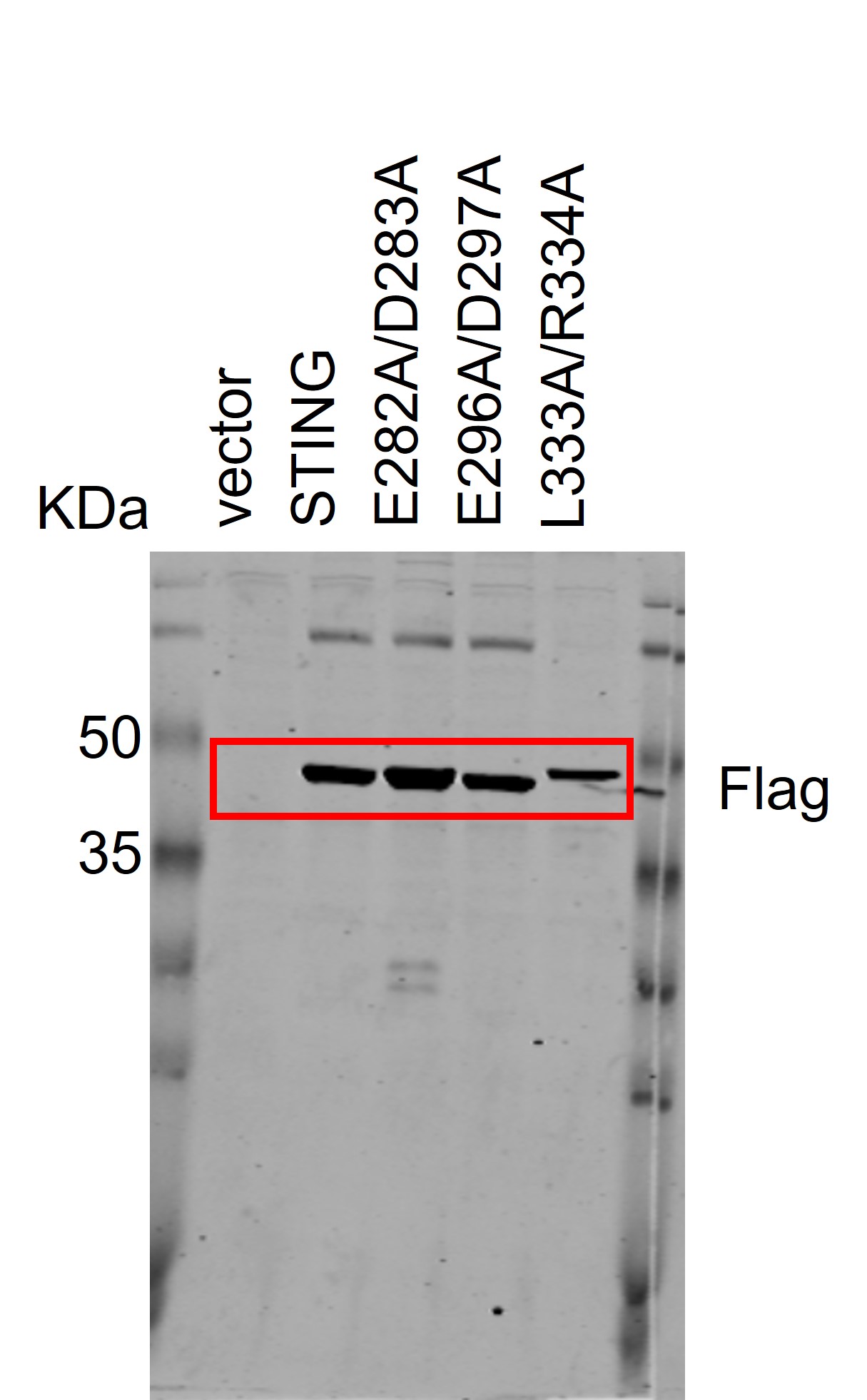

Supplement: Supplementary file 8 — Source data Fig. 6 [file 44319_2025_551_MOESM8_ESM.zip › Fig6/Fig6B/Fig6B Flag.jpg]

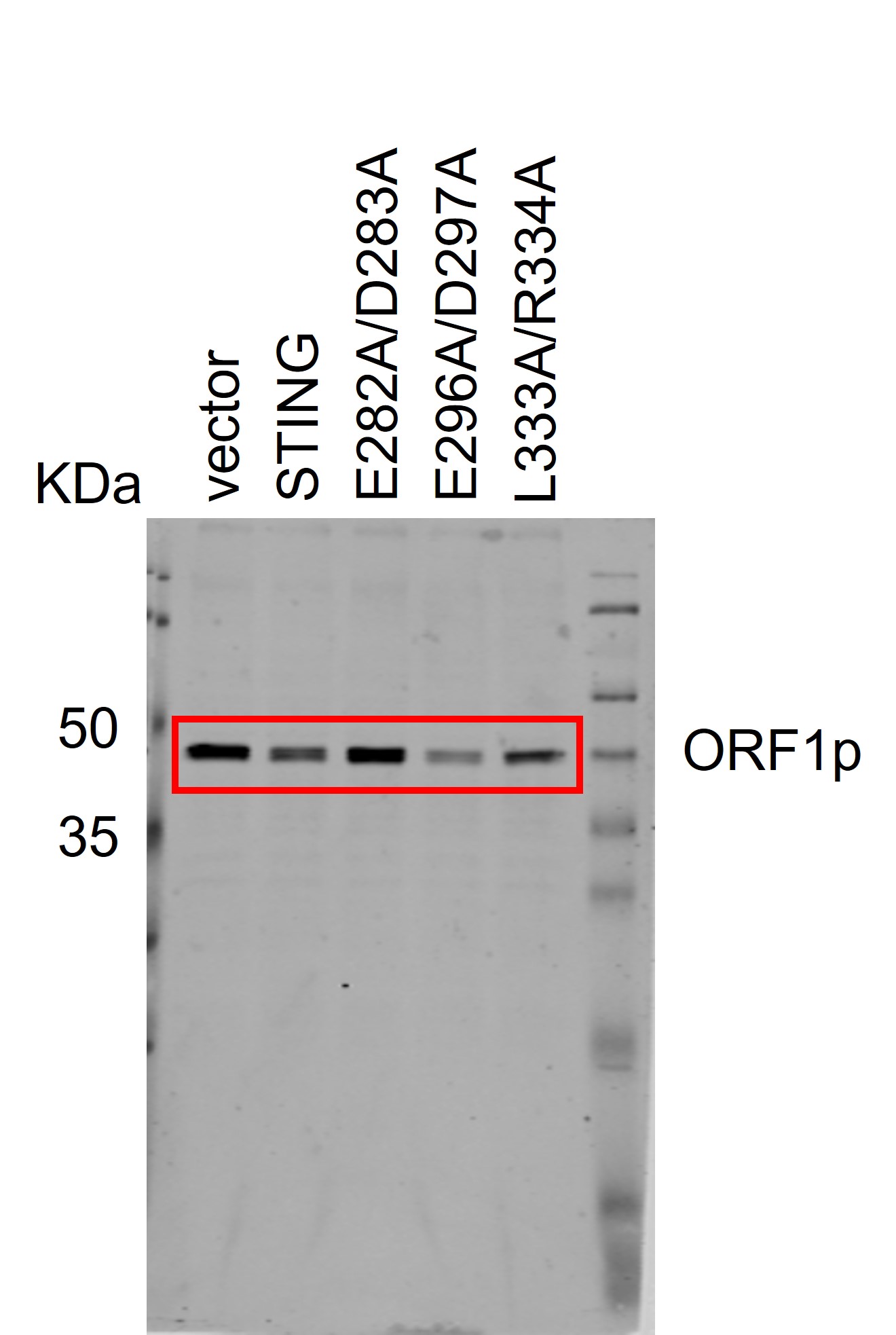

Supplement: Supplementary file 8 — Source data Fig. 6 [file 44319_2025_551_MOESM8_ESM.zip › Fig6/Fig6B/Fig6B ORF1p.jpg]

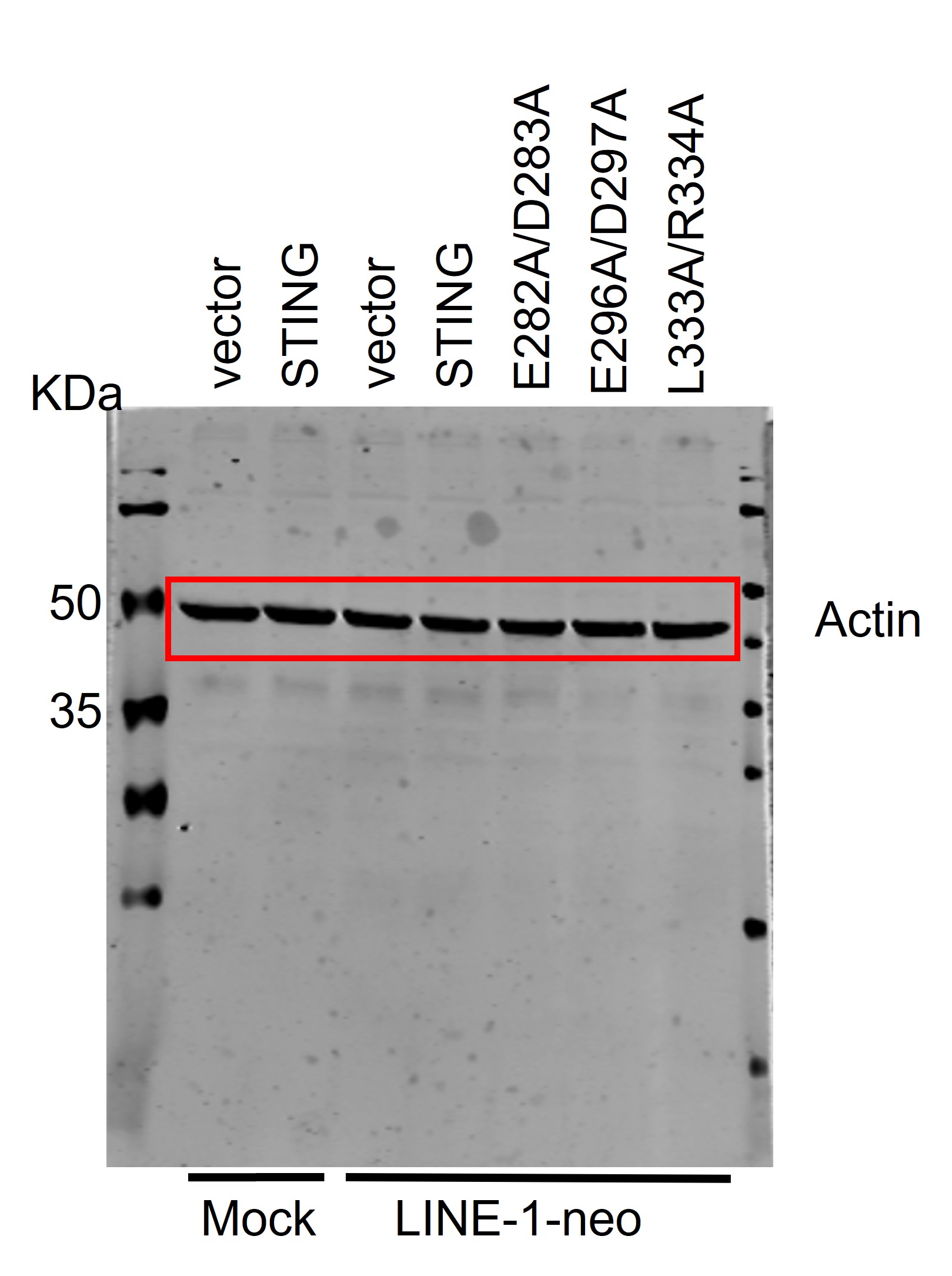

Supplement: Supplementary file 8 — Source data Fig. 6 [file 44319_2025_551_MOESM8_ESM.zip › Fig6/Fig6C/Fig6C Actin.jpg]

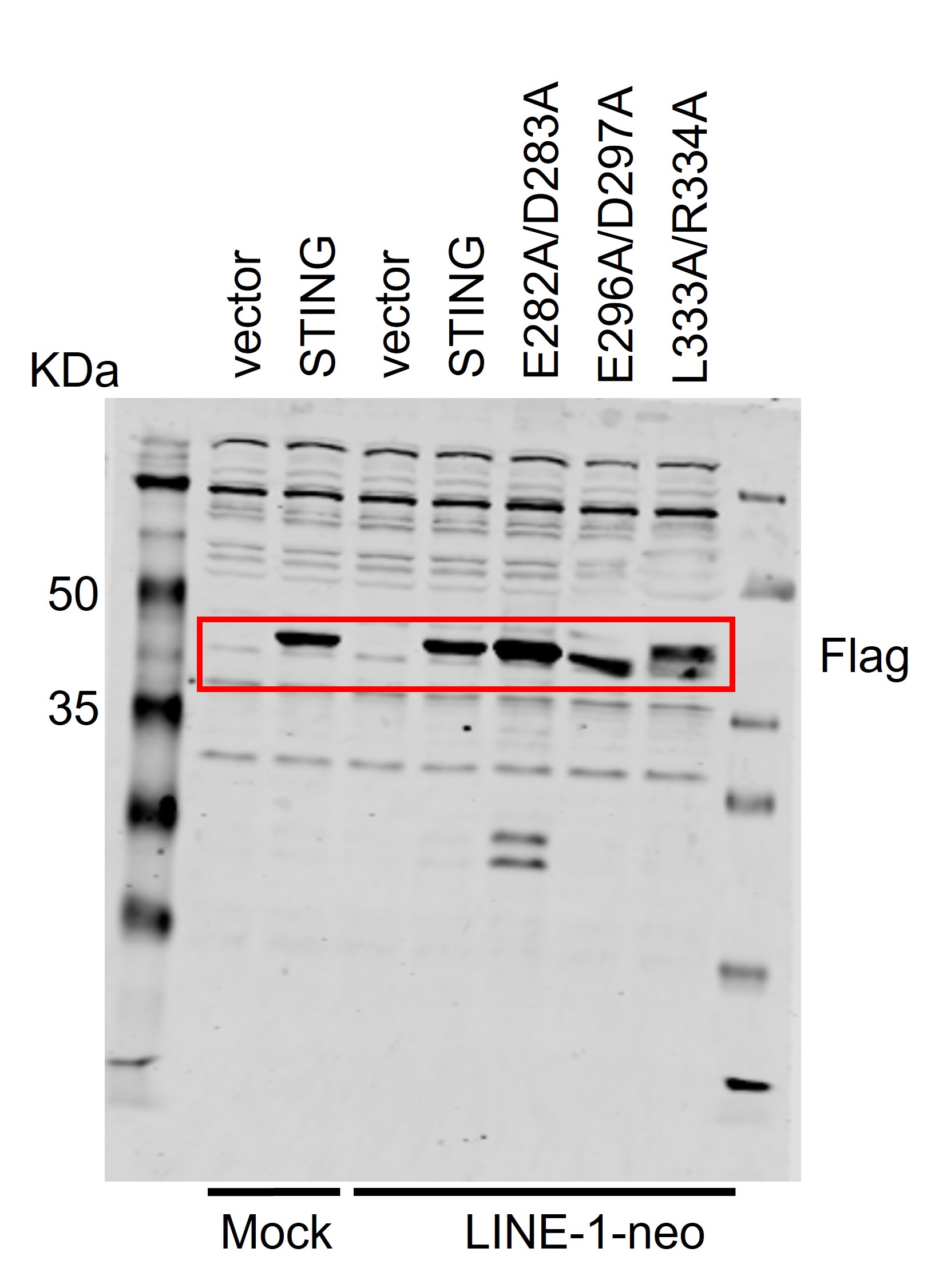

Supplement: Supplementary file 8 — Source data Fig. 6 [file 44319_2025_551_MOESM8_ESM.zip › Fig6/Fig6C/Fig6C Flag.jpg]

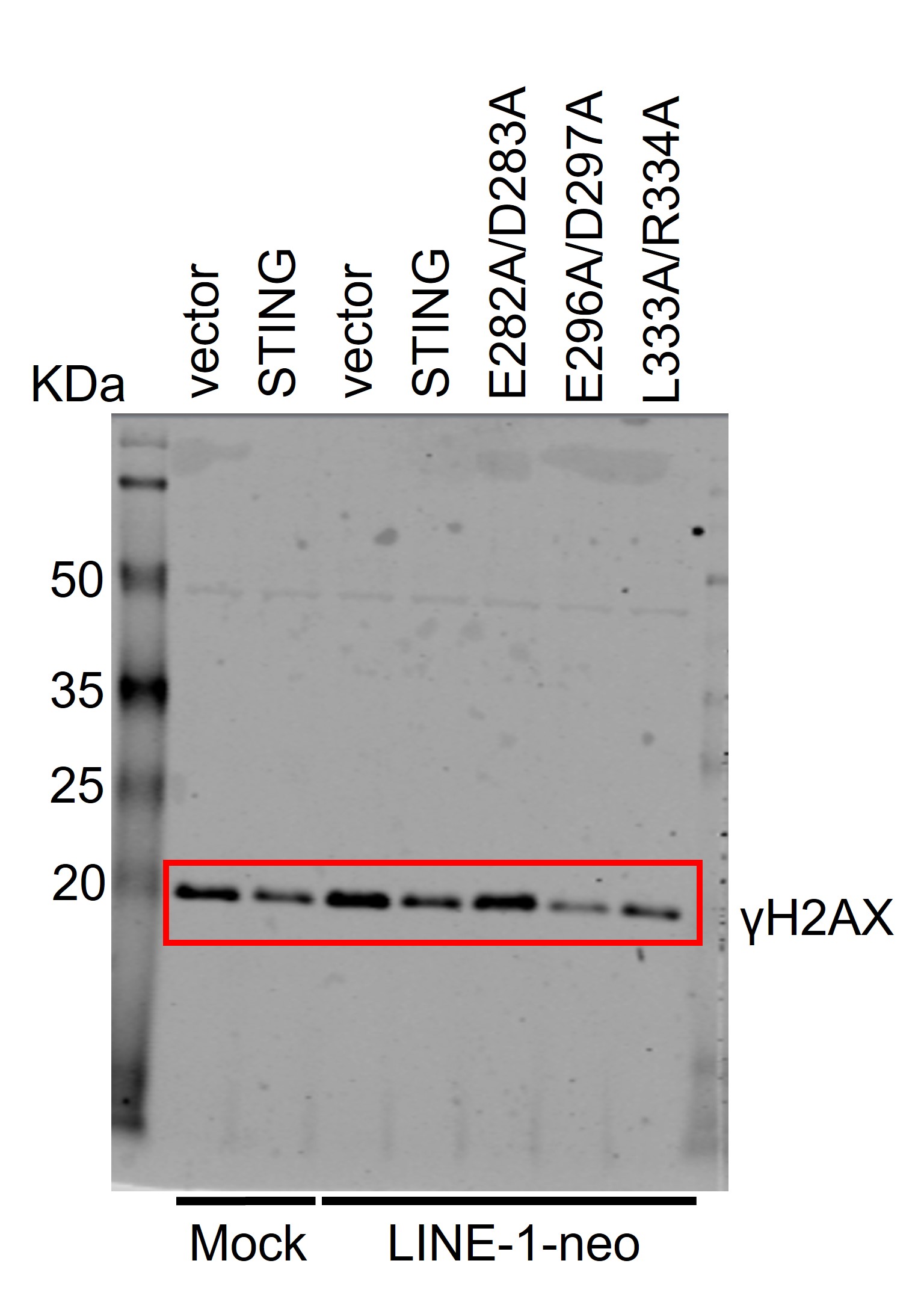

Supplement: Supplementary file 8 — Source data Fig. 6 [file 44319_2025_551_MOESM8_ESM.zip › Fig6/Fig6C/Fig6C γH2AX.jpg]

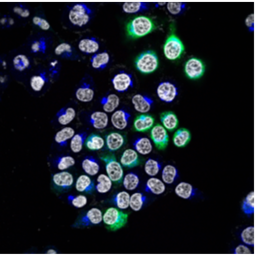

Supplement: Supplementary file 8 — Source data Fig. 6 [file 44319_2025_551_MOESM8_ESM.zip › Fig6/Fig6D/STING-EGFP/1X merge.png]

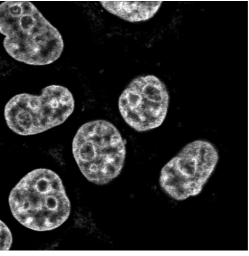

Supplement: Supplementary file 8 — Source data Fig. 6 [file 44319_2025_551_MOESM8_ESM.zip › Fig6/Fig6D/STING-EGFP/3X DAPI.png]

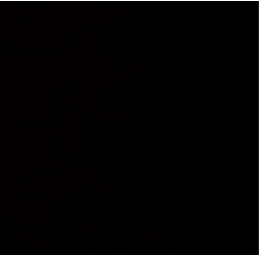

Supplement: Supplementary file 8 — Source data Fig. 6 [file 44319_2025_551_MOESM8_ESM.zip › Fig6/Fig6D/STING-EGFP/3X ORF1p.png]

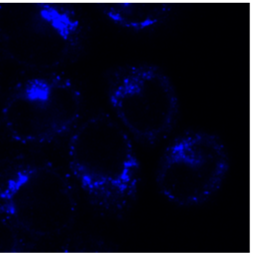

Supplement: Supplementary file 8 — Source data Fig. 6 [file 44319_2025_551_MOESM8_ESM.zip › Fig6/Fig6D/STING-EGFP/3X Rab7.png]

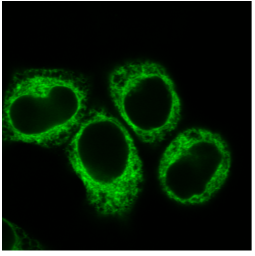

Supplement: Supplementary file 8 — Source data Fig. 6 [file 44319_2025_551_MOESM8_ESM.zip › Fig6/Fig6D/STING-EGFP/3X STING.png]

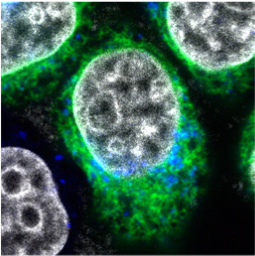

Supplement: Supplementary file 8 — Source data Fig. 6 [file 44319_2025_551_MOESM8_ESM.zip › Fig6/Fig6D/STING-EGFP/6X merge.png]

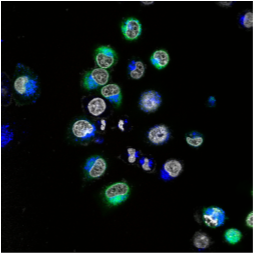

Supplement: Supplementary file 8 — Source data Fig. 6 [file 44319_2025_551_MOESM8_ESM.zip › Fig6/Fig6D/STING-EGFP+BafA1/1X merge.png]

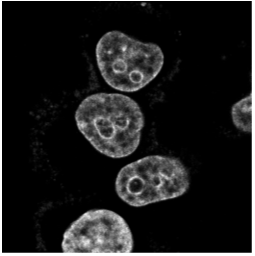

Supplement: Supplementary file 8 — Source data Fig. 6 [file 44319_2025_551_MOESM8_ESM.zip › Fig6/Fig6D/STING-EGFP+BafA1/3X DAPI.png]

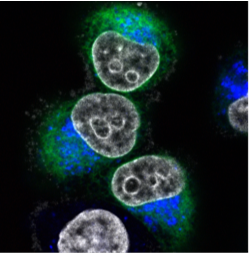

Supplement: Supplementary file 8 — Source data Fig. 6 [file 44319_2025_551_MOESM8_ESM.zip › Fig6/Fig6D/STING-EGFP+BafA1/3X merge.png]

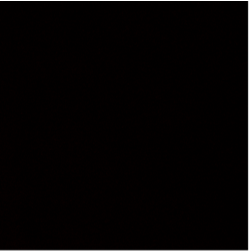

Supplement: Supplementary file 8 — Source data Fig. 6 [file 44319_2025_551_MOESM8_ESM.zip › Fig6/Fig6D/STING-EGFP+BafA1/3X ORF1p.png]

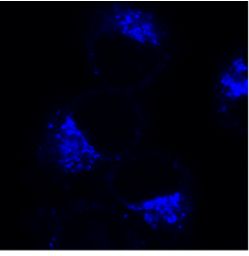

Supplement: Supplementary file 8 — Source data Fig. 6 [file 44319_2025_551_MOESM8_ESM.zip › Fig6/Fig6D/STING-EGFP+BafA1/3X Rab7.png]

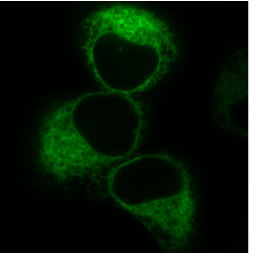

Supplement: Supplementary file 8 — Source data Fig. 6 [file 44319_2025_551_MOESM8_ESM.zip › Fig6/Fig6D/STING-EGFP+BafA1/3X STING.png]

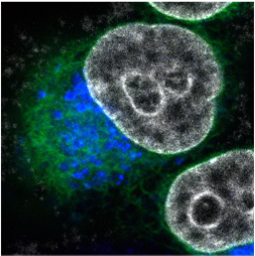

Supplement: Supplementary file 8 — Source data Fig. 6 [file 44319_2025_551_MOESM8_ESM.zip › Fig6/Fig6D/STING-EGFP+BafA1/6X merge.png]

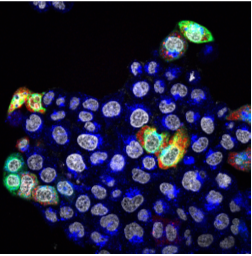

Supplement: Supplementary file 8 — Source data Fig. 6 [file 44319_2025_551_MOESM8_ESM.zip › Fig6/Fig6D/STING-EGFP+L1/1X merge.png]

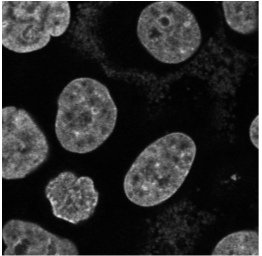

Supplement: Supplementary file 8 — Source data Fig. 6 [file 44319_2025_551_MOESM8_ESM.zip › Fig6/Fig6D/STING-EGFP+L1/3X DAPI.png]

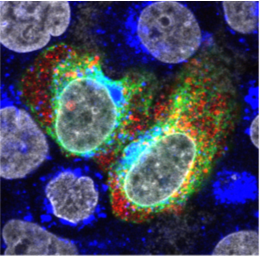

Supplement: Supplementary file 8 — Source data Fig. 6 [file 44319_2025_551_MOESM8_ESM.zip › Fig6/Fig6D/STING-EGFP+L1/3X merge.png]

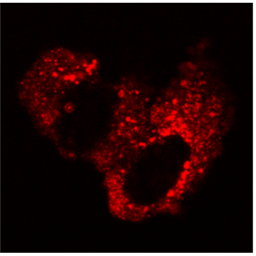

Supplement: Supplementary file 8 — Source data Fig. 6 [file 44319_2025_551_MOESM8_ESM.zip › Fig6/Fig6D/STING-EGFP+L1/3X ORF1p.png]

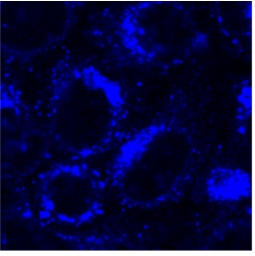

Supplement: Supplementary file 8 — Source data Fig. 6 [file 44319_2025_551_MOESM8_ESM.zip › Fig6/Fig6D/STING-EGFP+L1/3X Rab7.png]

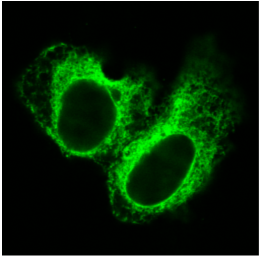

Supplement: Supplementary file 8 — Source data Fig. 6 [file 44319_2025_551_MOESM8_ESM.zip › Fig6/Fig6D/STING-EGFP+L1/3X STING.png]

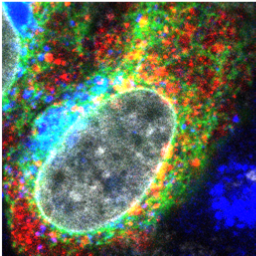

Supplement: Supplementary file 8 — Source data Fig. 6 [file 44319_2025_551_MOESM8_ESM.zip › Fig6/Fig6D/STING-EGFP+L1/6X merge.png]

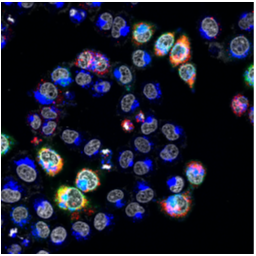

Supplement: Supplementary file 8 — Source data Fig. 6 [file 44319_2025_551_MOESM8_ESM.zip › Fig6/Fig6D/STING-EGFP+L1+BafA1/1X merge.png]

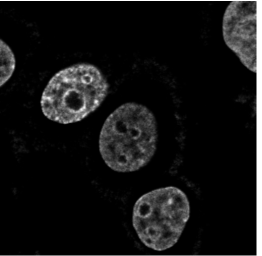

Supplement: Supplementary file 8 — Source data Fig. 6 [file 44319_2025_551_MOESM8_ESM.zip › Fig6/Fig6D/STING-EGFP+L1+BafA1/3X DAPI.png]

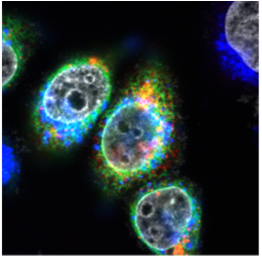

Supplement: Supplementary file 8 — Source data Fig. 6 [file 44319_2025_551_MOESM8_ESM.zip › Fig6/Fig6D/STING-EGFP+L1+BafA1/3X merge.png]

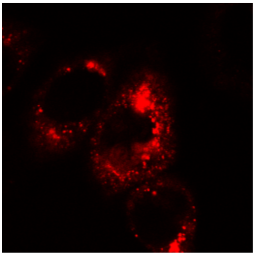

Supplement: Supplementary file 8 — Source data Fig. 6 [file 44319_2025_551_MOESM8_ESM.zip › Fig6/Fig6D/STING-EGFP+L1+BafA1/3X ORF1p.png]

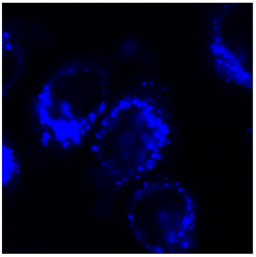

Supplement: Supplementary file 8 — Source data Fig. 6 [file 44319_2025_551_MOESM8_ESM.zip › Fig6/Fig6D/STING-EGFP+L1+BafA1/3X Rab7.png]

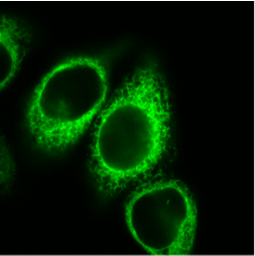

Supplement: Supplementary file 8 — Source data Fig. 6 [file 44319_2025_551_MOESM8_ESM.zip › Fig6/Fig6D/STING-EGFP+L1+BafA1/3X STING.png]

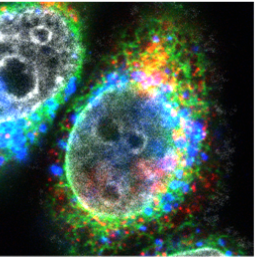

Supplement: Supplementary file 8 — Source data Fig. 6 [file 44319_2025_551_MOESM8_ESM.zip › Fig6/Fig6D/STING-EGFP+L1+BafA1/6X merge.png]

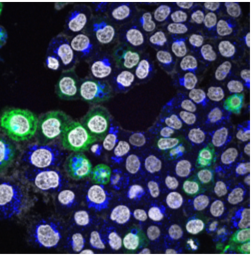

Supplement: Supplementary file 8 — Source data Fig. 6 [file 44319_2025_551_MOESM8_ESM.zip › Fig6/Fig6E/STING-EGFP/1X merge.png]

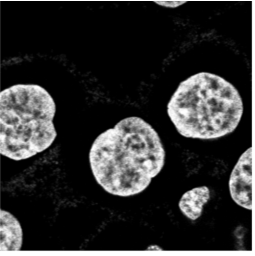

Supplement: Supplementary file 8 — Source data Fig. 6 [file 44319_2025_551_MOESM8_ESM.zip › Fig6/Fig6E/STING-EGFP/3X DAPI.png]

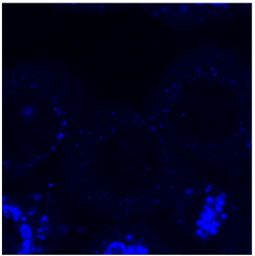

Supplement: Supplementary file 8 — Source data Fig. 6 [file 44319_2025_551_MOESM8_ESM.zip › Fig6/Fig6E/STING-EGFP/3X LAMP1.png]

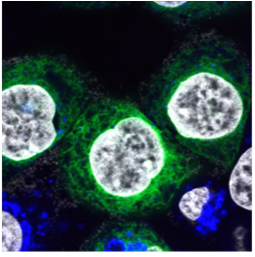

Supplement: Supplementary file 8 — Source data Fig. 6 [file 44319_2025_551_MOESM8_ESM.zip › Fig6/Fig6E/STING-EGFP/3X merge.png]

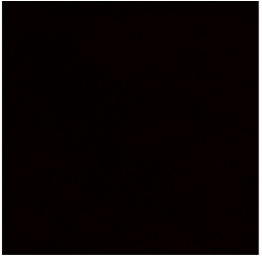

Supplement: Supplementary file 8 — Source data Fig. 6 [file 44319_2025_551_MOESM8_ESM.zip › Fig6/Fig6E/STING-EGFP/3X ORF1p.png]

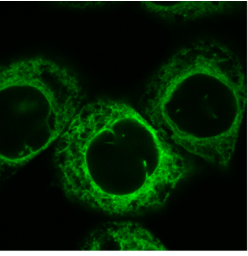

Supplement: Supplementary file 8 — Source data Fig. 6 [file 44319_2025_551_MOESM8_ESM.zip › Fig6/Fig6E/STING-EGFP/3X STING.png]

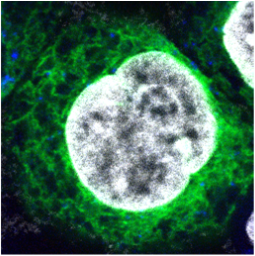

Supplement: Supplementary file 8 — Source data Fig. 6 [file 44319_2025_551_MOESM8_ESM.zip › Fig6/Fig6E/STING-EGFP/6X merge.png]

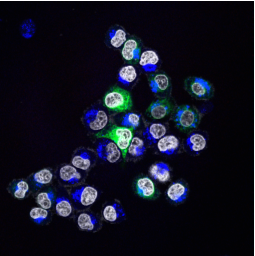

Supplement: Supplementary file 8 — Source data Fig. 6 [file 44319_2025_551_MOESM8_ESM.zip › Fig6/Fig6E/STING-EGFP+BafA1/1X merge.png]

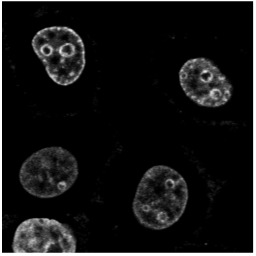

Supplement: Supplementary file 8 — Source data Fig. 6 [file 44319_2025_551_MOESM8_ESM.zip › Fig6/Fig6E/STING-EGFP+BafA1/3X DAPI.png]
