# Supplementary figures and images for: STING inhibits LINE-1 retrotransposition through sorting ORF1p to lysosomes for degradation (part 4 of 4)
Source: EMBO Rep. 2025 Aug 18;26(18):4607–30. doi: 10.1038/s44319-025-00551-0 (PMC12457603; doi:10.1038/s44319-025-00551-0)

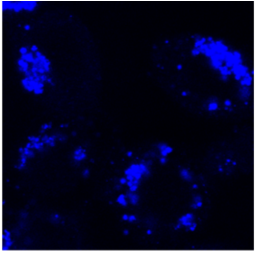

Supplement: Supplementary file 8 — Source data Fig. 6 [file 44319_2025_551_MOESM8_ESM.zip › Fig6/Fig6E/STING-EGFP+BafA1/3X LAMP1.png]

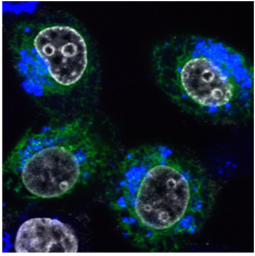

Supplement: Supplementary file 8 — Source data Fig. 6 [file 44319_2025_551_MOESM8_ESM.zip › Fig6/Fig6E/STING-EGFP+BafA1/3X merge.png]

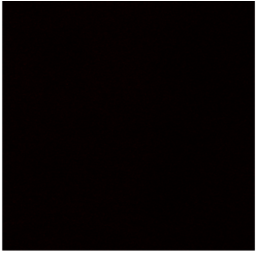

Supplement: Supplementary file 8 — Source data Fig. 6 [file 44319_2025_551_MOESM8_ESM.zip › Fig6/Fig6E/STING-EGFP+BafA1/3X ORF1p.png]

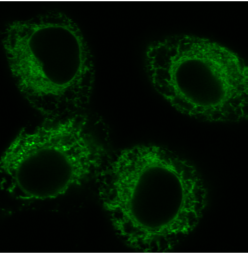

Supplement: Supplementary file 8 — Source data Fig. 6 [file 44319_2025_551_MOESM8_ESM.zip › Fig6/Fig6E/STING-EGFP+BafA1/3X STING.png]

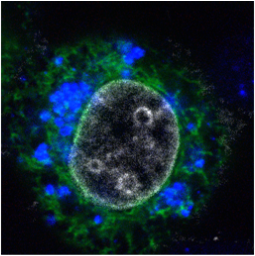

Supplement: Supplementary file 8 — Source data Fig. 6 [file 44319_2025_551_MOESM8_ESM.zip › Fig6/Fig6E/STING-EGFP+BafA1/6X merge.png]

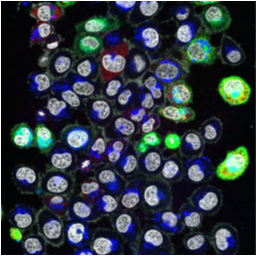

Supplement: Supplementary file 8 — Source data Fig. 6 [file 44319_2025_551_MOESM8_ESM.zip › Fig6/Fig6E/STING-EGFP+L1/1X merge.png]

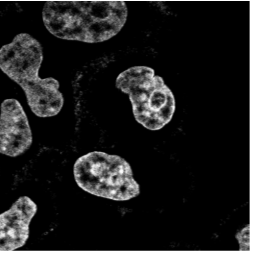

Supplement: Supplementary file 8 — Source data Fig. 6 [file 44319_2025_551_MOESM8_ESM.zip › Fig6/Fig6E/STING-EGFP+L1/3X DAPI.png]

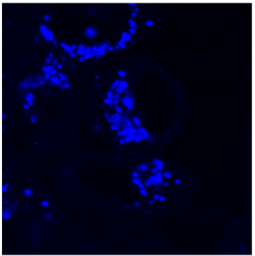

Supplement: Supplementary file 8 — Source data Fig. 6 [file 44319_2025_551_MOESM8_ESM.zip › Fig6/Fig6E/STING-EGFP+L1/3X LAMP1.png]

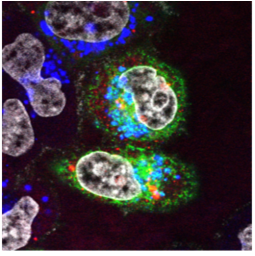

Supplement: Supplementary file 8 — Source data Fig. 6 [file 44319_2025_551_MOESM8_ESM.zip › Fig6/Fig6E/STING-EGFP+L1/3X merge.png]

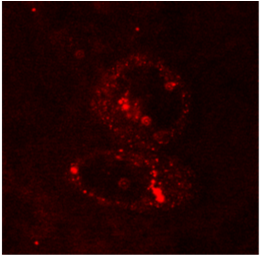

Supplement: Supplementary file 8 — Source data Fig. 6 [file 44319_2025_551_MOESM8_ESM.zip › Fig6/Fig6E/STING-EGFP+L1/3X ORF1p.png]

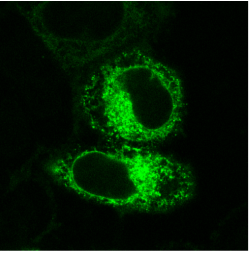

Supplement: Supplementary file 8 — Source data Fig. 6 [file 44319_2025_551_MOESM8_ESM.zip › Fig6/Fig6E/STING-EGFP+L1/3X STING.png]

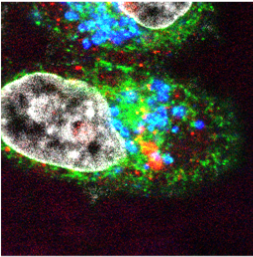

Supplement: Supplementary file 8 — Source data Fig. 6 [file 44319_2025_551_MOESM8_ESM.zip › Fig6/Fig6E/STING-EGFP+L1/6X merge.png]

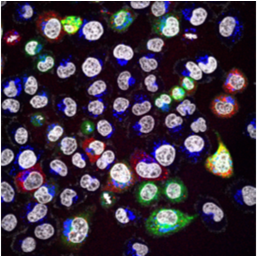

Supplement: Supplementary file 8 — Source data Fig. 6 [file 44319_2025_551_MOESM8_ESM.zip › Fig6/Fig6E/STING-EGFP+L1+BafA1/1X merge.png]

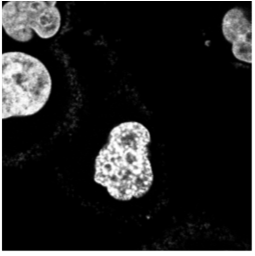

Supplement: Supplementary file 8 — Source data Fig. 6 [file 44319_2025_551_MOESM8_ESM.zip › Fig6/Fig6E/STING-EGFP+L1+BafA1/3X DAPI.png]

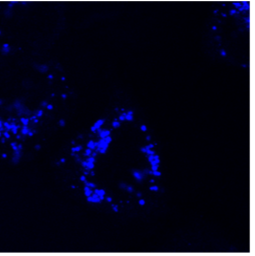

Supplement: Supplementary file 8 — Source data Fig. 6 [file 44319_2025_551_MOESM8_ESM.zip › Fig6/Fig6E/STING-EGFP+L1+BafA1/3X LAMP1.png]

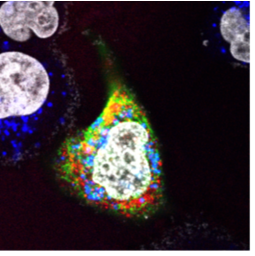

Supplement: Supplementary file 8 — Source data Fig. 6 [file 44319_2025_551_MOESM8_ESM.zip › Fig6/Fig6E/STING-EGFP+L1+BafA1/3X merge.png]

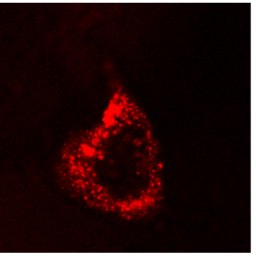

Supplement: Supplementary file 8 — Source data Fig. 6 [file 44319_2025_551_MOESM8_ESM.zip › Fig6/Fig6E/STING-EGFP+L1+BafA1/3X ORF1p.png]

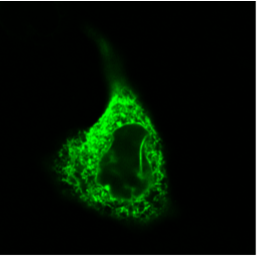

Supplement: Supplementary file 8 — Source data Fig. 6 [file 44319_2025_551_MOESM8_ESM.zip › Fig6/Fig6E/STING-EGFP+L1+BafA1/3X STING.png]

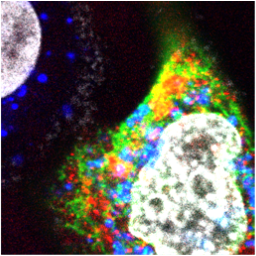

Supplement: Supplementary file 8 — Source data Fig. 6 [file 44319_2025_551_MOESM8_ESM.zip › Fig6/Fig6E/STING-EGFP+L1+BafA1/6X merge.png]
