# Supplementary material for: STING inhibits LINE-1 retrotransposition through sorting ORF1p to lysosomes for degradation
Source: EMBO Rep. 2025 Aug 18;26(18):4607–30. doi: 10.1038/s44319-025-00551-0 (PMC12457603; doi:10.1038/s44319-025-00551-0)
Supplement: Supplementary file 9 — Expanded View Figures [file 44319_2025_551_MOESM9_ESM.pdf]

Expanded View Figures

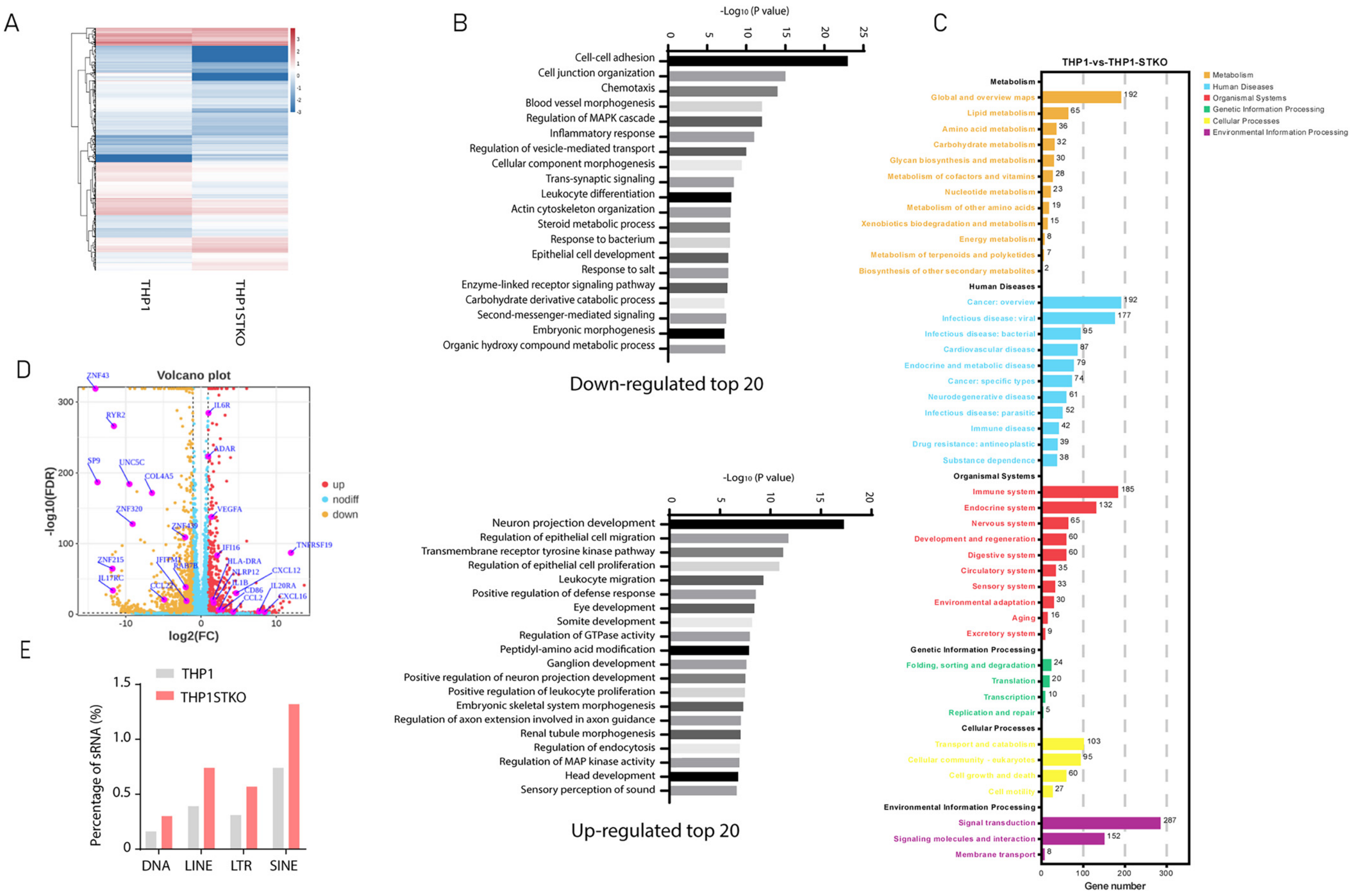

Figure EV1. RNA sequencing analysis of DEGs and clustering between control and STING-knockout THP1 cells.

(**A**) Expression heatmap of THP1 control and STING-knockout cells. (**B**) Bar graphs representing enriched top 20 GO BPs of upregulated and downregulated DEGs in STING-knockout THP1 cells with expression log2 [FC] >1 and $P < 0.05$, generated by Metascape. The *P* value was calculated based on the hypergeometric test. (**C**) Kyoto Encyclopedia of Genes and Genomes (KEGG) pathways of DEGs between control and STING-knockout THP1 cells. (**D**) Volcano plot of DEGs between control and STING knockout THP1 cells. The names of the DEGs associated with inflammation and immunity are shown. (**E**) Analysis of small RNA (18 to 30 nt) in STING-knockout THP1 cells.

© The Author(s)

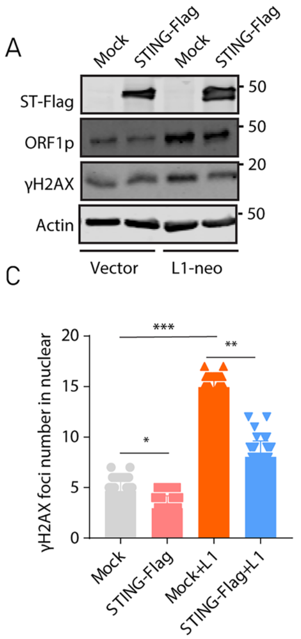

Figure EV2. STING inhibits the formation of nuclear γH2AX foci induced by L1 retrotransposition.

(**A**) Western blots to measure the levels of γH2AX in HeLa cells, which stably express STING-Flag and were transfected with the CMV-L1-neo^{RT} DNA for 48 h. (**B**, **C**) Detection of γH2AX foci in HeLa cells which stably express STING-Flag and were transfected with the CMV-L1-neo^{RT} DNA. Immunofluorescence was performed 24 h post transfection (**B**). Scale bar, 10 µm. γH2AX foci were scored in more than 50 cells for each treatment. The average number of γH2AX foci per cell is presented in the bar graph (**C**) (mean ± SEM; paired *t*-test). There was a significant difference ($p = 0.023$) in the STING-Flag stably-expressing cell line compared with the mock cell lines; a significant difference ($p < 0.001$) was also observed in the mock cell line with L1 transfection compared with the mock cell line; a significant difference ($p = 0.003$) was observed in STING-Flag stably-expressing cell line compared with the mock cell line which was transfected with L1 reporter system. ns non-significant; *$p < 0.05$; **$p < 0.01$; ***$p < 0.001$.

 © The Author(s)

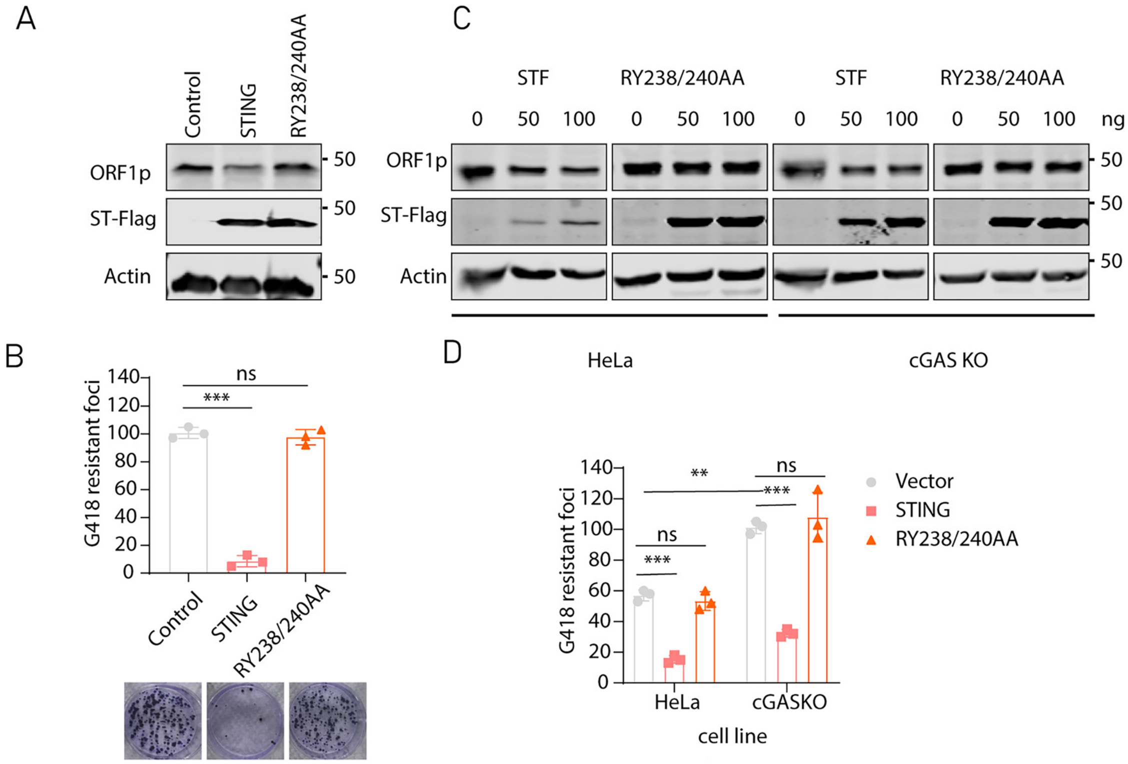

Figure EV3. STING RY238/240AA mutation impairs its ability to inhibit LINE-1 independently of cGAMP.

(**A**) Levels of endogenous ORF1p were determined in HeLa cells stably expressing STING WT or RY238/240AA mutant. (**B**) CMV-L1-neo^{RT} colony assay performed in HeLa cells stably expressing STING WT or RY238/240AA. The results of three independent experiments are presented in the bar graphs (mean ± SEM; paired *t*-test). Compared with the control group, a significant difference was observed in the STING-Flag stably-expressing cell lines ($p < 0.001$), but not in the RY238/240AA mutant stably-expressing cell lines ($p = 0.716$). ns, non-significant; *$p < 0.05$; **$p < 0.01$; ***$p < 0.001$. (**C**) STING WT or RY238/240AA mutant were co-transfected with CMV-L1-neo^{RT} into HeLa or cGAS knockout cells. Levels of ORF1p were determined 48 h post transfection. (**D**) CMV-L1-neo^{RT} colony assay performed with co-transfected STING WT or RY238/240AA mutant in cGAS knockout or control HeLa cells. The results of three independent experiments are presented in the bar graphs (mean ± SEM; paired *t*-test). In the control cell line, a significant difference was observed in the STING-Flag stably-expressing cell lines ($p < 0.001$), but not in the RY238/240AA mutant stably-expressing cell lines ($p = 0.807$); in the cGASKO cell line, there was also a significant difference in the STING-Flag stably-expressing cell lines ($p < 0.001$), but not in the RY238/240AA mutant stably-expressing cell lines ($p = 0.508$). ns non-significant; *$p < 0.05$; **$p < 0.01$; ***$p < 0.001$.

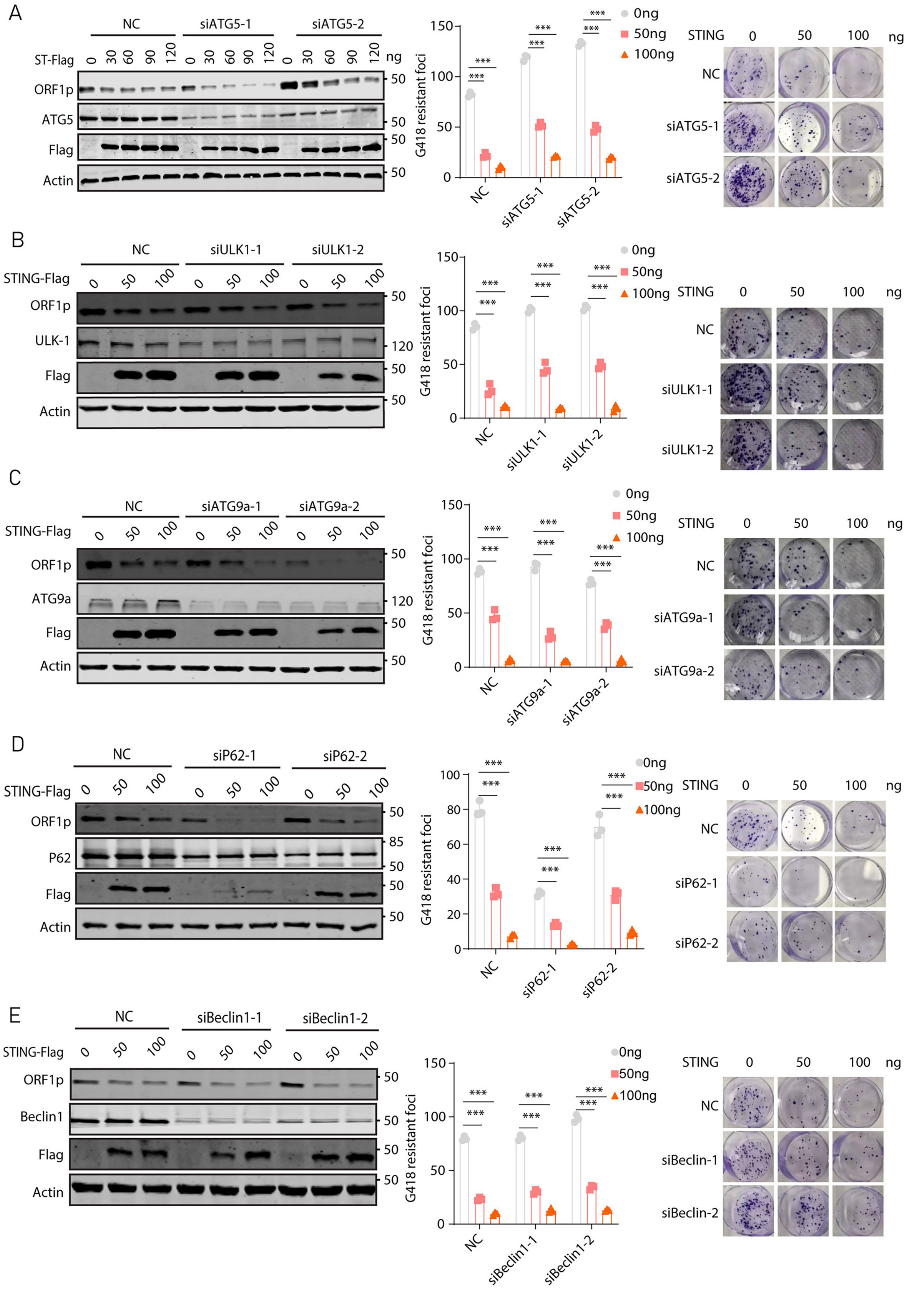
A
NC
siATG5-1
siATG5-2
ST-Flag
0 30 60 90 120 0 30 60 90 120 0 30 60 90 120 ng
ORF1p
ATG5
Flag
Actin
50
50
50
50
G418 resistant foci
150
100
50
0
***
0ng
50ng
100ng
NC
siATG5-1
siATG5-2
STING 0 50 100 ng
B
NC
siULK1-1
siULK1-2
STING-Flag
0 50 100 0 50 100 0 50 100
ORF1p
ULK-1
Flag
Actin
50
120
50
50
siULK1-1
siULK1-2
C
NC
siATG9a-1
siATG9a-2
ATG9a
siATG9a-1
siATG9a-2
D
NC
siP62-1
siP62-2
P62
85
80
60
40
20
siP62-1
siP62-2
E
NC
siBeclin1-1
siBeclin1-2
Beclin1
siBeclin1-1
siBeclin1-2
siBeclin-1
siBeclin-2

 © The Author(s)

Figure EV4. Knockdown of autophagy factors does not affect STING inhibition of LINE-1.

(**A–E**) ATG5 (**A**), ULK1 (**B**), ATG9a (**C**), P62 (**D**), or Beclin1 (**E**) was knocked down in HeLa cells with siRNA. followed by co-transfection with STING and CMV-L1-neo^{RT} DNA. ORF1p was detected with Western blots at 48 h post transfection. A colony assay was performed to measure LINE-1 activity. The results of three independent experiments are presented in the bar graphs (mean ± SEM; paired *t*-test). Compared with 0 ng STING-Flag transfection, significant differences were observed at 50 ng ($p < 0.001$) and 100 ng ($p < 0.001$), independent of ATG5, ULK1, ATG9a, P62, or Beclin1 knockdown. ns non-significant; *$p < 0.05$; **$p < 0.01$; ***$p < 0.001$.

© The Author(s)

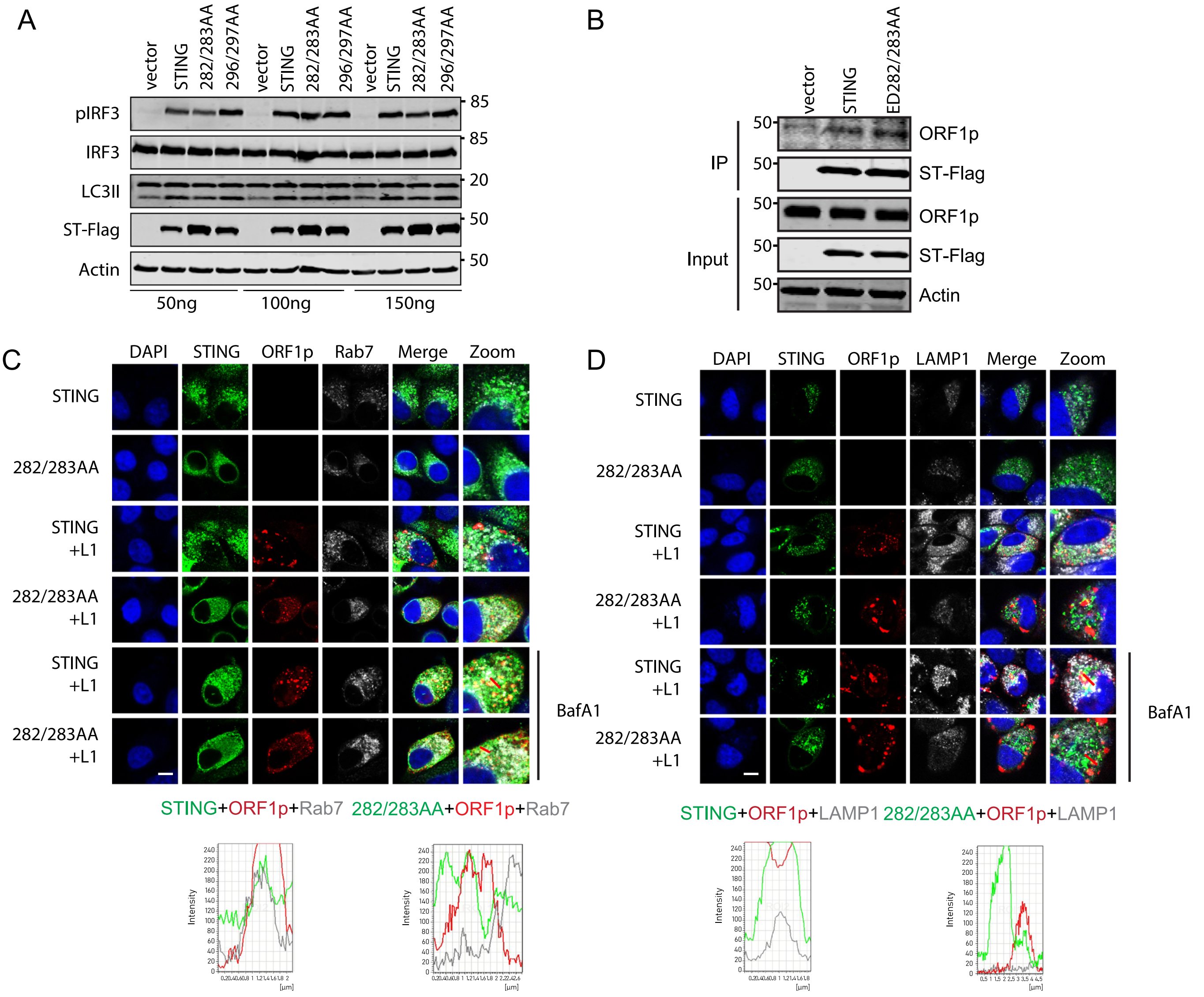

Figure EV5. Amino acids E282/D283 of STING are required for trafficking of STING/ORF1p complex to lysosomes.

(**A**) HeLa cells were transfected with STING and its mutants. Western blots were performed to detect IRF3 phosphorylation and LC3 lipidation. (**B**) HEK293 cells were co-transfected with CMV-L1-neo^{RT} DNA and STING-Flag or its mutants. Immunoprecipitation was performed with anti-Flag antibody 48 h post transfection. Presence of ORF1p in the precipitated materials was detected by Western blots. (**C**) Colocalization of STING-EGFP or its E282A/D283A mutant with ORF1p and Rab7 in HeLa cells treated with BafA1. STING in Green, ORF1p in red, and Rab7 in gray. ImageJ was used to analyze the colocalization. Scale bar, 10 μm. (**D**) Colocalization of STING-EGFP or its E282A/D283A mutant with ORF1p and LAMP1 in HeLa cells treated with BafA1. STING in Green, ORF1p in red, and LAMP1 in gray. Red Line indicates colocalization. ImageJ was used to analyze the colocalization. Scale bar, 10 μm.

 © The Author(s)
